# *Aspergillus fumigatus* from Pathogenic Fungus to Unexplored Natural Treasure: Changing the Concept

**DOI:** 10.4014/jmb.2411.11082

**Published:** 2025-03-11

**Authors:** Sabrin R.M. Ibrahim, Hagar Mohamed Mohamed, Anfal S. Aljahdali, Samar S. A. Murshid, Shaimaa G. A. Mohamed, Hossam M. Abdallah, Gamal A. Mohamed

**Affiliations:** 1 Department of Chemistry, Preparatory Year Program, Batterjee Medical College, Jeddah 21442, Saudi Arabia; 2 Department of Pharmacognosy, Faculty of Pharmacy, Assiut University, Assiut 71526, Egypt; 3 Department of Medical Laboratory Analysis, Faculty of Medical & Health Sciences, Liwa College, Abu Dhabi 41009, United Arab Emirates; 4 Department of Applied Medical Chemistry, Medical Research Institute, Alexandria University, Alexandria, Egypt; 5 Department of Pharmaceutical Chemistry, Faculty of Pharmacy, King Abdulaziz University, Jeddah, Saudi Arabia; 6 Department of Natural Products and Alternative Medicine, Faculty of Pharmacy, King Abdulaziz University, Jeddah 21589, Saudi Arabia; 7 Department of Fixed Prosthodontics, Faculty of Dentistry, The British University in Egypt, Cairo, 11837, Egypt; 8 Department of Pharmacognosy, Faculty of Pharmacy, Cairo University, Cairo 11562, Egypt

**Keywords:** Fungi, *Aspergillus fumigatus*, alkaloids, biological functions, biosynthesis, life on land

## Abstract

The *Aspergillus* genus is one of the oldest known fungal genera. Its species can survive in extremely difficult environmental conditions and can affect plants, animals, and human health due to their pathogenic characteristics. Also, they represent an enormous chemical and biological repertoire. *A. fumigatus* is a fungal pathogen that has been thoroughly investigated due to its medical significance. Additionally, *A. fumigatus* has revealed a capacity to biosynthesize different classes of secondary metabolites such as alkaloids, terpenes, sterols, quinones, peptides, and benzophenones. Alkaloids are the most common type of secondary metabolites that *A. fumigatus* produces, and they have a variety of biological functions. This current study aimed to highlight the positive aspects of this fungus. The published research on the structures, biological functions, and biosynthesis of *A. fumigatus*'s isolated metabolites were emphasized in this work. The current review comprised more than 400 compounds, with more than 145 references that were published between 1965 and August 2024. This review underscores the role of this fungus as a reservoir of bioactive compounds with antimicrobial, anticancer, anti-inflammatory, and agricultural applications.

## Introduction

Fungi are substantial microorganisms that negatively and positively influence human life in different ways and have principal ecological roles. For example, fungi are important plant symbionts (mycorrhiza), key contributors in saprobic decompositions, widely used enzyme producers, and human, animal, and plant pathogens, in addition, they are important in feed, food preservation, and food production [1]. Additionally, fungi biosynthesize a remarkable diversity of secondary metabolites that improve fungi competitiveness versus other microbes and aid adaptation to various environmental conditions [2]. These secondary metabolites have substantial roles in fungal communication, defense, and virulence and some of them are toxic (mycotoxins) and proved as one of the wealthiest fields for pharmaceutical discovery [3].

*Aspergillus* genus (Aspergillaceae family, Eurotiales order) is one of the most ubiquitous and biggest fungal genera, including different sections: sect. *Aenei*, *Cervini*, *Aspergillus*, *Clavati*, *Flavi*, *Fumigati*, *Flavipedes*, *Nidulantes*, *Polypaecilum*, *Nigri*, *Circumdati*, *Terrei*, *Restricti*, and *Usti* [4] with common species such as *A. versicolor*, *A. fumigatus*, *A. ustus*, *A. flavus*, and *A. sydowii*. *Aspergillus* species have been renowned as a valuable resource for food, agrochemicals, pharmaceuticals, and enzymes [5-9]. This species produces diverse and unique types of secondary metabolites such as alkaloids, terpenes, steroids, polyketides, and quinones, as well as enzymes with substantial biotechnological applications [5-10].

*A. fumigatus* is the most prevalent species of this genus and is commonly found in air, soil, and organic materials such as malted barley, cereals, compost, tobacco, and packed hay [4, 11-13]. This fungus has a substantial contribution to the environmental recycling of nitrogen and carbon [12]. This fungus has high adaptability and can colonize diverse environments due to its metabolic diversity and broad thermal and stress tolerances, as well as the easy spread of its conidia [14]. *A. fumigatus* is reported as one of the most prevalent airborne pathogens to humans that can produce many infections in both immunocompetent and immunocompromised patients [4]. It was estimated that there are 16 million pulmonary infections annually with fatal impacts on many hundred thousand patients [4]. It causes severe and fatal invasive infection in immunocompromised patients, known as invasive aspergillosis because of the inhalation of its conidia that leads to a high mortality rate of 40%-90% [11, 12, 14]. Various cases of neurotropic and tremorgenic intoxication were reported due to ingesting *A. fumigatus*, growing in homemade “miso” and rice [15]. *A. fumigatus* virulence is associated with its capability to yield a variety of toxic metabolites [16]. It was reported that fumagillin was among the potent antibiotics that was isolated from this fungus and employed in apiculture and microsporidian infections [17]. Also, it has a significant role in *A. fumigatus* fast adaptation to various stressors, such as contact with the host's lung tissue and immune system, which favors the infections [14].

On the other hand, *A. fumigatus* yields diverse types of metabolites such as alkaloids, terpenes, sterols, peptides, chromane, anhydride, quinones, and azophilane derivatives with an array of bioactivities. Additionally, this fungus was reported to secrete different enzymes: cellulase, xylanase, β-glucosidase, monooxygenase, amylase, amyloglucosidase, galactanase, proteinase, keratinase, elastase, and lipase that could have wide applications in various fields.

Many published reviews have focused on the pathogenesis and diseases caused by this fungus, as well as its clinical significance and drug resistance [4, 11, 12, 18-25]. However, no available details review discusses the secondary metabolites and biological properties of this fungus. Therefore, this work attempts to shed light on the bright side of *A. fumigatus*, including the reported secondary metabolites, their biosynthesis, and biological properties (Tables S1-S13).

The data collection for this review was done based on an online search through Scopus, PubMed, Google Scholar, ScienceDirect, and different publishers (*e.g.*, Wiley, Taylor & Francis, MDPI, ACS, RSC, Betham, and Thieme) websites between 1965 and August 2024. The search was carried out using keywords: *A. fumigatus* + Compounds, OR *A. fumigatus* + metabolites, OR *A. fumigatus* + Biosynthesis, OR *A. fumigatus* + biological activity. All published works related to *A. fumigatus*, its metabolites, biological activity, applications, and enzymes published in English were included. For non-English published articles, the data were obtained from their English abstract, however, full non-English written articles, as well as the articles dealing with the pathogenic effects of *A. fumigatus* were not included.

## Aspergillus Fumigatus Secondary Metabolites

### Alkaloids

**Fig. 1:**
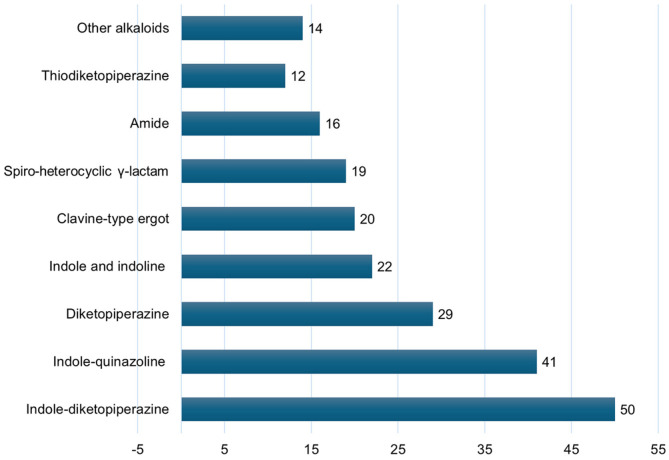
Classes of alkaloids reported from *A. fumigatus*.

Alkaloids represent about 55% (222 compounds) of the metabolites reported from *A. fumigatus*. These alkaloids are categorized according to their structural core into indole-diketopiperazine, indole-quinazoline, diketopiperazine, clavine-type ergot, spiro-heterocyclic γ-lactam, indole and indoline, amide, thiodiketopiperazine, and other alkaloids (Tables S1-S4) (Fig. 1). Among them, indole-diketopiperazine and indole-quinazoline represent the major alkaloids. It was noted that indole moiety is involved in the skeleton of many of these alkaloids. Below, the different classes of alkaloids were represented with their isolation, characterization, structural features, and bioactivities (Table S13).

**Fig. 2:**
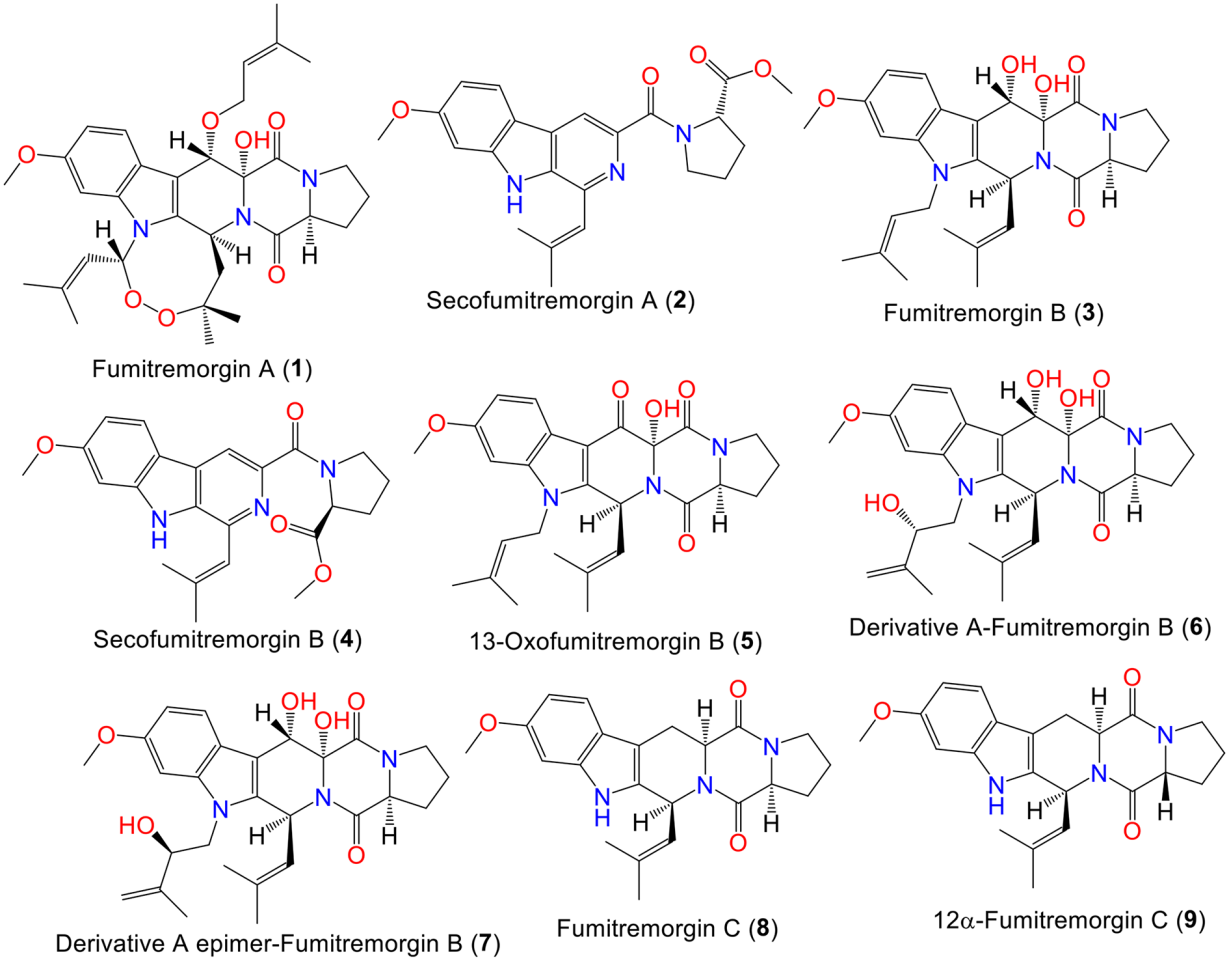
Examples of indole-diketopiperazine alkaloids (1–9) reported from *A. fumigatus*.

**Indole-diketopiperazine alkaloids.** The reported indole-diketopiperazine from *A. fumigatus* were listed in Table S1 [26-64]. Compounds **1** and **3**, new tremorgenic toxins were separated from *A. fumigatus* Fres. mycelia EtOAc extract by SiO_2_CC and repeated crystallization. They were characterized by NMR/Xray/chemical derivatization methods, as well as optical rotation measurement. Both compounds feature a 7-methoxytetrahydrocarbazole skeleton, whereas **1** has two oxygen and isopentenyl moieties more than **3** [26, 27] (Fig. 2). It was found that **1** frequently produced death in mice, rats, and rabbits following a severe toxic-clonic convulsion and induced a persistent tremor with intermittent convulsions. Additionally, **1** and **3** significantly stimulated the central nervous system. Yamazaki *et al*. found that giving **1** to mice (half maximal lethal concentration (LD_50_) 185 μg/kg, IV) substantially boosted and diminished the serotonin and g-aminobutyric acid (GABA) levels, respectively in their brains [27]. Interestingly, these metabolites did not possess tremorgenic effects in mice [27, 28].

Compound **5** (minimum inhibitory concentration (MIC) 1.25 μM) displayed marked antibacterial efficacy versus MRSA, compared to vancomycin (MIC 1.0 μM), whilst **10** also possessed powerful efficacy versus *M. bovis* (MIC 25.0 μM) and **3** was moderately active versus *C. albicans* (MIC 50.0 μM) [39]. The new metabolites, **2** and **4**, together with **8**, **10**, and **29** were identified from sea sediment-derived SD-406 strain using spectral/chiral HPLC/X-ray/J-based configuration analyses, as well as quantum chemical calculations [29]. Among them, **2/4** were obtained as an inseparable mixture that are derived from the indole-diketopiperazine derivative, **8** by cleaving the amide bond followed by pyridine ring formation through aromatization. Their antimicrobial assay against different aquatic, plant, and human pathogens revealed that **29** was effective versus *F. graminearum* (MIC 64.0 μg/ml) and **2/4** exhibited potential against *F. graminearum* (plant pathogen) and *Edwardsiella tarda* and *Vibrio alginolyticus* (aquatic pathogens) (MICs 4.0, 64.0, and 32.0 μg/ml, respectively), however, **8** and **10** had no notable effect [29]. On the other hand, **3** and **8** reported from *A. fumigatus* AR05 associated with *Astragalus membranaceus* root, demonstrated marked antifungal and antibacterial properties (MICs 4–64 μg/ml) towards *B. subtilis*, *S. aureus*, *E. coli*, *S. typhimurium*, *C. albicans*, *P. chrysogenum*, and *F. solani* [36]. Further, the EtOAc extract of *A. fumigatus* accompanied with *Crocus sativus* lateral buds and its metabolite (**8**) possessed moderate inhibition potential versus *Erwinia* sp. (MIC 100 μg/ml) [38]. In *in vitro* antiparasitic assay, 8 was moderately active against *P. falciparum* and *T. cruzi* (half-maximal inhibitory concentrations (IC_50_s) 2.3 and 9.6 μM, respectively), compared to chloroquine and benznidazole (IC_50_s 0.017 and 2.6 μM, respectively) [49]. Also, Abraham and Arfmann reported the separation of new fumitremorgin-related analog: **10** from *A. fumigatus* Fres. DSM-790 culture by SiO_2_ and reversed phase-18 column chromatography (RP-18 CC) that was identified by spectral techniques to have a relative configuration 3S/12R/13S [15] (Fig. S1).

Additionally, **15**–**17** and **44**, and the known **8**, **10**, **22**, and **27**, isolated from *Erythrophloeum fordii* stem-accompanied *A. fumigatus* were determined by spectral/CD (circular dichroism) experiments. The assessing of their anti-inflammatory effect using b-glucuronidase release inhibitory ratio in rat polymorphonuclear leukocytes (PMNs) caused by the platelet-activating factor (PAF) revealed that these compounds had weak activity with inhibitory ratios ranging from 6.1% to 20.2% (Conc. 10 μM), compared to ginkgolide B (inhibitory ratio 80.5%; Conc. 10 μM), while they had no activity versus ileocecal colorectal adenocarcinoma (HCT-8), hepatocellular carcinoma (Bel-7420), gastric carcinoma (BGC-823), lung adenocarcinoma epithelial (A-549), ovarian cancer (A2780), and ovarian cancer (A2780) in the 3-(4,5-dimethylthiazol-2-yl)-2,5-diphenyltetrazolium bromide (MTT) assay [43].

Liang *et al*. reported the isolation and characterization of a new alkaloid, **19**, together with **8**, **10**, **37**, and **38** from *A. fumigatus* Wrq12 [44]. Compounds **10**, **19**, and **37** showed cytotoxic effect on hepatocellular carcinoma (HepG2) cell line (IC_50_ 4.5, 47.5, and 9.8 μM, respectively), while **8** and **38** had IC_50_ values of 156.5 and 44.9 μM, respectively, suggesting that the C-12 and C-13 hydroxyls are substantial for indole-diketopiperazine alkaloids cytotoxic activity versus HepG2 cell line [44].

Compounds **18** and **20** are pair of atrop-isomers that were isolated from the EtOAc extract GXIMD00544 derived from saltern using silica gel column chromatography (SiO_2_ CC)/Rp-18CC/HPLC, along with **8, 27, 35, 36**, and **39**. The structures of **18** and **20** were elucidated by spectral/chemical reaction/quantum chemical calculations. Compounds **18/20** were like **2** and **4** previously reported by Yan *et al*. The main difference among them is the absence of C-17-OCH_3_ in **18/20** (Fig. S2). Compounds **18/20** originated through C_11_-C_12_ bond rotation between isopentenyl and proline units as in **2** and **4** that have 6S-configuration based on Marfey’s reaction products. Compound **18** demonstrated inhibition against *Fusarium* sp. associated with sugarcane (inhibitory rate 53%, Conc. 100 μM). Further, atrop-isomers **18/20** possessed antifouling capacity against *Balanus amphitrite* larval settlement (Conc. 100 μM; inhibitory rate 96%) [51].

New indole-diketopiperazines: **12**, **21**, and **27** and the known analogs, **3**, **8**, **10**, and **37** were separated by Cui *et al*. from sea-sediment associated *A. fumigatus* BM939 using SiO_2_ CC/preparative thin layer chromatography (TLC)/HPLC (Fig. S3). The structures of **21** and **27** include proline and two isoprenyl tryptophan moieties [30, 77]. These diketopiperazines exhibited a cell cycle progression inhibitory capacity of mouse tsFT210 cells at M-phase (MICs 0.45–60.8 μM) in the flow cytometry assay [30, 77]. Compound **23**, an indole diketopiperazine analog with a tricyclic 5/6/5 framework features a dipyrrolo[1,2-*a*:1’,2’-*d*]pyrazine-5,10-dione ring with a prenylated indole moiety A that was assigned by spectral/Xray analyses [56]. This compound was proposed to be biosynthesized from L-Pro and L-Trp via indole diketopiperazine. Compound **23** was moderately cytotoxic (IC_50_ 14.6 μM) against A-549 cell line in the MTT assay [56].

Compounds **14**, **25**, and **26** are novel indole diketopiperazine alkaloids obtained using D101 macroporous resin/silica gel CC/RP-18 CC/HPLC from *Vaccinium dunalianum* leaf-associated VDL36 strain [55]. Their configurations were assigned as 3S/6S/13S, 3S/6S/12S/13S, and 3S/6S/13S, respectively [55]. Compounds **25** (MICs 7.8–15.62 μg/ml) revealed more potent antifungal efficacy than **26** and **14** (MICs15.62–31.25 μg/ml) on *F. solani*, *F. oxysporum*, and *C. versicolor* [55]. Further, **25** revealed notable *in vivo* curative and protective efficacy against *Botrytis cinerea*, the causative pathogen of tomato gray mold [55].

In 1997, Cui *et al*. identified new indole-diketopiperazines: **24, 29, 32**, and **34** with mammalian cell cycle inhibitory potential were separated and identified from *A. fumigatus* BM939. Compounds **24, 29, 32**, and **34** inhibited the cell cycle progression at the G2/M phase in tsFT210 cells (IC_50_s 5.6–25.3 μM). It is noteworthy that **32** (IC_50_ 23.4 μM) had stronger activity than **10** (its 18-methoxy derivative, IC_50_ 60.8 μM), whereas **24** that is 12-epimer of **10** had pronounced activity (IC_50_ 5.6 μM) than **10** and **32** (IC_50_ 23.4 μM) that differs in C12 configuration and lacks the methoxy at C-18. On the other side, the substituent at C-13 demonstrated little effect on activity (*e.g.*, **32** (IC_50_ 23.4 μM) vs **34** (IC_50_ 25.3 μM)). These findings revealed that C-12 configuration in **24, 29, 32**, and **34** had a marked role in the activity, while the methoxy group on the benzene ring negatively affected the activity [57]. Moreover, **29** and **37** exhibited potent activity versus *B. subtilis*, *S. aureus*, *E. coli*, and *S. typhimurium* (MICs 1–4 μg/ml), compared to gentamicin (MICs 0.5–1.0 μg/ml) [36]. Besides, **37** prohibited the *Caenorhabditis elegans* egg hatching (IC_50_ 2.5 μM) [39].

The new diketopiperazines: **11, 28, 30**, and **31**, along with **3, 8, 10, 24, 29**, and **37** isolated from marine-obtained *A. fumigatus* YK-7 were assessed for cytotoxic effectiveness against human leukemic monocyte lymphoma (U937) and human prostate cancer (PC-3) cell lines using the MTT assay. Compounds **3, 8, 10, 11, 24, 28–31**, and **37** were potent cytotoxic against U937 and PC-3 cell lines (IC_50_s 1.8–6.6 μM), while **3, 11**, and **30** (IC_50_s 14.1, 18.2, and 25.3 μM, respectively) were moderately active versus U937 cell line, compared to doxorubicin HCl (IC_50_s 0.021 and 0.73 μM, respectively). It was noted that maximum activity was observed in C-9 OH substituted compounds. Whilst the formation of C_20_ and C_23_ peroxo bridge (*e.g.*, **3, 30**, and **37**) lowered the activity [33].

Bioassay-guided separation of the extract of *Heteroscyphus tener* liverwort-associated *A. fumigatus* that displayed powerful cytotoxic potential against PC3 cell line (IC_50_ 16.72 mg/ml) afforded new alkaloid: **35**, in addition to **8, 10, 12, 31, 32**, and **39** that were elucidated based on spectral/CD analyses and optical rotations [45]. Compound **35** belongs to indole-diketopiperazines, having N1-attached 3-methylbut-2-enoate moiety that is structurally similar to **39**. Its 3S/6S/12R/13S configuration was assigned based on nuclear Overhauser effect spectroscopy (NOESY)/CD/optical rotation ([α]_D_ -45.5) [45]. These metabolites had a weak cytotoxic effect against the PC3 cell line (IC_50_s 26.6–38.9 μM) [45].

In 2012, new alkaloids, **40** and **41** were reported from *A. fumigatus* LN-4 isolated from *Melia azedarach* stem bark using RP-18/SiO_2_/Sephadex LH-20 CC [34]. Compound **40** features the same skeleton as **24** except having 2-methylpropan-2-ol at C-3 instead of the isobutene group in **24**, while **41** is the methylated analog of **40** [34] (Fig. S4).

Compounds **3, 37**, and **41** exhibited antifungal capacities versus *B. cinerea*, *A. solani*, *A. alternata*, *C. gloeosporioides*, *F. solani*, *F. oxysporum* f. sp. *niveum*, *F. oxysporum* f. sp. *vasinfectum*, and *G. saubinettii* (MICs 6.25–50.0 μg/ml), compared to carbendazim and hymexazol. On the other hand, exerted **3** and **37** displayed notable toxicity in the brine shrimp assay (lethal concentrations 50 (LC_50_s) 13.6 and 15.8 μg/ml, respectively) and antifeedant activity (AFI (antifeedant index) 50.0 and 55.0%, respectively) against armyworm larvae [34]. It was noted that the methoxy group introduction at C-13 resulted in higher activity (*e.g.*, **29** and **41** vs **24** and **40**), regardless of the 12-OH configuration. Compound **41** (MIC 6.25 μg/ml) with a C_3_ 2-methylpropan-2-ol group had more significant activity than **29** with C_3_ isobutenyl (MIC 12.5 μg/ml) versus *A. solani*, *B. cinerea*, *A. alternata*, *C. gloeosporioides*, and *G. saubinetti*, suggesting the C-3 2-methylpropan-2-ol moiety on ring C as in **39, 40**, and **41** is necessary for activity [34]. Compound **37** with peroxide bridge, was 2-fold more active (MICs 6.25, 50.0, 12.5, 25, and 6.25 μg/ ml, respectively) than **3** towards plant pathogens: *C. gloeosporioides*, *F. solani*, *F. oxysporum* f. sp. *niveum*, *F. oxysporum* f. sp. *vasinfectum*, and *G. saubinettii*, indicating the significance of the peroxide bridge for antifungal activity [34]. Another study by Rateb *et al*. revealed that **3** and **37** displayed powerful growth inhibitory activity versus *T. brucei brucei* and *L. donovani* (half maximal effective concentrations (EC_50_s) 0.2/0.2 μM and 3.1/3.9 μM, respectively) with notable toxicity (EC_50_s 0.3 and 0.6 μM, respectively) towards MRC5 cells (normal human lung fibroblasts) [35].

Another study on the BM939 strain by Cui *et al*. reported the new metabolites: **42** and **45** that feature a unique skeleton with a spiro ring system, containing a five-membered hetero ring fused to a diketopiperazine moiety and a benzene ring fused to g-lactam that are formed from a proline, tryptophan, and isoprenyl moieties [60, 78]. The tryptophan moiety is modified by dihydrogenation and further oxidation at C3/C2 and C2, respectively. They also prohibited tsFT210 cells cell cycle progression at the G2/M phase (IC_50_s 197.5 and 14.0 μM, respectively) in the flow cytometry. It was assumed that the methoxy group in **42** decreased its inhibitory activity than that of **45** [60, 78]. From the *Edgeworthia chrysantha*-harboring *A. fumigatus* KR-019681culture EtOAc extract, **42** and **46** were separated that displayed notable antimicrobial potential versus *E. coli*, *S. aureus*, and *C. albicans* in the broth microdilution method with MICs 0.39 μg/ml [62].

New prenylated indole-diketopiperazine alkaloids: **6, 7, 38, 43**, and **47**–**49**, in addition to **5, 3, 8, 10, 21, 24, 27, 37**, and **42** were obtained from *A. fumigatus* Fres. isolated from holothurian *Stichopus japonicus* using Sephadex LH-20/SiO_2_ CC/preparative HPLC [32]. Compounds **43** and **47**–**49** feature spiro-oxindole diketopiperazines. Whilst **43** and **47** have an oxindole-diketopiperazine core as **42** with difference in the presence of 9-OH/8-OH in **43** and 8-OH/9-OH/N-attached isoprenyl unit in **47** with 2S/8S/9R/12S/18S configuration. Compounds **7, 38**, and **47**–**49** revealed cytotoxic potential against T lymphoblast (MOLT-4), (promyelocytic leukemia (HL-60), A-549, and BEL-7402 cells (IC_50_ 125.3-1.900 μM), whereas **6**, **7**, and **49** displayed potent activity versus MOLT-4, HL-60, and A-549 than **43, 47**, and **48**, compared to VP16 (IC_50_ 1.400–0.003 μM). Interestingly, **49** (IC_50_s 3.1, 2.3, and 3.1 μM, respectively) demonstrated the highest potential towards MOLT-4, HL-60, and A-549, while **6, 7**, and **38** were potent against HL-60 (IC_50_s 3.4, 5.4, and 1.9 μM, respectively) [32]. The new metabolites were assumed to originate from L-proline, L-tryptophan, L-methionine, and one or more isoprene moieties derived from mevalonate [32]. Additionally, from the EtOAc extract of *Erythrophloeum fordii*-associated *A. fumigatus*, **50**, along with **43** and **46** were identified [64] that had no effect on LPS (lipopolysaccharide)-induced nitric oxide (NO) production in mouse macrophages [64] (Fig. S5).

**Indole-quinazoline alkaloids.** Fumiquinazolines are hybrid alkaloids having an indole moiety linked to a pyrazino[2, 1-b] quinazoline-3,6-dione core that possess a wide range of bioactivities, including antiviral, anticancer, and antibacterial properties [65-76] (Tables S1 and S13). *Aspergillus* species are common producers of these metabolites.

**Fig. 3:**
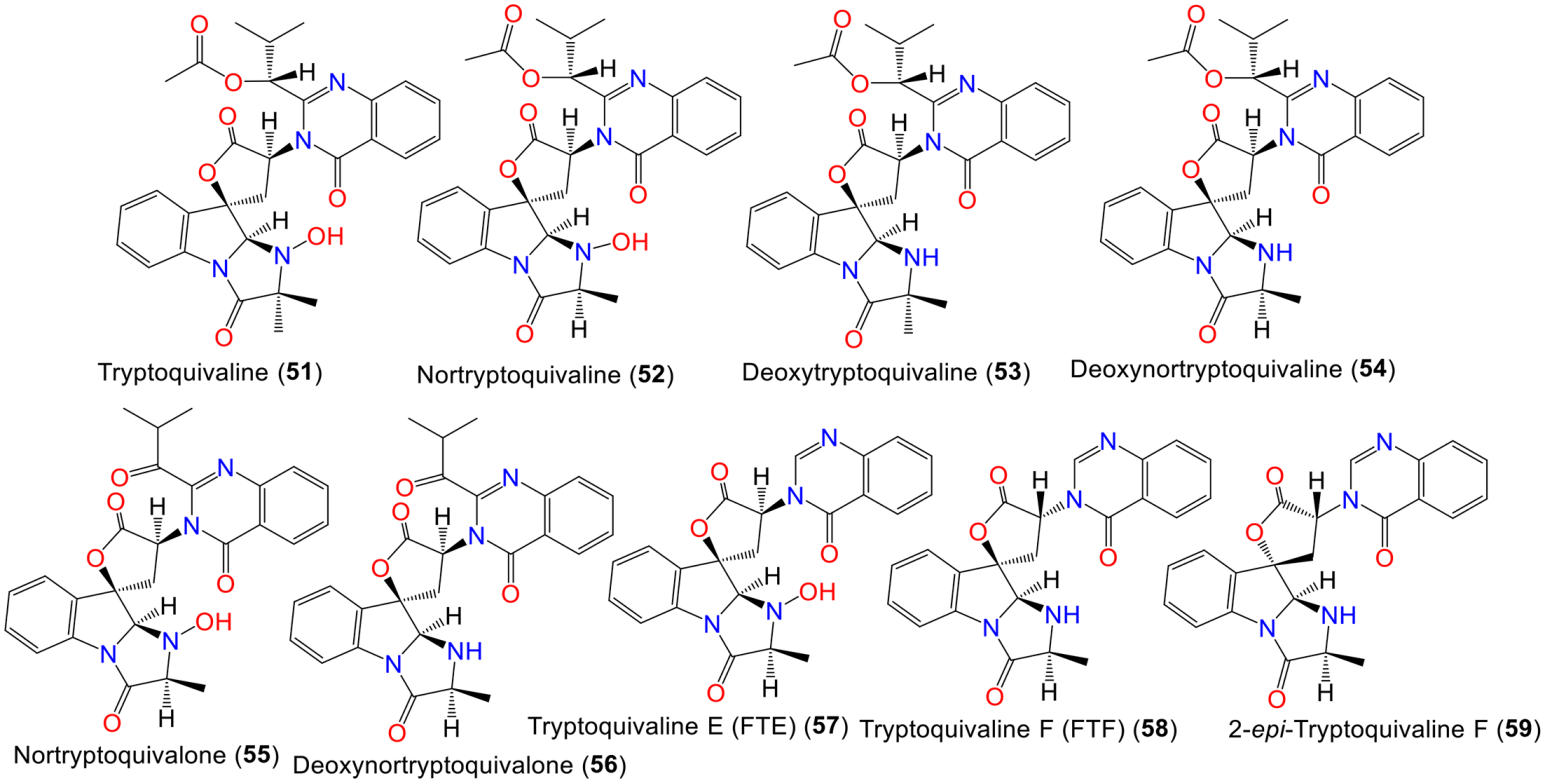
Examples of indole-quinazoline alkaloids (51–59) reported from *A. fumigatus*.

Compounds **51, 52, 57, 58**, and **60**–**63** were identified by Yamazaki *et al*. in 1979. These compounds were presumed to be derived from leucine, alanine, tryptophan, and anthranilic acid [28, 66] (Fig. 3). In another study by the same group, **64**–**66** were identified as structural isomers of **60, 52**, and **62**, respectively, based on the spectral and optical rotation data [67] (Fig. S6). Compounds **68**, **70**, and **71** were isolated from the mycelium of *A. fumigatus* obtained from saltwater fish *Pseudolabrus japonicus* gastrointestinal tract that were elucidated by spectral/chemical/X-ray analyses. These compounds were moderately cytotoxic against lymphocytic leukemia (P-388) cells [69]. The marine isolate *A. fumigatus* KMM-4631 yielded **71** and **73** that were elucidated by nuclear magnetic resonance (NMR)/mass spectrometry (MS) spectral tools [31].

In 2021, Yan *et al*. reported a new metabolite, **72**, together with its analog **71** from sea sediment-derived SD-406 strain [29]. Compound **72** has 3R/14R/17S/18S/20S and optical rotation -193.7, which was closely similar to **71**, except that 29-doublet methyl in **71** was substituted by an oxymethylene in **72**. Compound **72** exhibited weak activity versus *F. oxysporum* (MIC 64 μg/ml, respectively), while **71** was inactive. The results showed that C-29 hydroxylation diminished activity [29] (Fig. S7).

Tuan *et al*. stated that **71** and **73** revealed remarkable antibacterial potential versus *E. faecalis* (MICs 32.0 and 32.0 μg/ml, respectively).Besides **71** had also significant activity on *C. albicans* (MIC 64.0 μg/ml), however, **73** and **76** displayed weak potential on *C. albicans* (MICs 128.0 μg/ml). Additionally, **76** possessed marked cytotoxic efficacy on colon cancer (HT-29) and hepatocarcinoma (Huh7) cell lines (IC_50_s 9.7 and 10.3 μM, respectively), while **71** and **73** were weakly active (IC_50_s 60.9–70.9 μM). Further, only **71** displayed α-glucosidase inhibition capacity (% 13.6; Conc. 100 μg/ml), compared to acarbose (% 88.4 %) [54].

Shaaban *et al*. revealed that **73** and **74** possessed antimicrobial properties versus *B. subtilis*, *S. aureus*, *C. albicans*, and *M. miehi* (inhibition zone diameters 12/5, 12/15, 11/11, and 12/3 mm, respectively) in the disc-agar method [71]. Compound **76** possessed marked activity towards *B. subtilis*, *S. aureus*, *E. coli*, and *S. typhimurium* (MICs 1–4 μg/ml), compared to gentamicin (MICs 0.5-1.0 μg/ml) [36].

Additionally, **68**, **69**, **74**, and **75** (MICs 12.5–25 μg/ml) revealed notable antifungal potential, compared to **73** (MICs 25–50.0 μg/ml) versus *B. cinerea*, *A. solani*, *A. alternata*, *C. gloeosporioides*, *F. solani*, *F. oxysporum* f. sp. *niveum*, *F. oxysporum* f. sp. *vasinfectum*, and *G. saubinettii*, suggesting the significance of C-3 and N-22’s C-N bridge in activity [34].

A new alkaloid, **77**, with **58** were isolated from *A. fumigatus* KMM 4631 associated with *Sinularia* sp. soft coral using SiO_2_CC/HPLC. Compound **77** features quinazoline moiety and showed inhibitory activity against *S. aureus* ATCC-21027, *B. cereus* ATCC-10702, *E. coli* ATCC-15034, *P. aeruginosa* ATCC-27853, and *C. albicans* KMM-453 in the agar diffusion assay [61].

New pyrazinoquinazoline-indole glucosides: **79**–**81** were separated from *A. fumigatus* obtained from *Nemopilema nomurai* jellyfish using SiO_2_ CC/HPLC. Their structures were elucidated by spectral/time-dependent density-functional theory (TDDFT) calculations/electronic circular dichroism (ECD) methods (Fig. S8) [74].

These metabolites are examples of fumiquinzoline-type alkaloid glycosides that feature spiroquinazoline moiety with 3R/14R/17S/18S/20S, 3R/14R/17S/18S/20S, and 3S/14R/17S/18S/20S configurations, respectively. Compound **79** is similar to demethyl fumiquinazoline E, except **79** has 3-OH instead of 3-OCH_3_ in demethyl fumiquinazoline E. Whilst **80** has 3-H instead of 3-OH in **79**, and **81** is a C-3 stereoisomer of **80**. They showed no antibacterial or cytotoxic capacities against a panel of strains and cancer cell lines [74]. They were proposed to be precursors for fumiquinazolines before hydrolysis [74]. They possess indole and pyrazino[2,1-b]quinazoline-3,6-dione moieties. Compounds **79**–**81** were assumed to be derived from tryptophan, anthranilic acid, and alanine. Compounds **79** and **80** are formed upon the introduction of L-alanine (Scheme S1) [74].

The new alkaloids: **82** and **83**, together with **71** and **75** were isolated from the EtOAc extract of deep-sea-derived *A. fumigatus* SCSIO-41012. Compound **82** has quinazoline-indole skeleton that is closely related to **76** with the existence of C-3 oxygenated methine in **82** instead of the CH_3_ group in **76**. Its 3R/14R configuration was assigned using time-dependent density-functional-theory electronic circular dichroism (TDDFT-ECD) calculations. On the other hand, **83** possesses quinazolin-4(3*H*)-one moiety and *gem*-methyl imidazoindolone core with 1,2-disubstituted benzene rings similar to tryptoquivaline F. Compound **82** showed noticeable antifungal potential (MIC 1.56 μg/ml) against *F. oxysporum* f. sp. *momordicae*, however, **83** was significantly active against *S. aureus* 16,339, *S. aureus* 29,213, and *A. baumanii* ATCC-19606 (MICs1.56, 0.78, and 6.25 μg/ml, respectively) [70]. Additionally, **84** and **85**, new indole-quinazoline alkaloids glucosides were isolated from the SAI12 strain associated with *Sonneratia apetala* mangrove plant and elucidated by spectral/ECD analyses. Unfortunately, these compounds showed no antibacterial and cytotoxic capacities in the two-fold serial dilution method and MTT assays, respectively [73]. The extract of *A. fumigatus* MF029 associated with *Hymeniacidon perleve* sponge demonstrated moderate effectiveness versus *M. bovis* *bacillus* Calmette–Guerin (MIC 12.5 μg/ml). The new alkaloid, **89**, was identified along with its related analog **88**. In compound **89**, the C-12 methyl in **88** was replaced by oxygenated methylene, suggesting the alanine moiety in **88** was changed to serine in **89**. Unfortunately, these compounds exhibited no activity against different bacterial strains [75]. Compound **90** was isolated as a new metabolite from *Heteroscyphus tener* liverwort-associated *A. fumigatus*. This compound is related to **88** with 2S/3S/11S/14S and -110.9 optical rotation value [45]. Interestingly, **91** displayed inhibitory capacity versus sugarcane-associated *Fusarium* sp. (Conc. 100 μM; inhibitory rate 77%) [51] (Fig. S9).

**Diketopiperazine alkaloids.** From *A. fumigatus*, a number of indole-diketopiperazine alkaloids with cell cycle inhibitory action were reported that were biosynthetically derived from amino acids such as L-tryptophan and L-proline [32, 34, 35, 37, 42, 43, 45, 48, 54, 58, 63, 79-81] (Table S2). It is noteworthy, compound **93** revealed a potent plant growth inhibitory effectiveness than glyphosate. It also prohibited *Raphanus sativus* (turnip) root and shoot elongation (RIs (response index) -0.70 and -0.76 and, respectively, Conc. 120 ppm) and strongly prohibited (RI -0.9; Conc. 40 ppm) *Amaranthus mangostanus* (amaranth) seedling growth, therefore, it could be developed a natural eco-friendly herbicide [37].

**Fig. 4:**
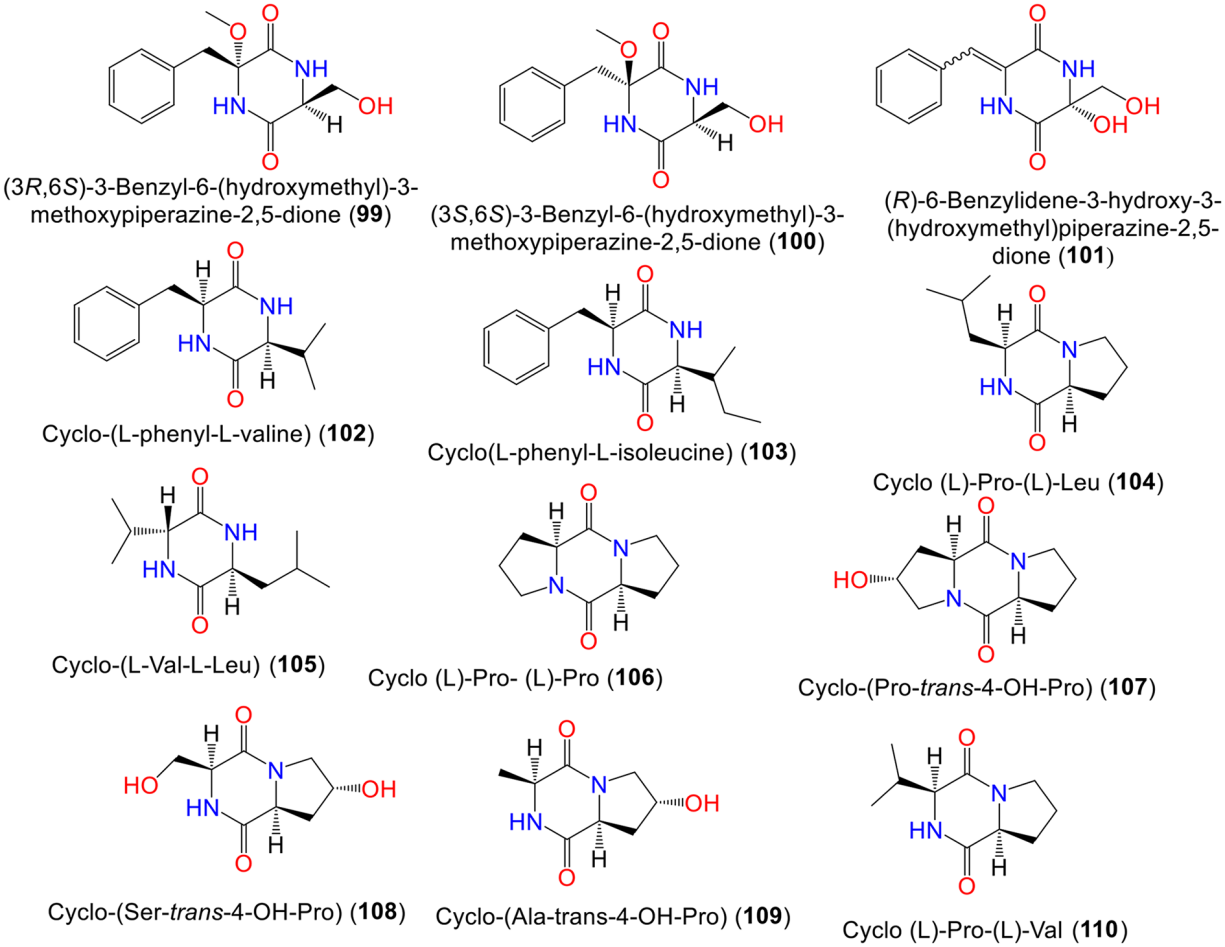
Examples of diketopiperazine alkaloids (99–110) reported from *A. fumigatus*.

In another study on the same stain, new dioxopiperazines; **99**, **100**, and **101** were isolated by Zhao *et al*. Compounds **99** and **100** are epimers, belonging to dioxopiperazine analogs with a benzyl group that differ in stereo-configuration at C-6. Compound **101** is structurally similar to **99** except for the presence of a C_6_-C_7_ double bond and the absence of C-6 methoxy [79]. The new diketopiperazine, **105** that is structurally similar to cyclo-(Val-Val) with an additional methylene group was obtained from *Erythrophleum fordii*-harboring *A. fumigatus* (Fig. 4). This compound exhibited nonsignificant cytotoxic activities (IC_50_ > 10 μM) against HCT-8, Bel-7402, BGC-823, A-549, and A2780 cell lines in the MTT assay [80].

The diketopiperazines **95, 97, 104, 106, 110, 111**, and **116** were reported from soil-associated *A. fumigatus* culture broth collected from Pantanal/Brazil that were assigned using NMR/Marfey's method. These compounds prohibited the growth of *M. luteus* and *S. aureus* (MIC 2.9 μM/l), in comparison to penicillin (MIC 0.12 and 0.25 μM/l, respectively) [81] (Table S2). Whilst **93, 97, 98, 102,103, 111, 115**, and **116** separated from EtOAc extract of *Scutellaria formosana* had no antimicrobial, cytotoxic, or anti-inflammatory properties [63] (Fig. S10).

**Fig. 5:**
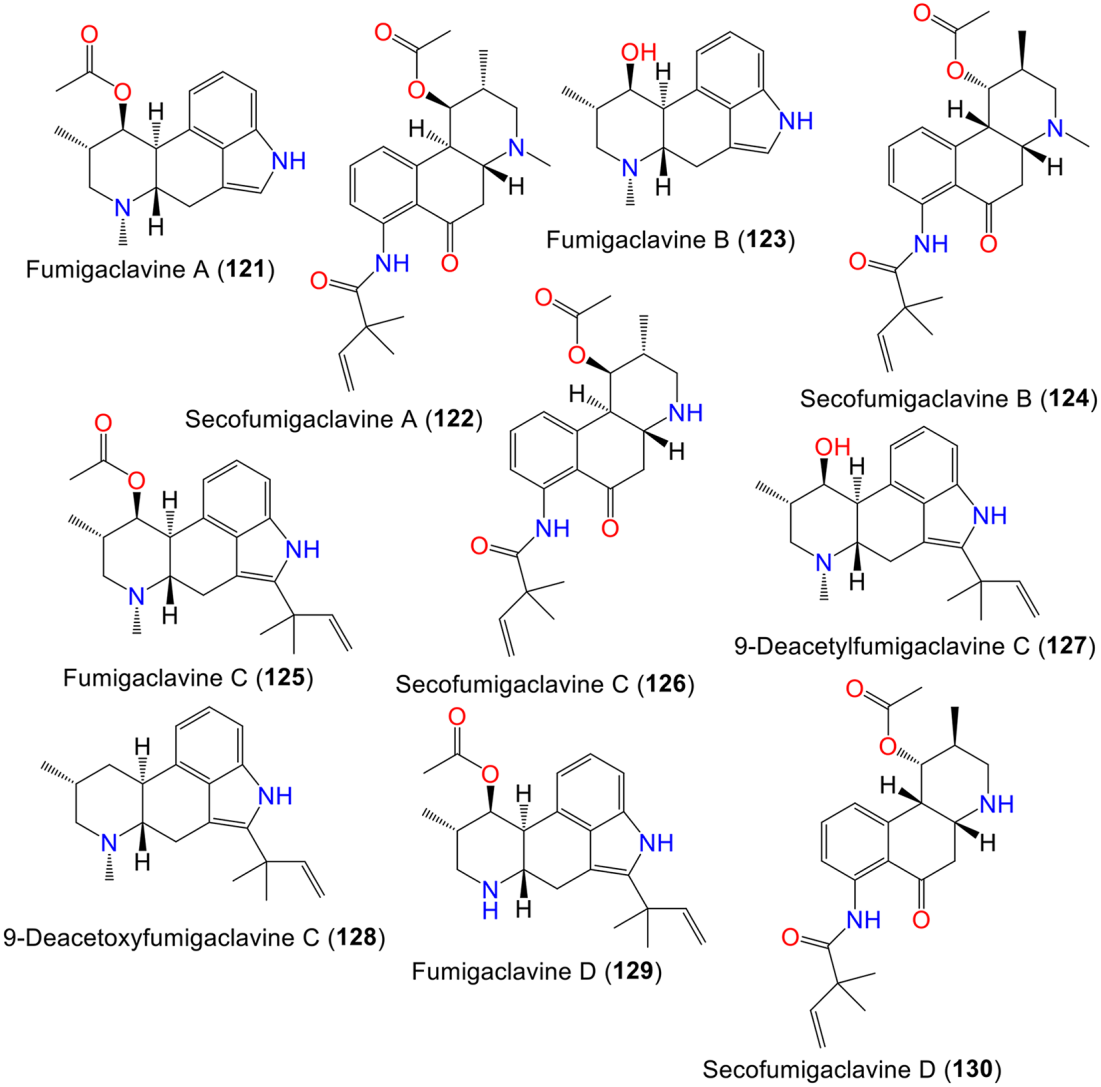
Chemical structures of clavine-type ergot alkaloids (121–130) reported from *A. fumigatus*.

**Clavine-type ergot alkaloids.** Different studies reported the separation of clavine-type ergot alkaloids [34, 39, 42, 44-46, 62, 72, 82-86] (Table S3). *A. fumigatus* isolates Bio-guided fractionation of the chloroform extract of *A. fumigatus* isolates, which contained toxic chemicals that caused prolonged shaking, convulsions, and/or death in day-old cockerels, led to the isolation of compounds **121, 123**, and **125**. In day-old cocks, the LD_50_ of **125** was approximately 150 mg/kg oral dosage. Additionally, *A. fumigatus* extracts developed irritability, severe diarrhea, and appetite loss in calves with serous enteritis and interstitial alterations [59]. Lui *et al*. reported that **121** (Conc. 20.0 μM) possessed immunomodulatory capacity by promoting the proliferation of splenic lymphocytes induced by LPS and concanavalin A (Con-A), with a proliferation rate of 39.4% [50]. Additionally, Zhang *et al*. reported new alkaloids **122**, **124**, **126**, **130**, and **132**, along with related analogs, **125** and **129** from EtOAc extract of intestinal human-derived *A. fumigatus* CY018. These compounds are characterized by a rare 2,3-seco-indole core skeleton, which was synthesized under photochemical conditions catalyzed at 365 nm by riboflavin from the related alkaloids; **125** and **129** [82] (Fig. 5).

Compound **125**, isolated from *A. fumigatus* associated with marine green algae collected from Seosaeng-myeon/Ulsan/Republic of Korea (Table S3), showed marked cytotoxic capacity towards breast adenocarcinoma (MCF-7) cells in the MTT assay. It induced apoptosis in MCF-7 *in vitro* through NF-κB and Akt/PI3 signaling, resulting in the activation of the mitochondrial cell death pathway [83].

A study by Zhang *et al*. revealed that compounds **124**, **130**, and **132** displayed marked inhibitory capacities against NO production induced by LPS (IC_50_s 3.48, 7.83, and 9.47 μM, respectively), while **122**, **125**, **126**, and **129** had moderate effects (IC_50_s 60.37 to 22.77 μM) [82]. Compound **124** demonstrated a noteworthy anti-inflammatory impact through its binding to MD2 (Myeloid differentiation factor-2), which deactivated the TLR4/MD2 signaling pathway. In the molecular dynamic (MD) simulation, Tyr131, an amino acid residue, was found to have a crucial role in the interaction of **124** with MD2, suggesting its potential for development as MD2 inhibitor[82]. Furthermore, compound **125** was reported to have better immunomodulating capacity via promoting the proliferation of splenic lymphocyte cell induced by LPS and Con-A (proliferation rates 38.1 and 21.3%, respectively; Conc. 20.0 μM) than the positive control [50]. Compounds **127** and **128** are new alkaloids isolated from *A. fumigatus* by SiO_2_/Sephadex LH-20 CC/HPLC. These compounds are similar to **125**, except for the absence of acetyl and O-acetyl groups at C-9, respectively. Compounds **127** and **128** displayed apparent selective inhibition of K562 cells (IC_50_s 41.0 and 3.1 μM, respectively), compared to doxorubicin hydrochloride (IC_50_ 1.2 μM) [52]. A study by Wang *et al*. on the VDL36 strain isolated from *Vaccinium dunalianum* leaf collected in Wuding County/Yunnan Province/China resulted in the separation and characterization of novel alkaloids: **134** and **135**, in addition to **125** using D101 macroporous resin/SiO_2_CC/RP-18 CC/HPLC and spectral/ECD analyses [55] (Fig. S11).

Compound **134** with 3S/5R/8R/9S/10R is proposed to be formed from **125** through the pinacol rearrangement that potentially plays an important role in γ-lactam moiety formation in ring A (Scheme S2). Compound **135** has the same configuration as **125** (5R/8R/9S/10R), however, it possesses N-OCH_3_ instead of N-CH_3_ in **125** [55]. These compounds were assessed for antifungal activity against phytopathogenic fungi: *F. oxysporum*, *C. versicolor*, *F. solani*, *B. cinerea*, and *F. graminearum* using the broth microdilution method. Interestingly, compound **134** (MICs 15.62–62.5 μg/ml) demonstrated antifungal capacities compared to ketoconazole and carbendazim (MICs 7.8–31.25 and 1.95–7.8 μg/ml, respectively). Furthermore, compound **134** was found to display *in vivo* protective and curative efficacy against *B. cinerea* (tomato gray mold causative pathogen) [55].

From the marine-derived *A. fumigatus* ZTF001 EtOAc extract, Zhang *et al*. reported the separation of new alkaloids, **136** and **137**, along with **138**–**140** using SiO_2_ CC/prep TLC/HPLC [86]. Compound **136** is a 2-(1,3-dimethylprop-1-ene) analog of **138**, the configuration of the new compounds was assigned as 5R/8R/10S and 5R/ 8S/10S, respectively based on spectral/CD analyses. In the MTT assay, compounds **136**–**138** and **140** had weak cytotoxic potential versus P388 (mouse leukemia) cell line (IC_50_s 64.9, 259.7, 333.3, and 218.8 μM, respectively), whereas **139** was inactive [86].

**Spiro-heterocyclic γ-lactam analogs.** Spiro-γ-lactams, also known as spiropyrrolidin-2-ones, are a group of spirocyclic molecules that were isolated from *A. fumigatus* [29, 34, 35, 39, 44-46, 48, 49, 51-53, 55, 58, 62, 64, 85-95](Table S3).

Abdulwahed *et al*. reported that compound **141** decreased the secretion of PCKS9 (Proprotein convertase subtilisin/kexin type-9) with an IC_50_ of 1.20 μM and inhibited its interaction with LDLR (LDL receptor) at concentrations 10–150 μM (Table S13). This resulted in lowering the circulating cholesterol and slowing the growth of hormone-dependent BT-474 breast cancers in a nude mice xenograft model [96]. Moreover, **141** simultaneously decreased the breast tumor recurrence marker CA 15-3 (cancer antigen 15-3) and serum levels of 17β-estradiol in mice. Following surgical excision of the primary tumor, it demonstrated the potential to suppress the locoregional recurrence of BT-474 breast carcinoma cells. It effectively suppressed breast cancer recurrence, induced optimal systemic hypocholesterolemia, and inhibited tumor development *in vivo*. Notably, **141** functioned both intracellularly and extracellularly, whereas existing FDA-approved monoclonal antibody (mAb) medications act only extracellularly. Thus, **141** represents a unique first-in-class proprotein convertase subtilisin/kexin type-9 (PCSK9)-targeting lead that can be used to manage the progression and recurrence of hormone-dependent breast cancer [96].

The new metabolite **142**, along with **141**, were reported from the HA 57-88 strain and characterized by spectral tools. These compounds displayed inhibitory capacity on membrane-bound and solubilized chitin synthase (IC_50_s 192 and 81 μM, respectively), however, they had no antimicrobial potential due to the lack of epoxy ring [87].

Compound **143**, in addition to **141** and **145**, were characterized from SD-406 strain isolated from sea sediment using spectral/chiral HPLC/X-ray/J-based configuration analyses, as well as quantum chemical calculations [29]. Compound **143** is similar to **141** (configuration 5S/8S/9R/10S/11S; optical rotation -5), but it possesses a different 5S/8S/9R/10R/11S configuration and optical rotation value of +6.0 [29]. Compound **143** (MIC 16 μg/mL) with the side chain extension revealed moderate potential versus *F. graminearum* plant pathogen, however, **141** and **145** had no activity [29].

**Fig. 6:**
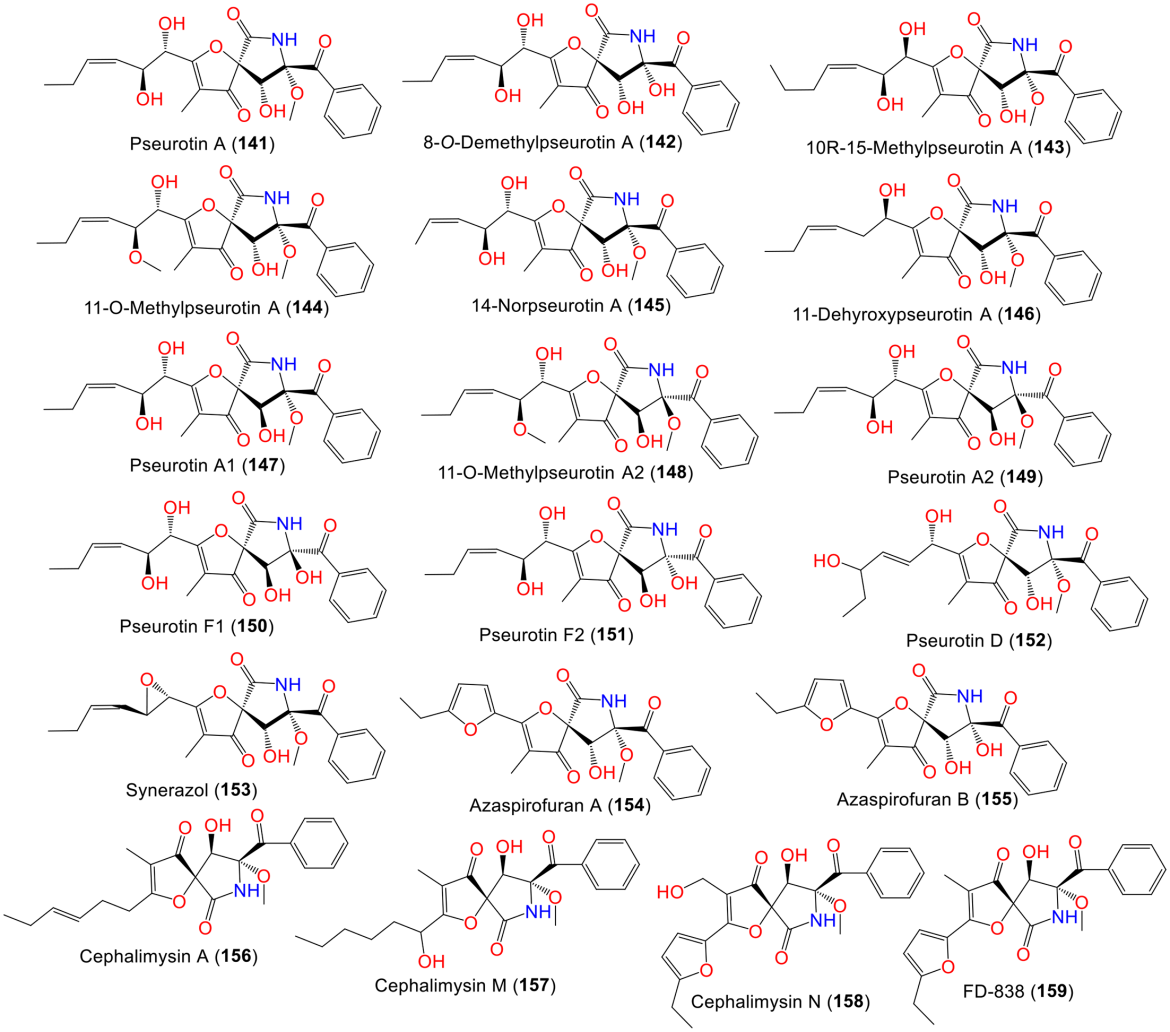
Examples of spiro-heterocyclic γ-lactam analogs (141–159) reported from *A. fumigatus*.

Boot *et al*. separated **144**, a new compound from sea sediment-derived 030402d strain, along with **141** using SiO_2_ CC/HPLC that were assigned by spectral/optical rotation/J-based analyses (Fig. 6). Compound **144** possessed antifungal potential versus cell cycle-related mutant and wild-type *S. cerevisiae* (IZDs (inhibition zone diameters) 9 and 4 mm, respectively), while **141** was inactive against both strains [88]. Also, **145** remarkably induced PC12 cells neurite outgrowth (Conc. 10.0 μM) more than **141** [52]. Shi *et al*. reported that **141** had anti-inflammatory effectiveness through suppression of LPS-caused NO formation in mouse macrophages (IC_50_ 5.20 μM), in comparison to dexamethasone (IC_50_ 2.5 × 10^-2^ μM) [64].

A novel alkaloid, **146**, was reported by Wang *et al*. from the VDL36 strain isolated from *Vaccinium dunalianum* leaf. This compound is an analog of **141**, having an 1-oxa-7-azaspiro[4.4]non-2-ene- 4,6-dione framework and 5S/8S/9R/10R configuration [55].

Co-culture of *A. fumigatus* MBC F1-10 with *Streptomyces bullii* resulted in the production of a new metabolite, **148**, along with **144** [35]. Compound **148** is a 9-epimer of **144**. It was found that **148** displayed anti-leishmanicidal potential versus *L. donovani* (EC_50_ 18.4 μM) with cytotoxic effect against normal human lung fibroblasts (MRC5) cells, while **144** was inactive [35].

Compound **153**, a new antifungal antibiotic that is related to **141**, was reported from soil-accompanied SANK-10588 strain collected from Prachuap khiri khan/Thailand. Compound **153** had activity against *C. albicans* and possessed marked synergistic action with fluconazole and clotrimazole [93], whereas **141** was found to possess selective antibacterial potential (MIC 64 μg/ml) versus *Shigella shigahe* and *B. cereus* in the serial dilution method [90].

Yamada *et al*. reported the isolation of **156** from the *Mugil cephalus* marine fish-associated *A. fumigatus* that was characterized by NMR/chemical/modified Mosher’s methods. This compound demonstrated marked cytotoxic potential versus HL-60 and P388 cell lines (IC_50_ 9.5 and 15.0 nM, respectively) in the MTT assay [94].

Copmans *et al*. assessed the antiseizure potential of the heterospirocyclic γ-lactams, **144**, **149**, **150**, **144**, **152**, **154**, and **155** separated from Red Sea sediment-derived *A. fumigatus* collected from Hurghada/Egypt using larval zebrafish model [91]. It is noteworthy that compounds **149** and **154** were proved as antiseizure agents, while other analogs were inactive. Also, **149** and **154** ameliorated PTZ (pentylenetetrazole)-induced epileptiform discharges, as well as lowered seizure duration in mouse 6 Hz (44 mA) psychomotor seizure model dose-dependently. Therefore, **149** and **154** were proposed as lead compounds for treating epileptic seizures [91]. From the EtOAc (ethyl acetate)/MeOH (methanol) extract of sea-sediment derived *A. fumigatus* CUGBMF-170049 collected from the Bohai Sea/China, new spiro-heterocyclic γ-lactam analogs, **157** and **158**, along with **141**, and **159** were separated by Xu *et al*. Compound **157** is similar to **141** with an oxygenated side chain, instead of the unsaturated oxygenated chain in **141**, while **158** is related to **159**, in which the C-3 methyl in **159** is replaced by oxygenated methylene in **158**. These compounds had no activity against *S. aureus* ATCC-6538 and MRSA ATCC-29213 [92].

**Indole and indoline alkaloids.** The reported indole and indoline alkaloids from this fungus were listed in Table S4 [39, 47, 54, 64, 97, 98] and discussed below.

Compounds **160** and **161** that possess a 10-(indol-3-yl) ring and a perhydroisoindolone moiety were reported by Flewelling *et al*. from *A. fumigatus* AF3-093A associated with *Fucus vesiculosus* brown alga. These compounds demonstrated growth inhibition capacities on *Mycobacterium tuberculosis* H37Ra and methicillin-resistant *S. aureus*in the microplate resazurin and microbroth dilution assays, respectively [97].

*A. fumigatus* H22 EtOAc extract showed potent anti-MRSA capability. The investigation of this extract resulted in the separation of new constituents, compounds **162**–**164**, that were characterized by spectral/ECD analyses, as well as chemical derivatization and ECD/quantum NMR calculations [39]. Compound **162** and **163/164** feature indole pyridine carboxylic acid core with GABA and proline moieties respectively [39] (Fig. S12).

Mai *et al*. identified new alkaloids: **166**–**172** and previously reported **165, 173, 175**–**178**, and **180** from *A. fumigatus* 0338 associated with *Nicotiana tabacum* (Fig. S13). Compounds **166**–**168** are rare linear prenylated fused 6/6/5 tricyclic indole alkaloids, whereas **169** and **170** have rare C-5 isopentenyl substituents and **171** and **172** are new 6-methyl-1,7-dihydro-2*H*-azepin-2-one-bearing indole alkaloids [98]. Compounds **172** and **173** demonstrated noticeable anti-*G. cichoracearum* properties (%inhibition 82.6 and 85.2%, respectively; Conc. 250 μg/ml), compared to carbendazim (78.6%). Compound **173** could directly target and destroy *G. cichoracearum* spores, and also restore leaf tissue characteristics to enhance plant resistance to pathogens. Compounds **172** and **173** activated several defense-related enzymes and increased the expression of defense-related genes in tobacco leaves infected with powdery mildew. It was proved that the indole core in these compounds was the source of their anti-*G. cichoracearum* action, as indicated by molecular docking data. Additionally, the methoxy substitution in compound **172** enhanced its activity. These indole alkaloids could be new anti-powdery mildew candidates for pesticide development [98].

From sea cucumber (*Colochirus quadrangularis*)-associated *A. fumigatus* M580, a new indole glucoside, **181** was characterized by Tuan *et al*. Compound **181** is like indole-3-carboxylic acid *O*-β-D-glucopyranosyl ester with an additional methoxy group at C-6 of the indole moiety [54].

**Amide-containing alkaloids.** Amides are among the metabolites biosynthesized by *A. fumigatus* (Table S4)[29, 71, 75, 79, 99-104].

The mixed culture of *Streptomyces peucetius* and *A. fumigatus* produced new sulfated formyl xanthocillin analogs: **183** and **185**, along with **182** and **184** that were separated using SiO_2_ CC/HPLC. Compound **185** has 1,4-diphenylbuta-1,3-diene-2,3-diyl-diformamide with sulfate and methoxy groups. Compound **183** possessed notable activity versus different cell lines (IC_50_s 1.12–0.65 μM), while **184** was moderately active (IC_50_s 2.13–1.37 μM) [99] (Fig. S14).

A study by Gill *et al*. stated that *A. fumigatus* derived from soil produced a high yield of **186** (~117 mg/l). This compound demonstrated mycelial inhibition only against *P. ostreatus*, *G. lucidum*, and *A. oryzae* (% inhibition 80, 20, and 14%, respectively) [100]. Compound **187** (IC_50_ 17.4 μM) exhibited moderate activity against K562 cells using the sulphorhodamine B (SRB) method [79]. Compound **189**, an amino acid-pyrone derivative was isolated from the KARSV04 strain, which demonstrated a cytotoxic effect on MCF-7 cells (IC_50_ 70.57 μg/ml). This effect was synergized in co-treatment with doxorubicin through the accumulation of doxorubicin-treated MCF-7 cells in the G2/M phase [102]. Additionally, 189 inhibited the growth of ductal breast epithelial (T47D) cells and modulated the S-phase in the cell cycle, indicating different mechanisms in regulating cell cycle progression in the two cell lines [102].

Song *et al*. identified a new dihydrofuranone derivative: **191**, together with **190** from *A. fumigatus* MF029. Compound **191** was proposed to be a cyclized esterification product of **190** [75] (Fig. S15).

Compound **192** is a new five-membered lactone-containing compound, which is a dehydrated analog to sphingofungin H that was formerly isolated from *A. penicilliodes* with an additional C2-C3 double bond. It demonstrated notable antibacterial capacity versus *V. alginolyticus*, *E. tarda*, and *P. aeruginosa* (MICs 8 μg/ml), compared to chloramphenicol (MICs 1–4 μg/ml) [29]. Yu *et al*. reported that *A. fumigatus* D/*F. oxysporum* R1 co-culture yielded a new amide, **194**. Its 4S configuration was established by ECD and optical rotation (+11.7) measurements [101]. Kalyani *et al*. identified **195** from *A. fumigatus* MF-1 fraction that possessed a cytotoxic effect towards cervical epitheloid carcinoma (HeLa) cell lines (IC_50_ 74.38 μg/ml, Conc. 200 μg/ml) [104].

**Thiodiketopiperazine and other alkaloids.** Thiodiketopiperazines alkaloids were identified from different *Aspergillus* species, including *A. fumigatus* that are distinguished by a sulfur-bridged six-membered diketopiperazine unit (Table S4) [31, 33-35, 39, 52-54, 62, 71, 72, 84, 105-108]. In addition, other minor alkaloids belonging to different classes were also listed in Table S4 [34, 42, 47, 48, 64, 70, 71, 89, 99, 109-111].

**Fig. 7:**
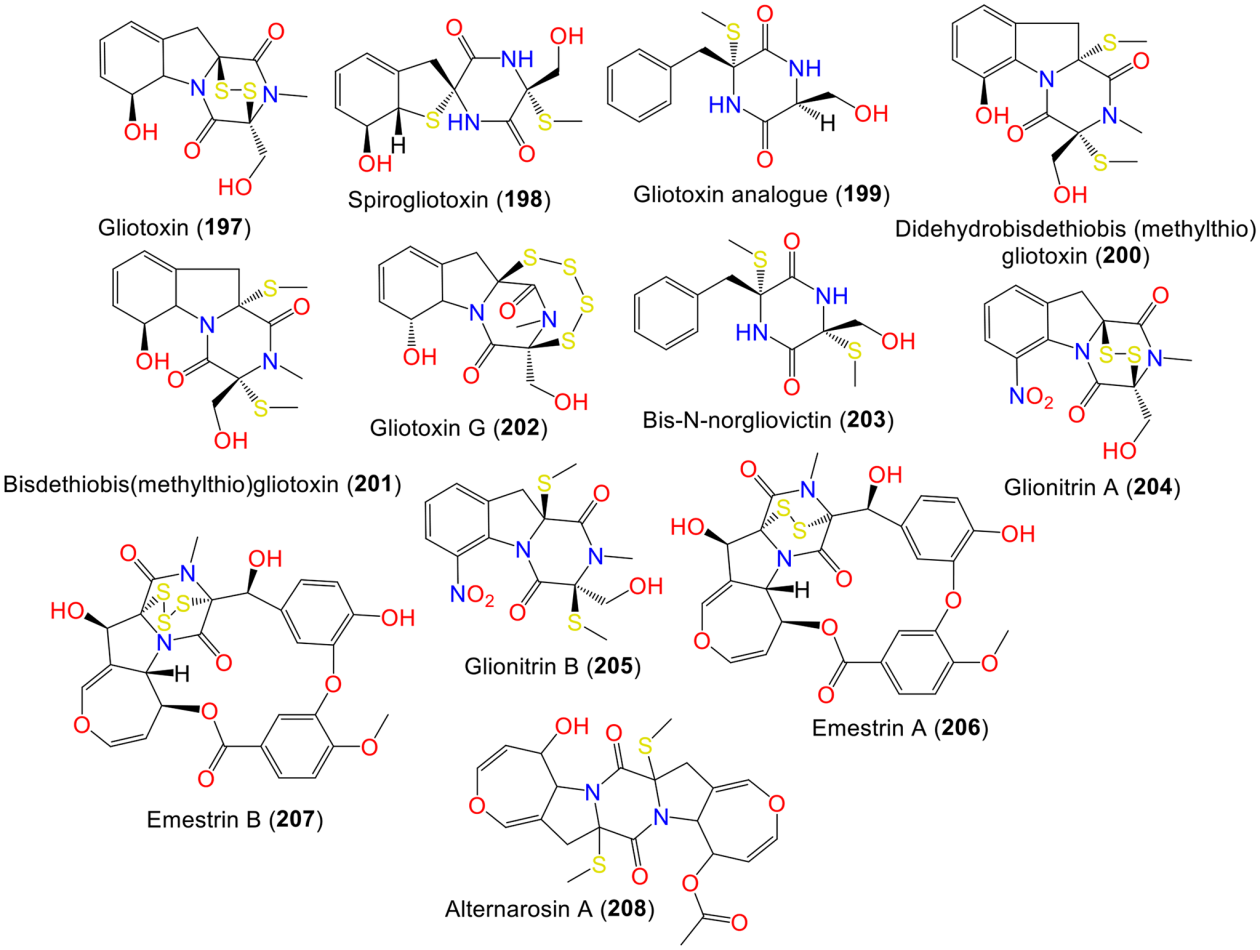
Examples of thiodiketopiperazine (197–208) alkaloids reported from *A. fumigatus*.

Gliotoxin **197** is a fungal metabolite that belongs to the epipolythiodioxopiperazine family with bridging disulfide that is derived from serine and phenylalanine with a variety of bioactivities, such as antiviral and antibacterial capabilities [105]. It was reported to induce apoptotic cell death in a variety of cell types, including fibroblasts, macrophages, thymocytes, and splenocytes [112]. According to Vigushin *et al*. **197** was verified as a dual inhibitor of geranylgeranyltransferase I and famesyltransferase with strong antitumor efficacy against breast cancer both *in vitro* and *in vivo* [113]. Compounds **197** and **201** were separated from the *Edgeworthia chrysantha*-harboring *A. fumigatus* KR-019681culture EtOAc extract (Fig. 7). These compounds exhibited marked antimicrobial properties versus *E. coli*, *S. aureus*, and *C. albicans* in the broth microdilution method, whereas **201** showed powerful inhibitory effectiveness towards *C. albicans*, *S. aureus*, and *E. coli*, respectively (MICs 0.39 μg/ml) [62].

Zhao *et al*. reported a new gliotoxin analog **199**, with **197**, **200**, **201**, and **203** from marine-sediment accompanied *A. fumigatus* Fres that were isolated by Sephadex LH-20 CC/HPLC and assigned based on spectral methods. Compound **199** has 1-substituted benzene, dioxopiperazine, and cyclo-(Phe-Ser) moieties. In the SRB assay against the tsFT210 cell line, **197** (IC_50_ 0.15 μg/ml) possessed substantial cytotoxic activity, however, **200** was weakly active (IC_50_ 89.8 μg/ml) and other metabolites had no activity [105]. Besides, **202**, a new tetrasulphide metabolite with an epidithiodioxopiperazine skeleton was separated from *A. fumigatus* F15 mycelia using SiO_2_ CC/PrepTLC [106].

The co-culture of *A. fumigatus* KMC-901 and *Sphingomonas* sp. KMK-001A derived from mine drainage yielded a new diketopiperazine disulfide, **204** that was characterized by NMR/CD/Xray. This compound is a 3S,10aS-diketopiperazine disulfide having a nitro aromatic ring that is related to dehydrogliotoxin and **197** [107]. Compound **204** had prominent antibacterial potential versus *S. aureus* (MIC 0.78 μg/ml) in the broth microdilution assay. It also displayed potent cytotoxic capacity on (colon cancer) HCT-116, A-549, gastric adenocarcinoma (AGS), and (prostate carcinoma) DU145 cell lines (IC_50_s 0.82, 0.55, 0.45, and 0.24 μM, respectively) and against MCF-7 and HepG2 (IC_50_s 2.0 and 2.3 μM, respectively) [107]. It is noteworthy that **204** was not detected in KMK-001 or KMC-901 monoculture broths, suggesting **204** originated through the interactions of *A. fumigatus* with *Sphingomonas* strain KMK-001. Thus, the microbial competition that takes place in coculture studies might lead to the synthesis of novel chemical compounds and potential therapeutic targets.

The coculture of *A. fumigatus* KMC- 901/*Sphingomonas* sp. KMK-001 isolated from acidic coal mine drainage yielded a new diketopiperazine, **205**, that was identified as (3S,10aS)-dithiomethylglionitrin A based on NMR/CD and semi-synthesis using **204**. Compound **204** demonstrated marked cytotoxic potential versus the DU145 cell line, whereas **205** had no effect on DU145 cell viability, whereas it diminished the DU145 cell invasion (Conc. 60 μM; 46% inhibition). Hence, **205** could be an antimetastatic agent in cancer therapy [108].

Rateb *et al*. stated that adding N-butyryl-DL-homoserine lactone to *A. fumigatus* MBC F1-10 /*Streptomyces bullii* culture medium led to the formation of **206** and **207** that were separated by SiO_2_/Sephadex LH-20 CC/HPLC and identified by spectral/CD/optical rotation measurement. They displayed no anti-trypanosomal and anti-leishmanial properties [35]. Liu *et al*. stated that **208** which was separated from the sea-sediment derived SZW01 strain, displayed weak α-glucosidase inhibition capacity, compared to acarbose [95].

Compound **209** possessing 1,4-benzodiazepine-2,5-dione moiety was isolated from the EtOAc extract of the deep-sea-derived *A. fumigatus* SCSIO-41012 (Fig. S16) [70]. A study by El-Sayed *et al*. on the *Catharanthus roseus*-associated *A. fumigatus* EtOAc extract using SiO_2_ CC/HPLC led to the isolation of **211** that was verified by HPLC/NMR/FTIR/LC-MS [109]. This metabolite belongs to epothilones which are macrolactones produced by *Sorangium cellulosum* myxobacterium and are known for their broad anticancer potential towards different tumors with greater affinity to stabilize microtubule arrays during cellular division. This compound (IC_50_s 10.21, 6.4, and 8.7 μM, respectively) demonstrated potent antiproliferation capacity towards LS174T, HepG-2, and MCF-7 cell lines [109].

In 2022, Xu *et al*. characterized new benzothiazoles: **212** and **213** from cave soil-derived *A. fumigatus* GZWMJZ-152. Their structures and configurations were elucidated by spectral/X-ray/ECD calculations [110]. These metabolites have benzothiazole core, in which the H-2 in **213** was replaced by oxymethylene group that was assigned as 4-methoxy-7-methylbenzo[d]thiazole-5,6-diol and 2-hydroxymethyl-4-methoxy-7-methylbenzo[d]thiazole-5,6- diol, respectively [110]. In the 1,1-Diphenyl-2-picrylhydrazyl (DPPH) assay, **212** and **213** scavenged DPPH radicals (IC_50_s 23.73 and 3.45 μM, respectively) [110].

*Cynodon dactylon*-accompanied *A. fumigatus* CY018 biosynthesized the tricyclic alkaloid **214**, which was separated from the EtOAc extract by SiO_2_CC. This compound inhibited *C. albicans* (MIC 75.0 μg/ml), compared to ketoconazole (MIC 31.5 μg/ml) [42]. A study by Mandal *et al*. aimed to optimize the production of the broad-spectrum antibacterial, compound **216** revealed that *A. fumigatus* nHF-01 produced the greatest quantity of 5-butyl-2-pyridine carboxylic acid when fed the SSF (solid-state fermentation) with lignocellulosic substratum as opposed to SmF (submerged fermentation). Also, SSF had lower energy requirements, higher volumetric productivity, and less affluent generation. Thus, **216** can be produced on an industrial scale by *A. fumigatus* nHF-01 using lignocellulosic sugarcane bagasse [111].

### Peptides

Cyclic pentapeptides, cyclic peptides typically made up of five amino acid residues were reported from this (Table S5) [114, 115]. Among these, **224**–**226** were reported from *A. petrakii*, *Hamigera ingelheimensis*, and *Hamigera avellanea*. Compound **224** raised blood pressure, **225** enhanced the effects of antitumor agents, and **226** showed inhibitory action on quorum sensing [114]. Wang *et al*. reported that the chemical investigation of the EtOAc extract of GXIMD-03099 strain, isolated from *Acanthus ilicifolius*, led to the separation and characterization of **226**–**229**, along with **224**, **225**, **230**, and **231** using spectral/X-ray analyses and Marfey’s method [114].

**Fig. 8:**
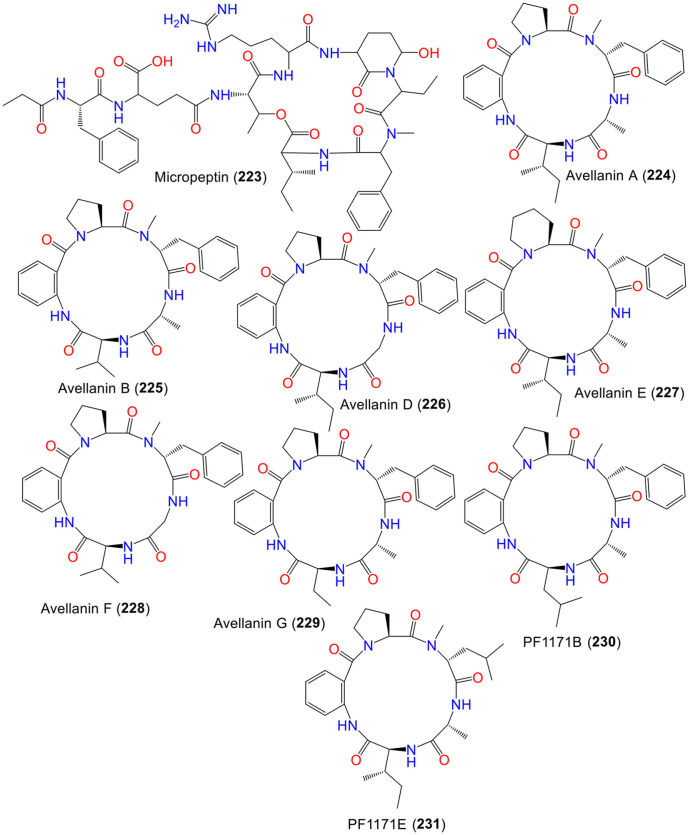
Chemical structures of peptides (223-231) reported from *A. fumigatus*.

Compounds **227** and **229** feature uncommon Pip and Abu amino acid residues, respectively (Fig. 8). All metabolites were examined for antibacterial and insecticidal activities. Compound **227** possessed insecticidal potential versus *Culex quinquefasciatus* newly hatched larvae (LC_50_ 86.6 μM), while **229** demonstrated weak antibacterial capacity against *Vibrio harveyi* (MIC 5.85 μM) in the microplate assay method [114], suggesting a crucial role of Pip and Abu for insecticidal and anti-*Vibrio* efficacy [114].

### Terpenoids

Pyripyropenes represent unique pentacyclic sesquiterpenoids, having pyridine and α-pyrone moieties, forming a steroid framework. These compounds represent the major type of terpenes reported from *A. fumigatus*. Some of them have been found to possess ACAT (Acyl-CoA: cholesterol acyltransferase) inhibitory properties that was discussed below.

ACAT is involved in the intestinal absorption of cholesterol and cholesterol ester accumulation in atherogenesis. It is anticipated that ACAT inhibition could be beneficial in treating hypercholesterolemia and atherosclerosis [116-118].

**Fig. 9:**
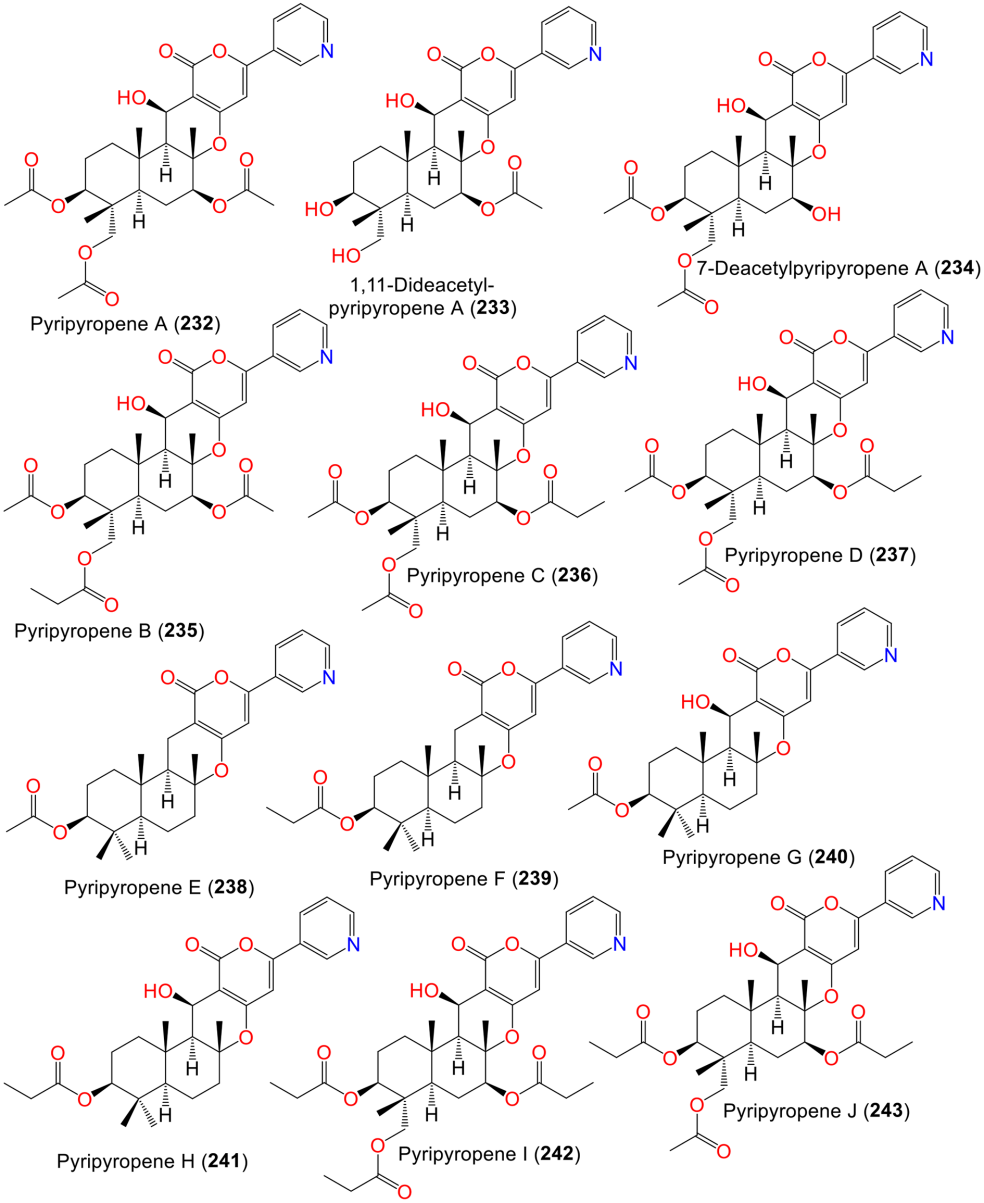
Examples of pyridino-α-pyrone sesquiterpenoids (232–243) reported from *A. fumigatus*.

Omura *et al*. and Kim *et al*. reported the isolation of **232** and **235**–**237** from *A. fumigatus* FO-1289 [119, 120]. In **235**–**237**, one of the three acetyl groups in **232** is substituted by a propionyl moiety (Fig. 9). Compounds **235**–**237** were potent ACAT inhibitors with IC_50_ 53–268 nM [119]. Additionally, studies by Tomoda *et al*. reported the isolation and characterization of **232** and **235**–**251** from the soil associated *A. fumigatus* FO-1289 collected from Jingugaien, Sinjuku-Ku, Tokyo, Japan using HLPC/SiO_2_/RP-18/Sephadex LH-20 CC and spectral tools, respectively [116-118]. The assay of their ACAT inhibitory properties using rat liver microsomes revealed that **232**, **236**, **235**, **243**, and **245** (IC_50_s 0.15, 0.16, 0.27, and 0.85, respectively) demonstrated the most potent inhibitory activity, indicating that they represent the most potent naturally ACAT inhibitors. Also, **242**, **244**, and **246** had potent inhibition (IC_50_ 2.45, 2.65, and 3.80 μM, respectively), however, **247**–**251** were moderately active (IC_50_ 11.0–78.0 μM) in comparison to CL-283,546, (IC_50_1.3 μM) [116-118]. Ohshiro *et al*. stated that **232** (doses 10 to 100 mg/kg) significantly inhibited intestinal cholesterol absorption (%inhibition 30.564.7% to 55.863.3%) in mice. These findings revealed that **232** also reduced cholesteryl oleate and cholesterol levels in both VLDL and LDL in atherogenic mice, resulting in the prevention of atherosclerosis development [121]. Therefore, pyripyropenes as ACAT2 inhibitors appear as potential candidates for the novel antiatherogenic agents. Zou *et al*. stated that **232**, **233**, and **238** reported from HQD24 strain isolated from *Rhizophora mucronata* flower displayed no notable effectiveness against Con A-induced T cell proliferation, as well as no cytotoxic capacity against HepG2 cell line in the cell counting kit-8 (CCK-8) and MTT assays, respectively [47].

Another pyripyropene-related analogs: **252**–**254** were identified by Jeong *et al*. from *A. fumigatus* F37 obtained from soil, Mountain Dukyou/Cheonbuk/Korea culture broth acetone extract using SiO_2_CC/reverse phase HPLC (Fig. S17). These compounds **252**–**254** prohibited ACAT (IC_50_s 94, 40, and 42 μM, respectively), compared to pyripyropene A **232** (IC_50_ 43 nM) [122, 123].

Different members of the sesquiterpene chemical family were reported from *A. fumigatus* (Table S6) [39-41, 46, 48, 53, 56, 71, 124-129]. Among them, **255** was initially separated as antiparasitic metabolite [39, 41, 124]. This compound and its analogs were identified as very effective angiogenesis inhibitors that effectively suppressed endothelial cell proliferation in-vitro and prevented angiogenesis generated by tumors *in-vivo*. Fumagillin was found to reverse the Vpr growth inhibitory effect in human cells and yeast and also suppressed the Vpr-dependent viral gene production in human macrophages, following infection. Interestingly, the semisynthetic analogs of fumagillin showed potent antitumor activity such as TNP-470 is in Phase I and II clinical trials for brain, prostate, and breast cancer treatment, as well as CKD-732 also displayed synergistic antitumor capacity when co-administered with chemotherapeutic agents [33].

In 2004, Jiao *et al*. reported a new fumagillin derivative, **256**, along with **255** from the CANU-A151 strain obtained from saline lake sand/Western Australia [41]. A new metabolite, **257**, along with **255** were isolated from the mycelia EtOAc extract by SiO_2_ CC/HPLC and identified spectroscopic tools. Compound **257** is related to **255** in which the exomethylene moiety is replaced by spiro-epoxide moiety. This compound (IC_50_ 250 nM) possessed >31-fold decrease in antitumor activity versus SKMEL-5 cells in comparison to **256** (IC_50_ 8 nM), suggesting the spiro-epoxide moiety reduced the activity [124]. Compounds **255** (MIC 2.5 μM), **273**, and **281** (MICs 1.25 μM) revealed notable potential versus MRSA, compared to vancomycin (MIC 1.00 μM) [39].

Kim *et al*. isolated novel angiogenesis inhibitor, **260** from *A. fumigatus* IMI-069714 and its structure was assigned as (3*R*,4*R*,6*R*)-4-[(2*R*,3*R*)-2-methyl-3-(3-methyl-but-2-enyl)-oxiranyl]-1-oxa-spiro[2,5]octan-6-ol that was assured by independent synthesis from **259** [127] (Fig. S18). It is noteworthy that its carbamoyl derivative, 6-O-(chloroacetylcarbamoyl)-5-demethoxyfumagillol (IC_50_ 1.147 μM) exhibited a 7-fold greater anti-proliferation potential than **260** (IC_50_7.06 μM) against CPAE (calf pulmonary artery endothelial) cells compared to TNP-470 (IC_50_ 0.0011 7 μM) [127].

Liu *et al*. reported a new sesquiterpenoid, **262** from *A. fumigatus* obtained from coastal saline soil using SiO_2_/Sephadex LH-20 CC/HPLC. Its configuration was specified as 1*R*/2*S*/4*S*/5*R* by X-ray analysis. Compound **262** (IC_50_s 12.4 and 22.1 μM, respectively) was cytotoxic toward HL-60 and A-549 cell lines in the MTT method [46].

Two undescribed sesquiterpenes: **263** and **264** were characterized from *Ligusticum wallichii*-associated *A. fumigatus* fermentation broth using spectral/ECD calculations [53]. Compounds **263** and **264** are trans-cis isomers. In the MTT method, **263** and **264** displayed moderate growth inhibitory potential against MDA-ME-231 and MV4-11 (IC_50_s 14.3/17.3 and 8.4/11.2 μg/ml, respectively) [53]. Compound **265**, a new bergamotane sesquiterpene with 6-methylbicyclo[3.1.1] heptane skeleton was specified from Aconitum-derived M1 strain using spectral/ECD/NMR computational methods to have 8R/10R configuration and optical rotation -7.5. It possessed no notable (Conc. 40 μM and 256 μg/ml, respectively) cytotoxic and antibacterial effects [48]. Another study by Sang *et al*. revealed the isolation of **266**, a new bergamotane sesquiterpenoid from *A. fumigatus* accompanied with *Delphinium grandiflorum* that demonstrated cytotoxic property against A-549, Hela, and HepG2 (inhibition rates 25.46–31.90%) [40].

Coculturing assists in discovering the processes underlying fungal interspecific relationships and novel gene functions, in addition to inducing the biosynthesis of various enzymes and secondary metabolites [128]. In 2023, Su *et al*. revealed that the coculture of *Paraphaeosphaeria* sp. and *A. fumigatus* produced novel fumagillol analogs, compounds **268**–**270**, which were isolated using SiO_2_/Rp-18/Sephadex LH-20 CC and elucidated by spectral/ECD analyses. Compounds **269** and **270** were similar to **259**, featuring tetrahydropyrane and tetrahydrofuran residue. Compounds **268**–**270** demonstrated antifungal potential versus *A. alternata* and *Paraphaeosphaeria* sp.(MICs 2.0–128.0 μg/ml), whereas **268** exhibited potent efficacy versus *A. alternata* (MIC 2.0 μg/ml), compared to nystatin (MICs 2.0 μg/ml) [128].

Jiang *et al*. identified **271** a bisabolane sesquiterpenoid with novel bicyclo[3.2.1]octane ring via methyl transformation configuration using spectral/DP4+ probability/ECD calculations [56]. In 1984, compound **272**, a new meroterpenoid, was isolated as colorless crystals from the IFM 4482 strain that was identified by spectral/Xray analyses. It was proposed to be biosynthesized from bis-C-methylated tetraketide and farnesyl pyrophosphate. This compound at a dose of100 mg/kg (I.P.) showed no toxicity to mice [129].

### Triterpenoids

Compound **273** belongs to fusidane-type antibiotics that represent a unique class of microbial metabolites with penta- or tetracyclic skeletons (Fig. S19). Compound **273** possessed antifungal efficacy versus *B. cinerea*, *A. solani*, *A. alternata*, *C. gloeosporioides*, *F. solani*, *F. oxysporum* f. sp. *niveum*, *F. oxysporum* f. sp. *vasinfectum*, and *G. saubinettii* (MICs 6.25–25 μg/ml), compared to carbendazim and hymexazol [34]. Compound **273** also displayed activity against *S. aureus* (MIC 1.95 μg/ml), compared to ciprofloxacin in the microplate dilution method [76]. Xu *et al*. reported **273** from CUGBMF-170049 strain demonstrated powerful antibacterial potential against *S. aureus* and MRSA (MIC 0.78 μg/ml) [92]. Liu *et al*. stated its antifungal capacity versus *C. albicans* and *T. rubrum* (MIC 31.5 μg/ml) which is comparable to ketoconazole (MIC 31.5 μg/ml) [42]. It also had powerful antibacterial capacity versus MRSA and *S. aureus* (MIC 3.12 μg/ml) comparable to vancomycin and cytotoxic capacity against A-549, Hela, and HepG2 (inhibition rates 30.54–69.12%) [40].

New helvolic acid derivatives: **277**–**286**, along with **276** and **285** were characterized from the marine sponge-derived HNMF-0047 strain collected from Wenchang Beach/Hainan Province/China using spectral/ECD analyses and quantum ECD calculations. Compounds **273**, **281**, **282**, and **286** revealed antibacterial potential against *S. agalactiae* (MICs 8.0, 16.0, 2.0, and 64.0 μg/ml, respectively), while **273**, **281**, and **282** had activity against *S. aureus* (MICs 16.0, 16.0, and 8.0 μg/ml, respectively), compared to tobramycin (MIC 32.0 and 1.0 μg/mL, respectively). It was noted that the existence of a C-21/C-16 lactone ring remarkably diminished the antibacterial capacity, while **282**, with a propionyloxy replacing the C-6 acetoxy substituent in **273**, possessed powerful antibacterial potential versus *S. agalactiae* than tobramycin and **273** [130]. Further, **281** and **282** displayed antibacterial potential against *S. aureus* and MRSA (MIC 6.25–25 μg/ml), however, **275** was inactive, indicating the α,β-unsaturated ketone had a key role in activity [92].

Besides, compound **277** markedly prohibited the proliferation of ConA-caused T and LPS-produced B murine spleen lymphocytes (IC_50_s 12.11 and 62.66 μM, respectively), compared to cyclosporin A (IC_50_ 4.39 μM) [72]. Liang *et al*. reported **287**, a new helvolic acid analog with a phenanthrene core from *A. fumigatus*-harboring *Diphylleia sinensis*. This compound (IC_50_ 139.9 μM) had weak cytotoxic action in the MTT assay against the HepG2 cell line [44].

A study by Liu *et al*. revealed that the bio-guided isolation of the cytotoxic fermentation broth extract of *A. fumigatus* isolated from the *Cleidion brevipetiolatum* roots afforded new tetracyclic triterpenoids: **283** and **284**. These compounds are structurally related to **273** with additional propionyl and acetyl groups connected at C-16-and C-6 OH groups in **283** and C-6- and C-16-OH in **284**. Compounds **283** and **284** possessed cytotoxic potential towards gastric cancer (HGC-27), A-549, and lung adenocarcinoma (H1975) (IC_50_s 8.4–20.9 and 9.8–26.5 μM, respectively), compared with adriamycin (IC_50_ 0.42–0.62 μM) in the MTT assay [95].

Hopane triterpenoids are pentacyclic triterpenes with an icosahydro-1H-cyclopenta-[a]chrysene framework. Many hopanoids with varied structures and promising bioactivities are reported from fungi, bacteria, lichens, plants, and ferns. A study by Ma *et al*. reported the isolation of rare hopane-type triterpenoid glycosides: **288** and **289** from *A. fumigatus* CEA17.1 by Rp-18/Prep-TLC (Fig. S20). It was indicated that **288** had a substantial role in protecting *A. fumigatus* against ultraviolet or heat stress [131].

The bioinformatics analysis and genome sequencing of *A. fumigatus* identified *afum* potential gene cluster for hopane-type glycoside biosynthesis that consists of four genes: *AfumB* (cytochrome P450), *AfumA* (squalene hopane cyclase), *AfumC* (glycosyltransferase), and *AfumD* (transcription factor) [131]. The biosynthesis of **288** was established using a combination of *in-vivo* gene deletion techniques and heterologous expression (Scheme S3). Biosynthesis starts with the hopene skeleton formation from 2,3-oxidosqualene that was catalyzed by squalene hopene cyclase (*AfumA*). Then, cytochrome P450 (*AfumB*) undergoes C-24 hydroxylation and C-30 oxidations to produce **I**. C-24 Glycosylation is achieved by glycosyltransferase (*AfumC*) to yield **288** [131]. This compound was reported to enhance the ultraviolet (UV) resistance and thermotolerance of *A. fumigatus* [131]. Also, **292** is a pentacyclic triterpenoid with 3*S*/4*S*/5*R*/8*R*/9*R*/10*R*/12*R*/13*R*/14*R*/17*S*/18*S*/21*S*/22*S* and +30 optical rotation that was moderately active against *F. graminearum* and *V. alginolyticus* (MICs 32 and 16 μg/mL, respectively) [29].

Limbadri *et al*. characterized a new sterol, **293** from deep-sea-derived *A. fumigatus* SCSIO-41012. Compound **293** showed similarity to 6-deacetyl-3-ketocephalosporin P1, except for C-7 ketone carbon instead of an oxygenated methine [70]. Rateb *et al*. separated **295** from *A. fumigatus* MBC-F1-10/*Streptomyces bullii* coculture [35] that exhibited moderate activity against *S. aureus* (MICs 15.63 μg/ml) [76, 132]. Compounds **295** and **300** isolated from *A. fumigatus* AR05-accompanied with *Astragalus membranaceus* root had weak and no antibacterial and antifungal activity, respectively against *B. subtilis*, *S. aureus*, *E. coli*, *S. typhimurium*, *C. albicans*, *P. chrysogenum*, and *F. solani* [36]. Also, **295**–**299** were reported from CY018 and WJ-131 strains associated with *Cynodon dactylon* leaves [42] and *Gardenia jasminoides* stem, respectively (Table S7; Fig. S21) [56].

### Anthrone, Quinone, and Anthraquinones

Studies by Yamamoto *et al*. 1965 reported the separation of quinones: **302**–**310** from both *A. fumigatus* DH 413 and J-4 strains using SiO_2_ CC that were identified by NMR (Table S7). Also, **302** and **311** were identified from the mycelia of *A. fumigatus* DH 413 culture [133, 134]. These metabolites were proposed to be biosynthesized from orsellinic acid through different reactions, including methylation, hydroxylation, epoxidation, oxidation, and decarboxylation (Scheme S4) [133, 135, 136]. Interestingly, **305** originated only when NaCl was added to the culture medium [134].

New nematicidal quinones: **313** and **314**, together with **306** were isolated from *A. fumigatus* culture obtained from soil by SiO_2_ CC/preparative TLC (Fig. S22). Compound **313** possessed notable nematocidal properties (%inhibition 54 and 24%%) versus *P. penetrans* and *B. xylophilus* without inhibiting plant growth except for lettuce seedlings, whereas **306** and **314** (31% and 44%, respectively) were active against *B. xylophilus* [89]. Antibacterial activity revealed that **315** (MIC 1.25 μg/ml) possessed notable antibacterial effectiveness versus *M. bovis* *bacillus* Calmette-Guerin, compared to rifamycin (MIC 0.02 μg/ml) [75]. Additionally, **317** and **318** were reported from *A. fumigatus* Wrq12 [44].

A study by Yamamoto *et al*. investigated the yellow pigment produced by *A. fumigatus* J-4 and revealed the isolation of chlorinated metabolites: **322**–**326**, along with **315** from the mycelium extract using SiO_2_ CC eluted with benzene and benzene: ether solvent systems, whereas the anthrone derivative gave deep green colour with p-nitroso dimethylaniline reagent (Fig. S23). These compounds were identified using chemical and spectral tools [132].

Activating pcPTase (polycyclic polyketide prenyltransferase)-having silent clusters in *A. fumigatus* resulted in a new prenylated anthracenone; **328** that was assigned by X-ray/NMR analyses and has a 3,4-dihydroanthracen-1(2H)-one tricyclic skeleton with a 2,4-keto-enol pentyl side, C2-COCH_3_ substituent *syn* to a C3-OH, and C5-dimethylallyl side chain. Compound **328** possessed T-cell antiproliferation potential (IC_50_ 2.99 μM), compared to cyclosporin A (IC_50_ 26 nM), while it was less toxic to HeLa and HFF cell lines (> 50 μM), suggesting its potential as an immunosuppressive agent and specificity to T-cells [137].

From soil-inhabiting *A. fumigatus* 3T-EGY, **327**, **329**, and **330** were reported by Abdel-Aziz *et al*. Compound **330** had a moderate antimicrobial potential against *S. aureus*, *P. aeruginosa*, and *C. albicans* (IZDs ranging from 9.0 to 10.66 mm), compared to neomycin (IZDs ranging from 14.0 to 19.0 mm) [115].

### Benzophenones and Diphenyl Ethers

Natural benzophenones are a class of chemical compounds reported from the plants of various families such as iridaceae, garciniaceae, lauraceae, moraceae, rosaceae, daphneceae, and other families [139]. They are made up of two benzene rings joined by a carbonyl group and have various substituents, including hydroxyl, methoxy, glycosyl, and isopentenyl, which are the intermediary of xanthones. These organic compounds exhibit a wide range of biological properties, including anti-inflammatory, anti-allergy, α-glucosidase inhibition, and cardiovascular protection [138].

New sulfur-bearing benzophenones, **332**–**335**, along with **336** and **337** were separated from cave soil-derived *A. fumigatus* GZWMJZ-152. Their structures and configurations were elucidated by spectral/X-ray/ECD calculations [110]. Compound **332** features an unusual skeleton of diketopiperazine and benzophenone cores connected through a thioether linkage, while **333** has a rare sulfoxide group. Both **333** and **334** were initially separated as racemic mixtures that were purified to (+)-**333**/(-)-**333** and (+)-**334**/(-)-**334**, respectively. In the DPPH assay, **336** scavenged DPPH radicals (IC_50_ 18.90 μM), while (±)-**333**, (+)-**333**, (-)-**333**, **335**, **336**, and **337** displayed powerful antioxidant properties (oxygen radical antioxidant capacity (ORAC) values 1.73, 1.76, 1.59, 1.65, 6.14, and 1.55 μM TE/μM, respectively). Additionally, (±)-**333** and (±)-**334** revealed protective action against PC-12 cells oxidative injury induced by hydrogen peroxide (H_2_O_2_). Interestingly, the racemic mixtures had better activity than pure enantiomers **333** and **334** [110].

A study by Zhang *et al*. reported that **336** and **337** (MIC 1.25 μM) possessed prominent antibacterial capacity versus MRSA, compared to vancomycin (MIC 1.00 μM). Therefore, *A. fumigatus* can biosynthesize bio-metabolites with anti-MRSA activity [39].

The new benzophenone: **340** which is the methoxylated derivative of **337** was identified from *Cynodon dactylon*-accompanied CY018 strain [42]. Liu *et al*. reported the characterization of new benzophenone: **342** using spectral tools from sea sediment-derived *A. fumigatus* SZW01 collected from Shenzhen/Guangdong province/China (Table S9) (Fig. S24). This compound demonstrated weak and strong free radical scavenging capacity in the DPPH and ABTS assays, respectively, compared to vitamin C (IC_50_s 25.13 and 12.5 μM, respectively). Whilst it showed a powerful a-glucosidase inhibition potential than acarbose [84].

New diphenyl ethers **344** was identified from the cytotoxic extract of *A. fumigatus* associated with *Heteroscyphus tener* liverwort that showed weak cytotoxic effectiveness against PC3, PC3D, A-549, and NCI-H460 cell lines in the MTT [45]. Additionally, **346** was separated from *A. fumigatus* DH 413 culture treated with methionine antagonist; DL-ethionine using SiO_2_ CC and crystallization from benzene; this compound gave green, red, and violet colors with Gibbs, Millon, and FeCl_3_ reagents, respectively, and was assigned by spectral and chemical methods [133].

### Chroman and Isochromane Derivatives

Chromans are rare metabolites reported from fungi. Some published work reported the isolation of chromane and isochrome derivatives from *A. fumigatus* (Table S10) [138, 72, 85, 115, 139-143].

In 1996, Culer *et al*. separated new chromans; **347** and its methyl ester **348** from *A. fumigatus* associated with a coral lichen collected from hot, sulfurous springs environment of Craters-of-the-Moon/North Island of New Zealand utilizing SiO_2_ CC and identified by spectral/ chemical methods [139]. Thakur *et al*. stated that the MeOH extract of *A. fumigatus* obtained from *Bacopa monnieri* prohibited *M. tuberculosis* H37RV growth (MIC 500 μg/ml). From this extract **364**, an isochromenone was isolated using SiO_2_ CC and characterized by spectra/Xray analyses [142]. Additionally, new isochromenes, **357** and **358**, along with **359**-**363** were identified from cigar tobacco-associated *A. fumigatus*. Compounds **357** and **358** feature isochromen core with C-6 attached propanoyl and 3-hydroxypropanoyl units, respectively. Compounds **357** and **358** (MIC_50_s 6.8 and 8.4 μg/ml, respectively) revealed antibacterial capacities against *Pseudomonas syringae* (causative pathogen of tobacco angular spot disease), compared to streptomycin (MIC 2.2 μg/ml) in the broth microdilution method [141].

The investigation of *A. fumigatus* from *Edgeworthia chrysantha* coastal plant resulted in the identification of **350**–**356** and **368** (Figs. S25 and S26). These metabolites showed no notable inhibitory efficacy towards *E. coli*, *S. aureus*, and *C. albicans* in broth microdilution assay [140].

Hua *et al*. proposed the biosynthetic pathway for aromatic polyketides: **351**–**356** [140]. Six malonyl-CoA and one acetyl-CoA are employed for the biosynthesis of **350**, **368**, and intermediates (A–D) by sequential catalytic reaction in a non-reducing PKS system (Scheme S5). Further, dimerization of **350** with A–D at C-6, C-7, C-9, or C-10 leads to the formation of **355**–**356**. It was proven that D8.t287 is the key PKS gene accountable for the biosynthesis of heptaketone, the initial precursor of aromatic polyketides [140].

### Azaphilone and Pyranone Derivatives

A new azaphilone derivative: **371**, and known analogs **370**, **372**, and **373** were separated from gorgonian-derived *A. fumigatus* 14–27 EtOAc culture extract (Table S11) [144].

These metabolites displayed high-performance liquid chromatography- Ultraviolet (HPLC-UV) absorptions at 340, 264, and 216 nm. Compound **371** features azaphilone core and orsellinic acid part as **370** which was previously reported from *Talaromyces aculeatus* DS-620137. The difference between them is the shift of H-8, suggesting **371** is an epimer of **370**. The configuration of **371** was specified as 7R/7R/8aS based on ECD and optical rotation (Fig. S27). None of these compounds had antibacterial or cytotoxic capacities versus *S. albus*/*S. aureus*/*V. anguillarum*/*E. coli*/*P. aeruginosa* and HCT-116/HL- 60/HepG2/A-549 in the broth dilution and MTT methods, respectively [144].

Bioactivity-guided separation of *Acrostichum specioum*-associated *A. fumigatus* JRJ111048 EtOAc extract that possessed insecticidal capacity against *Spodoptera litura* led to the identification of **374** that is a new anhydride derivative, along with **375**–**378**. These compounds were examined *in-vivo* against *S. litura* newly hatched larvae. Compound **374** revealed potent insecticide potential (Conc. 20 μg/ml) and reduced larval growth compared to controls with mortality rates 40, 46.67, 60, and 76.67%, compared to azadirachtin (mortality rates 86.67, 93.33, 100, and 100%, respectively) at 7, 10, 14, and 20th days, respectively. Only **375** revealed weak antifungal potential towards *C. albicans* (IZD 8.6 mm, Conc. 20 mg/ml), compared to ketoconazole (IZD 29.2 mm) [103]. Compounds **379** and **380** are pyranone derivatives were reported from sponge-associated *A. fumigatus* WA7S6 and *A. fumigatus* D/*Fusarium oxysporum* R1 co-culture, respectively [101, 145].

### Other Metabolites

Various secondary metabolites, including cyclohexanones, naphthalene, furan-containing, aldehyde, phenolics, fatty acids, and related derivatives reported from this fungus were listed in table S12 [13, 34, 38, 39, 44, 45, 48, 50, 58, 71, 72, 75, 76, 97, 100, 101, 103, 115, 143-148].

A new cyclohexenone derivative, **381** along with **382** were obtained from the co-culture of *A. fumigatus*/*Alternaria alternata* associated with *Coffea arabica* using SiO_2_ CC/Rp-18/Sephadex LH-20 that were identified by spectral/ECD analyses. Their absolute configurations were assigned as 2R and 2*R*/3*S*, respectively based on ECD calculations (Fig. S28). Compounds **381** and **382** had weak antifungal potential against *F. incarnatum* and *A. alternata* (MICs 32.0–64.0 μg/ ml) [58].

Compound **386** was separated and identified from *G. griffithii*-associated *A. fumigatus* EtOAc extract using SiO_2_ CC and spectral analyses, respectively [148]. Compound **387** is a toxin belonging to anthraquinone that was separated from *A. fumigatus* NRRL-35693 spores using RP-HPLC/LC-MS and characterized by NMR. This compound was the most toxic metabolite that decreased cell viability and triggered cell lysis (IC_50_ 7 μM) against A-549 and HBEpC (human bronchial epithelial) cells. It initiated the NO (nitric oxide) and H_2_O_2_ (hydrogen peroxide) intracellular formation that triggered necrotic cell death and reduced mitochondrial membrane potential [13]. It also possessed anti-protozoal capacities versus *Toxoplasma gondii* and *Trypanosoma cruzi* [13]. Besides, it demonstrated antibacterial potential versus *B. subtilis* and *M. bovis* *bacillus* Calmette-Guerin (MICs 12.5 and 1.25 μg/ml, respectively), compared to rifamycin (*M. bovis* *bacillus*; MIC 0.02 μg/ml) [75].

A study by Wang *et al*. reported that **389** a powerful nematode-antagonist was successfully obtained from *A. fumigatus* 1T-2 fermentation broth using LC-MS (liquid chromatography-mass spectrometry). This compound is furan carboxylic acid that demonstrated effective mortality activity *in vitro* (LC_50_ 37.75 μg/ml at 24 h) against *Megalaima incognita* as fosthiazate (LC_50_ 30 μg/ml) [147]. Further, detrimental effects on egg hatchability and nematode vitality were noted on continuous exposure to 2-furoic acid. Additionally, Wang *et al*. found that **389** and 1T-2 fermentation broth substantially prohibited *M. incognita* in the greenhouse test-tube assay [147]. Thus, **389** could be a useful lead for developing affordable and eco-friendly new nematicides [147].

Brazilian soil-accompanied *A. fumigatus* IFM 54246, new acetophenone, **391** was separated from MeOH: CH_2_Cl_2_ (dichloromethane) (1:1) extract using SiO_2_ CC/HPLC that had a broad antifungal potential versus *A. fumigatus*, *A. niger*, *C. albicans*, and *C. neoformans* [68]. Gill *et al*. reported that soil-derived *A. fumigatus* had the capacity to biosynthesize **393** in high yield (18 mg/l). Interestingly, **393** possessed strong inhibitory potential against *A. oryzae*, *T. citrinoviride*, *G. lucidum*, *P. ostreatus*, and *Aspergillus* sp. (%inhibition ratios 13–87%). These findings suggested the use of this fungus for industrial production of **393** [100]. Also, compounds **394**, **398**, and **402** were isolated from the soil-derived 3T-EGY strain [115].

A new antibiotic, **395** was isolated by RP-18 CC and identified by NMR spectral tools from *A. fumigatus* Y-83,0405 obtained from Himalayan soil sample (Table S11). Compound **395** gave a violet color with ninhydrin and decolorized dilute KMnO_4_ solution. Its structure was established as 2-amino-4-acetoxy-3,5,14-trihydroxy-Δ^6^-eicosenoic acid. This compound had potent antifungal potential versus *C. beticola* and **C. resinae** (MICs 0.9 μg/ml), followed by *A. nigar* and *P. digitatum* (MICs 7.8 μg/ml) [143].

Compounds **399** and **400** were identified by GCMS to which the extract antioxidant capacity was attributed [145], while **398**, **403**, and **404** were isolated from *A. fumigatus* D/*F. oxysporum* R1 co-culture [101] (Fig. S29).

## Conclusion

**Fig. 10:**
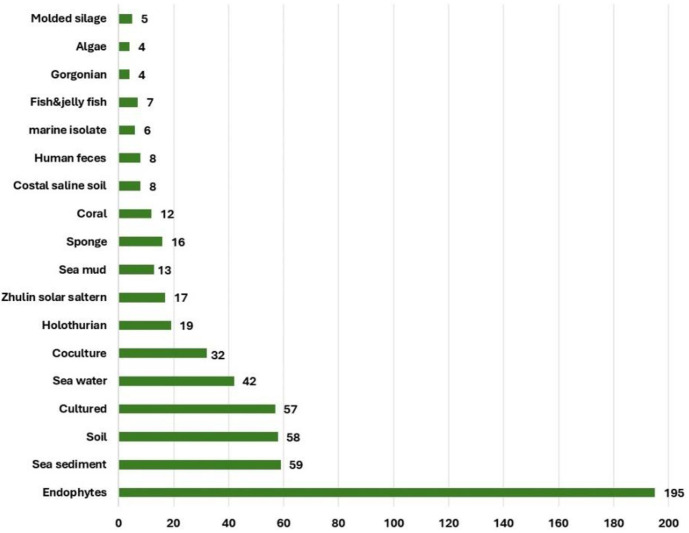
Number of metabolites reported from *A. fumigatus* isolated from various sources.

*A. fumigatus* emerges as a complex organism, known for its dual roles as a potent pathogen and a promising source of bioactive compounds. While its role in human infections, especially in immunocompromised patients, has been the focal point of many studies, this review highlights over 400 compounds reported from *A. fumigatus* strains that were isolated from various sources, showcasing its immense potential as a natural source of valuable secondary metabolites (Fig. 10). The reported data reveals that *A. fumigatus* thrives in a wide variety of environments, from terrestrial (*e.g.*, soil, endophytes) to marine ecosystems (*e.g.*, sea sediment, seawater). Endophytes contribute by the largest number of compounds (195), while the marine environment (190 compounds) (sea sediment, seawater, sea mud, marine isolates, etc.) also provides substantial sources of *A. fumigatus* compounds with a high number of compounds from sea sediment (59) and seawater (42) associated strains. The coculture of *A. fumigatus* alongside other microorganisms contributed 32 compounds, suggesting that microbial interactions can stimulate the production of unique bioactive compounds, which may not be produced in monoculture. Therefore, coculturing is a valuable strategy in drug discovery for uncovering cryptic secondary metabolites.

**Fig. 11:**
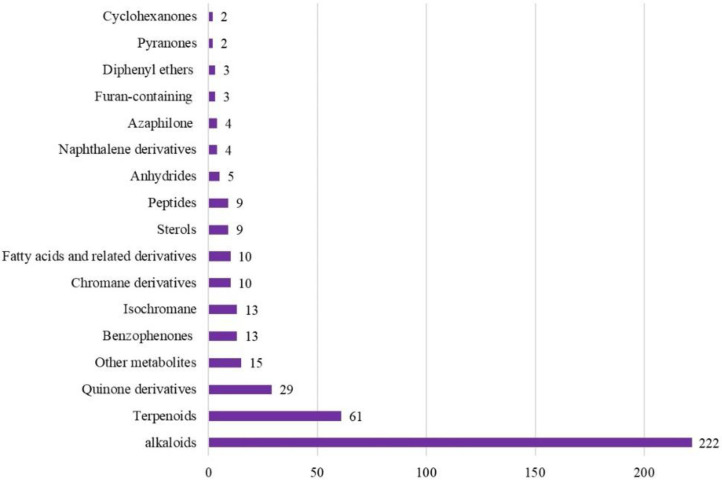
Different classes of secondary metabolites reported from *A. fumigatus*.

These metabolites include alkaloids, terpenes, quinones, peptides, and sterols. Among the major metabolites of *A. fumigatus*, alkaloids (222 compounds) stand out as the most abundant group, followed by terpenoids (61 compounds) and quinone derivatives (29 compounds) (Fig. 11).

These compounds have been assessed for a wide range of biological properties, including antimicrobial, cytotoxic, anti-inflammatory, ACAT inhibitory, and antioxidant activities, whereas some compounds showed marked effectiveness. For example, alkaloids such as **3, 5, 25, 29, 37, 39, 41, 42, 68, 69, 71, 76, 82, 125, 141, 197**, and **201** exhibited strong antimicrobial activity against a wide range of pathogens, often showing better or comparable efficacy to standard controls. Notably, **5** and **201** demonstrated potent antifungal activity against *C. albican*,*s* with MIC values equal to that of amphotericin B. Furthermore, antibacterial studies of compounds such as **42** revealed effective inhibition of pathogens like *E. coli* and *S. aureus* at MIC values as low as 0.39 μg/ml, comparable to ampicillin. These results indicate that these natural compounds possess broad-spectrum antimicrobial properties and have the potential for further development as therapeutic agents. Compound **76** showed potent antibacterial activity against *G. saubinettii*, *B. subtilis*, *S. aureus*, and *E. coli*, with MIC values comparable to the commonly used gentamicin. Additionally, it exhibited antifungal properties, particularly against *C. albicans* and *Fusarium solani*, with MIC values outperforming those of the control nystatin. Moreover, compounds like **124**, **130**, and **131** demonstrated notable anti-inflammatory effects in LPS-induced NO production assays in RAW264.7 cells, with IC_50_ values lower than the standard indomethacin, while **124** demonstrated a noteworthy anti-inflammatory impact by deactivating the TLR4/MD2 signaling pathway that could be considered for MD2 inhibitors development. Several derivatives of fumitremorgin and spirotryprostatin also showed marked cytotoxicity against various cancer cell lines, with IC_50_ values in the micromolar range. Compound **125** caused marked apoptosis in MCF-7 via mitochondrial cell death pathway activation, suggesting its potential as a breast cancer therapeutic candidate. Additionally, **141** is a unique first-in-class PCSK9-targeting lead that can be used to manage the progression and recurrence of hormone-dependent breast cancer. Compound **328** possessed a marked T-cell antiproliferation capacity than cyclosporin A, suggesting its potential as an immunosuppressive agent. Terpenes, another critical class of metabolites produced by *A. fumigatus*, include pyripyropenes that have potent ACAT2 inhibitory capacities, revealing their potential for developing novel antiatherogenic agents. Fumagillin, a sesquiterpene with notable anti-angiogenic and antiparasitic activities.

It is noteworthy that compound **93** revealed a potent plant growth inhibitory effectiveness, suggesting its potential as a natural eco-friendly herbicide. Compounds **149** and **154** demonstrated marked antiseizure potential, indicating their possible development as lead compounds for treating epileptic seizures. Compounds **172** and **173** showed significant anti-*G. cichoracearum* activity, making them promising candidates for the development of new anti-powdery mildew pesticide. Further, compound **389** is a potent nematode-antagonist that could serve as a lead structure for the development of affordable and eco-friendly nematicides.

In conclusion, *A. fumigatus* represents a multifaceted organism with significant biotechnological and pharmaceutical potential, owing to its diverse classes of secondary metabolites. The adaptability of *A. fumigatus* to various ecological niches and the discovery of unique metabolites through coculture approaches highlight the potential of this fungus in drug discovery, paving the way for further research in this area that could contribute to therapeutic advancements.

## Supplemental Materials

Supplementary data for this paper are available on-line only at http://jmb.or.kr.
