# Supplementary material for: Aspergillus fumigatus from Pathogenic Fungus to Unexplored Natural Treasure: Changing the Concept
Source: J Microbiol Biotechnol. 2025 Mar 11;35:e2411082. doi: 10.4014/jmb.2411.11082 (PMC11925756; doi:10.4014/jmb.2411.11082)
Supplement: Supplementary file 1 [file jmb-35-e2411082-supple.pdf]

J. Microbiol. Biotechnol. 2025. 35: e2411082
https://doi.org/10.4014/jmb.2411.11082

Supplementary Figures and Tables

Aspergillus fumigatus from Pathogenic Fungus to Unexplored Natural Treasure: Changing the Concept

Sabrin R.M. Ibrahim,[1,2*] Hagar Mohamed Mohamed,[3,4] Anfal S. Aljahdali,[5] Samar S. A. Murshid,[6] Shaimaa G. A. Mohamed,[7] Hossam M. Abdallah,[6,8] and Gamal A. Mohamed[6]

[1] Department of Chemistry, Preparatory Year Program, Batterjee Medical College, Jeddah, 21442, Saudi Arabia

[2] Department of Pharmacognosy, Faculty of Pharmacy, Assiut University, Assiut 71526, Egypt

[3] Department of Medical Laboratory Analysis, Faculty of Medical & Health Sciences, Liwa College, Abu Dhabi 41009, United Arab of Emirates

[4] Department of Applied Medical Chemistry, Medical Research Institute, Alexandria University, Alexandria, Egypt

[5] Department of Pharmaceutical Chemistry, Faculty of Pharmacy, King Abdulaziz University, Jeddah, Saudi Arabia

[6] Department of Natural Products and Alternative Medicine, Faculty of Pharmacy, King Abdulaziz University, Jeddah, 21589, Saudi Arabiahmafifi@kau.edu.sa (H.M.A.)

[7] Department of Prosthodontics, Faculty of Dentistry, British University, El Sherouk City, Suez Desert Road, Cairo 11837, Egypt

[8] Department of Pharmacognosy, Faculty of Pharmacy, Cairo University, Cairo 11562, Egypt

* Correspondence: sabrin.ibrahim@bmc.edu.sa or sabreen.ibrahim@pharm.aun.edu.eg (S.R.M.I.)

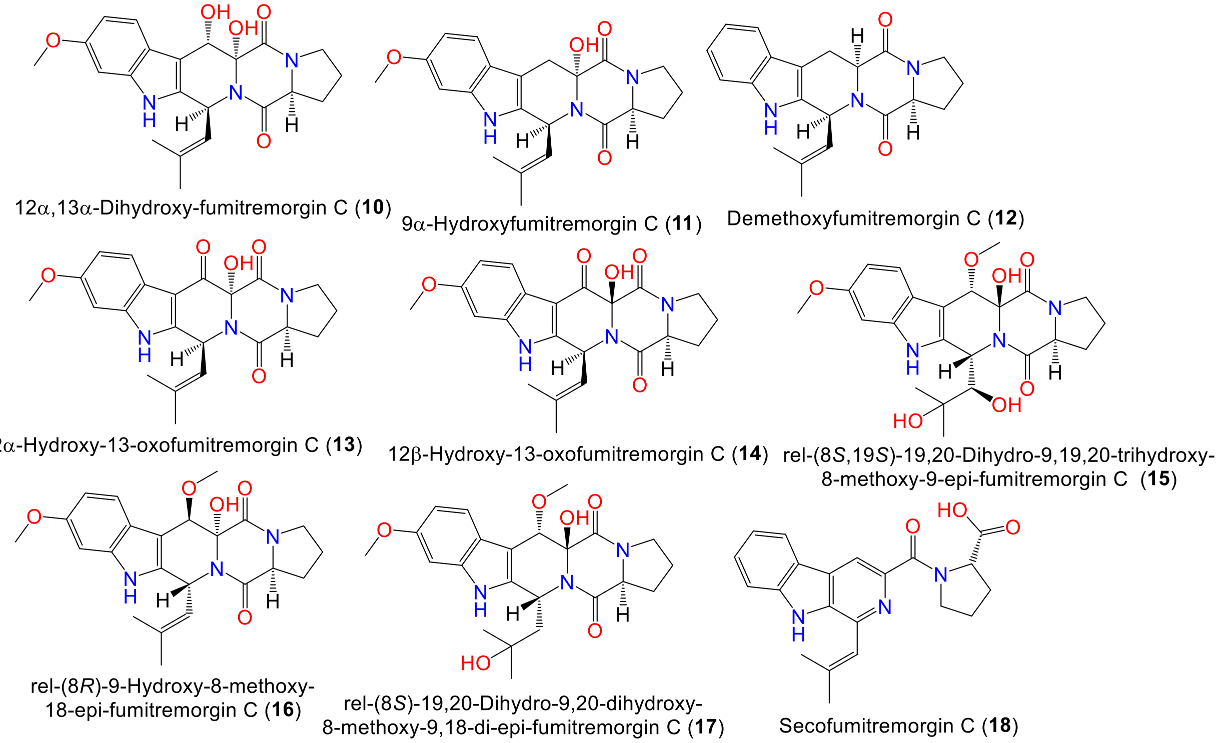

Fig. S1. Chemical structures of indole-diketopiperazine alkaloids (10–18).

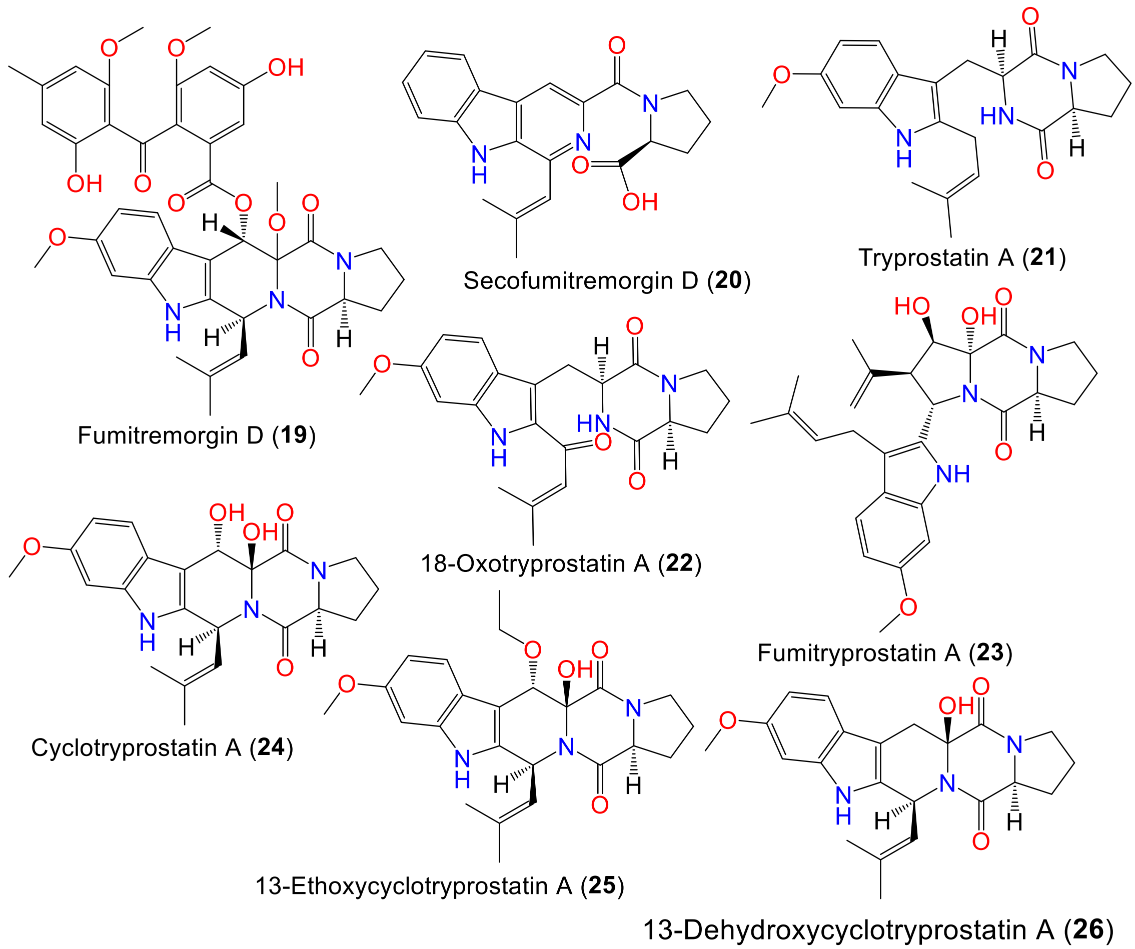

Fig. S2. **Chemical structures of indole-diketopiperazine alkaloids** (**19**–**26**).

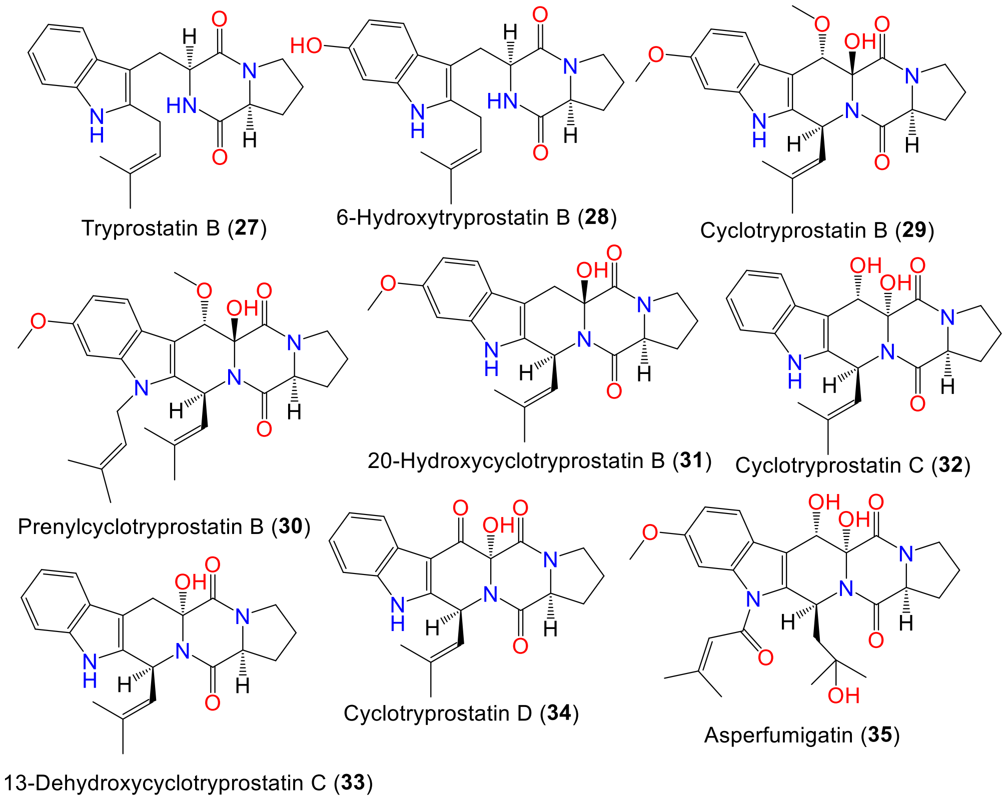

Fig. S3. Chemical structures of indole-diketopiperazine alkaloids (27–35).

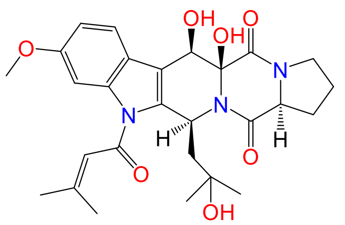

12β,13β-hydroxy-asperfumigatin (**36**)

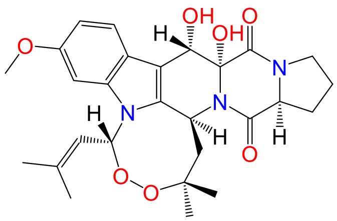

Verruculogen (**37**)

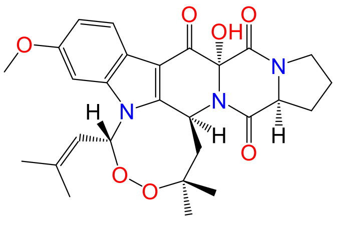

13-Oxoverruculogen (**38**)

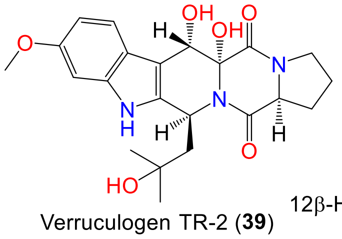

Verruculogen TR-2 (**39**)

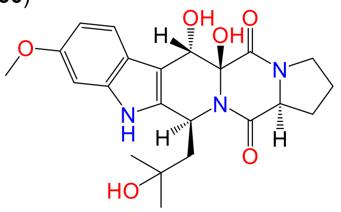

12β-Hydroxyverruculogen TR-2 (**40**)

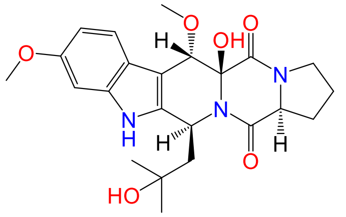

12β-Hydroxy-13α-methoxyverruculogen TR-2 (**41**)

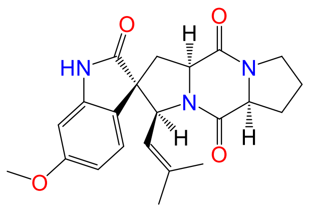

Spirotryprostatin A (**42**)

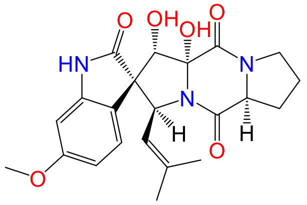

Spiro [5H,10H-dipyrrolo[1,2-a:1`,2`-d]pyrazine-2-(3H),2`-[2H]indole]-3`,5,10(1`H)-trione (**43**)

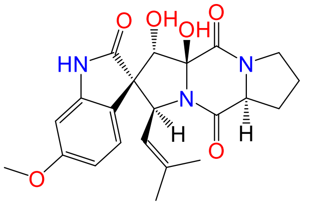

(3*S*,8*S*,9*S*,18*S*)-8,9-dihydroxyspirotryprostatin A (**44**)

Figure S4. Chemical structures of indole-diketopiperazine alkaloids (**36–44**).

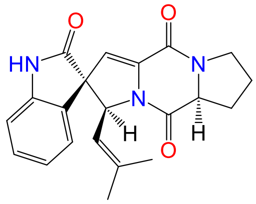

Spirotryprostatin B (**45**)

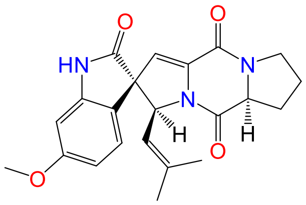

6-Methoxyspirotryprostatin B (**46**) = Spirotryprostatin G

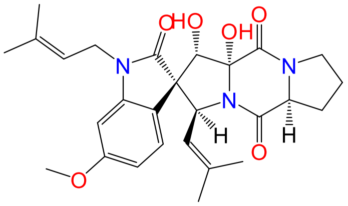

Spirotryprostatin C (**47**)

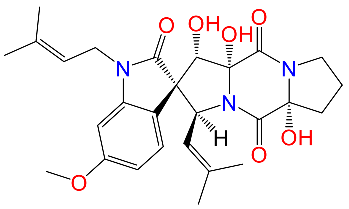

Spirotryprostatin D (**48**)

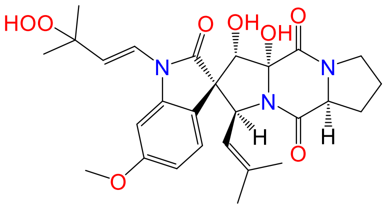

Spirotryprostatin E (**49**)

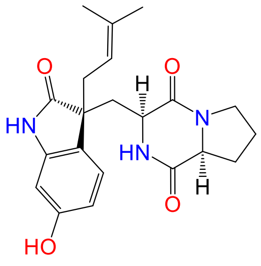

Spirotryprostatin K (**50**)

Fig. S5. Chemical structures of indole-diketopiperazine alkaloids (45–50).

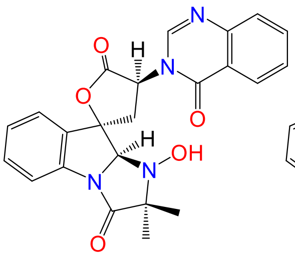

Tryptoquivaline G (FTG) (**60**)

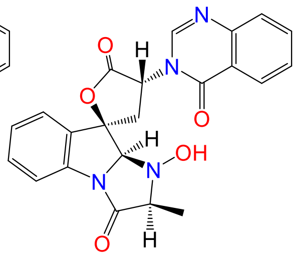

Tryptoquivaline H (FTH) (**61**)

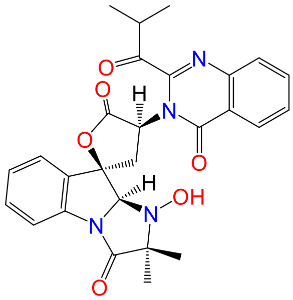

Tryptoquivaline I (FTI) (**62**)

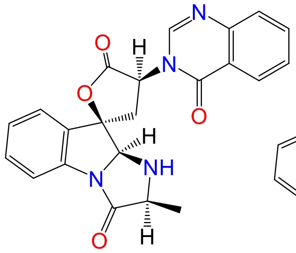

Tryptoquivaline J (FTJ) (**63**)

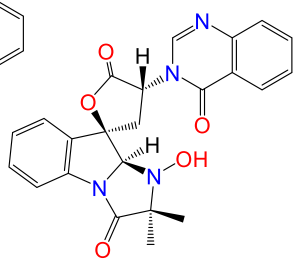

Tryptoquivaline L (FTL) (**64**)

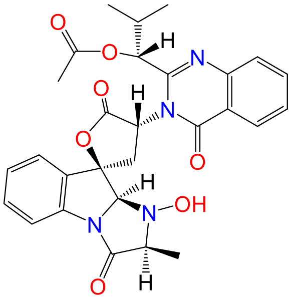

Tryptoquivaline M (FTM) (**65**)

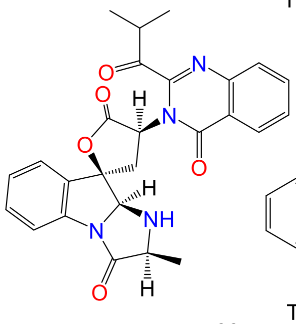

Tryptoquivaline N (FTN) (**66**)

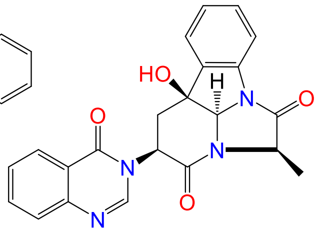

Tryptoquivaline O (FTO) (**67**)

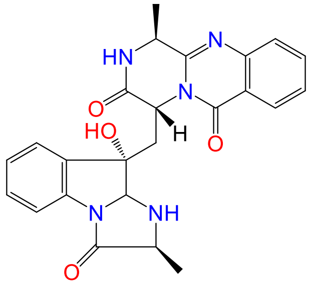

Fumiquinazoline A (**68**)

Fig. S6. Chemical structures of indole-quinazoline alkaloids (60–68).

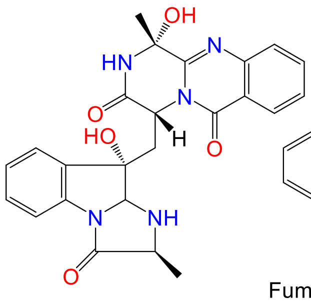

3-Hydroxyfumiquinazoline A (**69**)

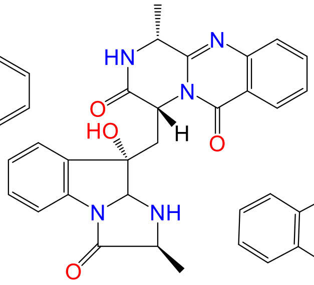

Fumiquinazoline B (**70**)

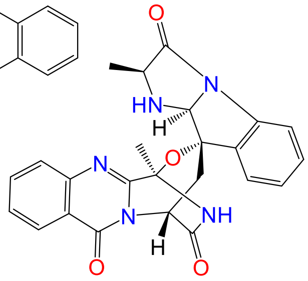

Fumiquinazoline C (**71**)

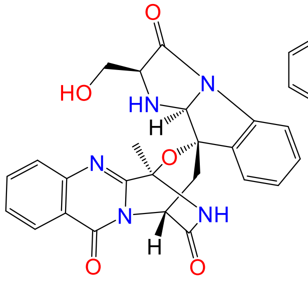

Fumiquinazoline C (**72**)

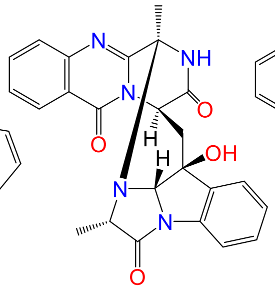

Fumiquinazoline D (**73**)

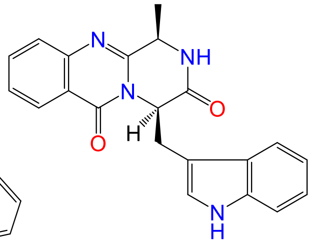

Fumiquinazoline F (**74**)

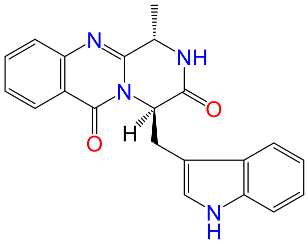

Fumiquinazoline G (**75**)

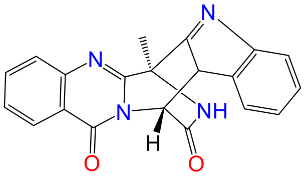

Fumiquinazoline J (**76**)

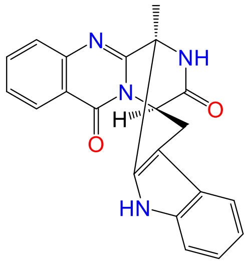

Fumiquinazoline K (**77**)

Fig. S7. Chemical structures of indole-quinazoline alkaloids (69–77).

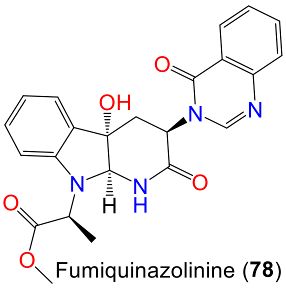

Fumiquinazolinine (**78**)

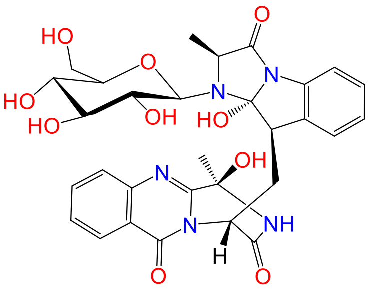

Fumigatoside B (**79**)

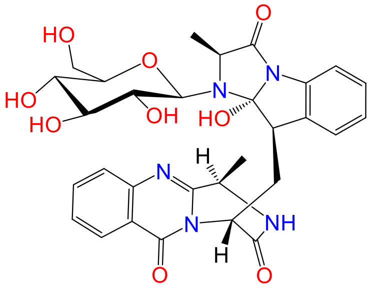

Fumigatoside C (**80**)

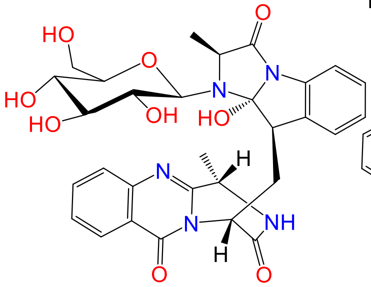

Fumigatoside D (**81**)

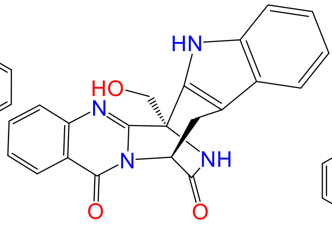

Fumigatoside E (**82**)

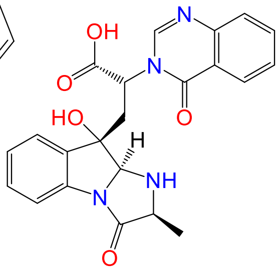

Fumigatoside F (**83**)

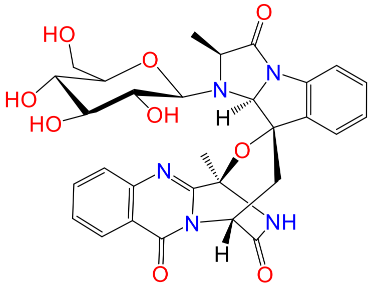

Fumigatoside G (**84**)

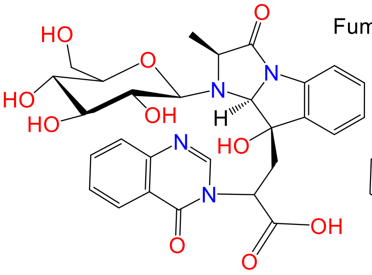

Fumigatoside H (**85**)

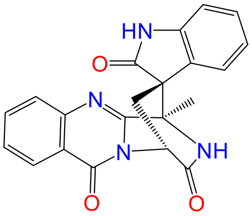

(+)-Alantrypinone (**86**)

Fig. S8. Chemical structures of indole-quinazoline alkaloids (78–86).

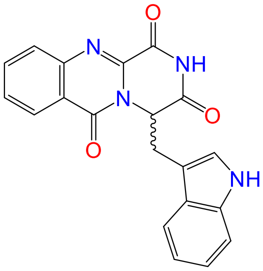

Oxoglyantrypine (**87**)

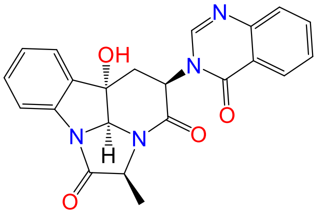

Chaetominine (**88**)

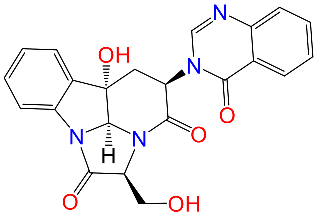

Chaetominine A (**89**)

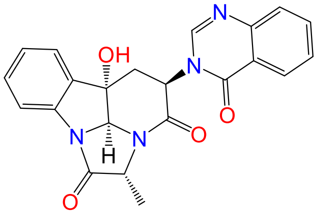

Isochaetominine = 11-*epi*-chaetominine (**90**)

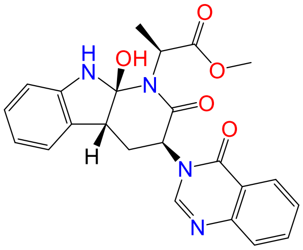

Methyl (S)-{2-((3R,4aS,9aR)-hydroxy-2-oxo-3-(4-oxoquinazolin-3(4H)-yl)-2,3,4,4a,9,9a-hexahydro-1H-pyrido[2,3-b]indol-1-yl)propanoate} (**91**)

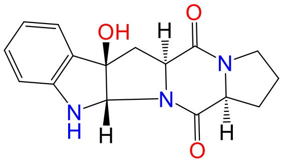

3β-Hydroxy cyclo-L-tryptophyl-L-proline (**92**)

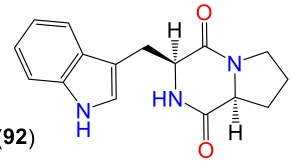

Cyclo-L-tryptophyl-L-proline = Brevianamide F (**93**)

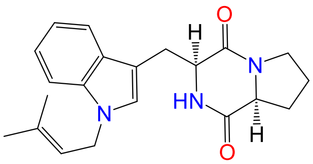

N-Prenyl-cyclo-L-tryptophyl-L-proline (**94**)

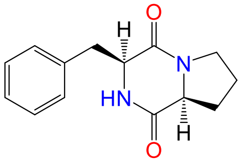

Cyclo (L)-Pro-(L)-Phe (**95**)

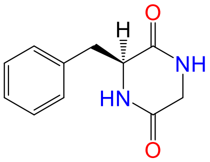

Cyclo-(Gly-Phe) (**96**)

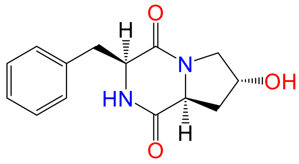

Cyclo (L)-4-OH-Pro-(L)-Phe (**97**)

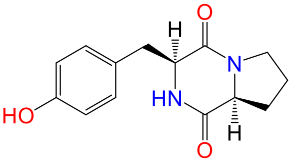

Cyclo-(L-Tyr-L-Pro) (**98**)

Fig. S9. Chemical structures of indole-quinazoline (87–91) and diketopiperazine (92–98) alkaloids.

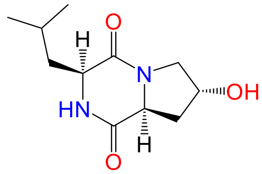

Cyclo (L)-4-OH-Pro-(L)-Leu (**111**)

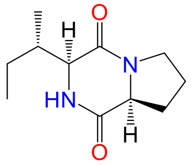

Cyclo (L-isoleucyl-L-prolyl) (**112**)

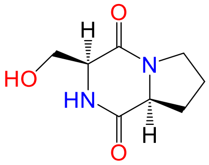

Cyclo-(Pro-Ser) (**113**)

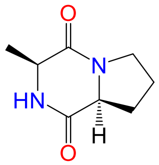

Cyclo-(Pro-Ala) (**114**)

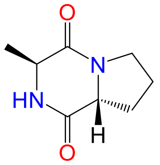

Cyclo-(D-Pro-L-Ala) (**115**)

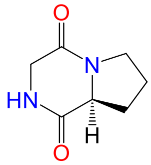

Cyclo (L)-Pro-Gly (**116**)

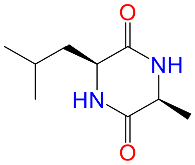

Cyclo (L-Ala-L-Leu) (**117**)

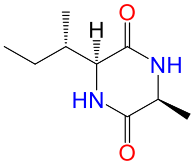

Cyclo (L-Ala-L-Ile) (**118**)

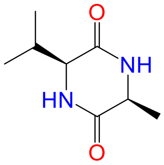

Cyclo-(L-Ala-L-Val) (**119**)

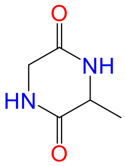

Cyclo-(Gly-Ala) (**120**)

Fig. S10. Chemical structures of diketopiperazine alkaloids (111–120).

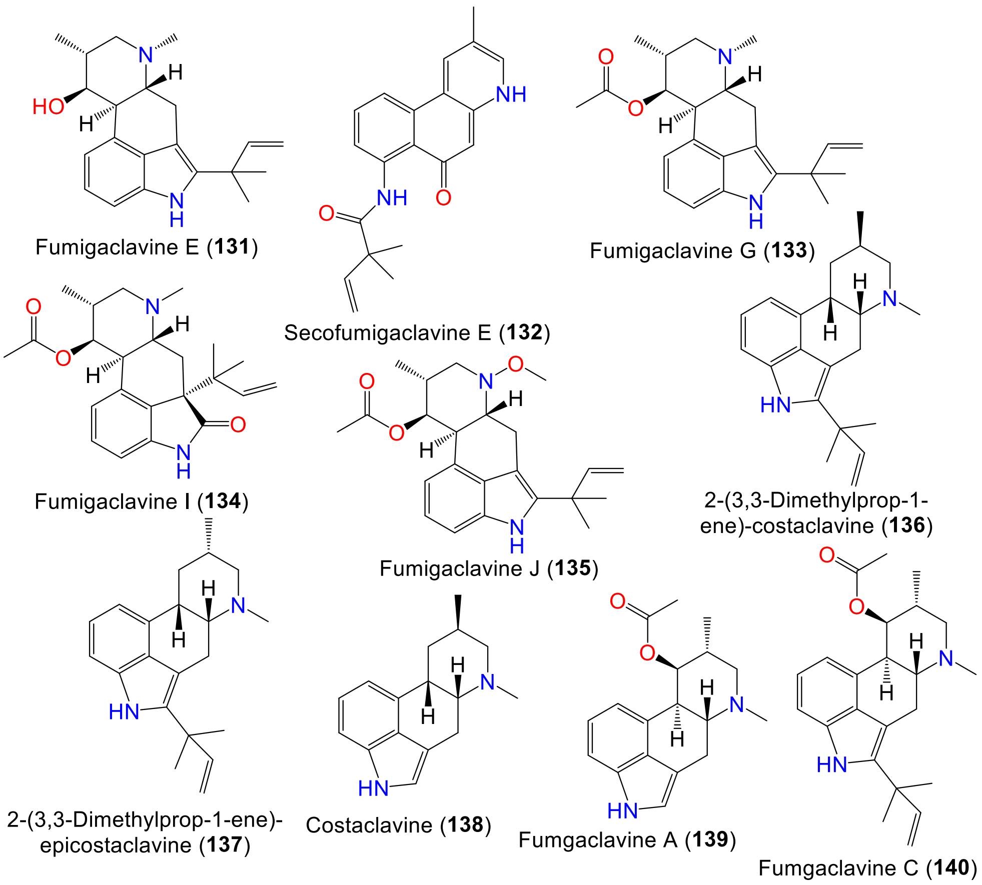

Fig. S11. **Chemical structures of clavine-type ergot alkaloids (131–140)**.

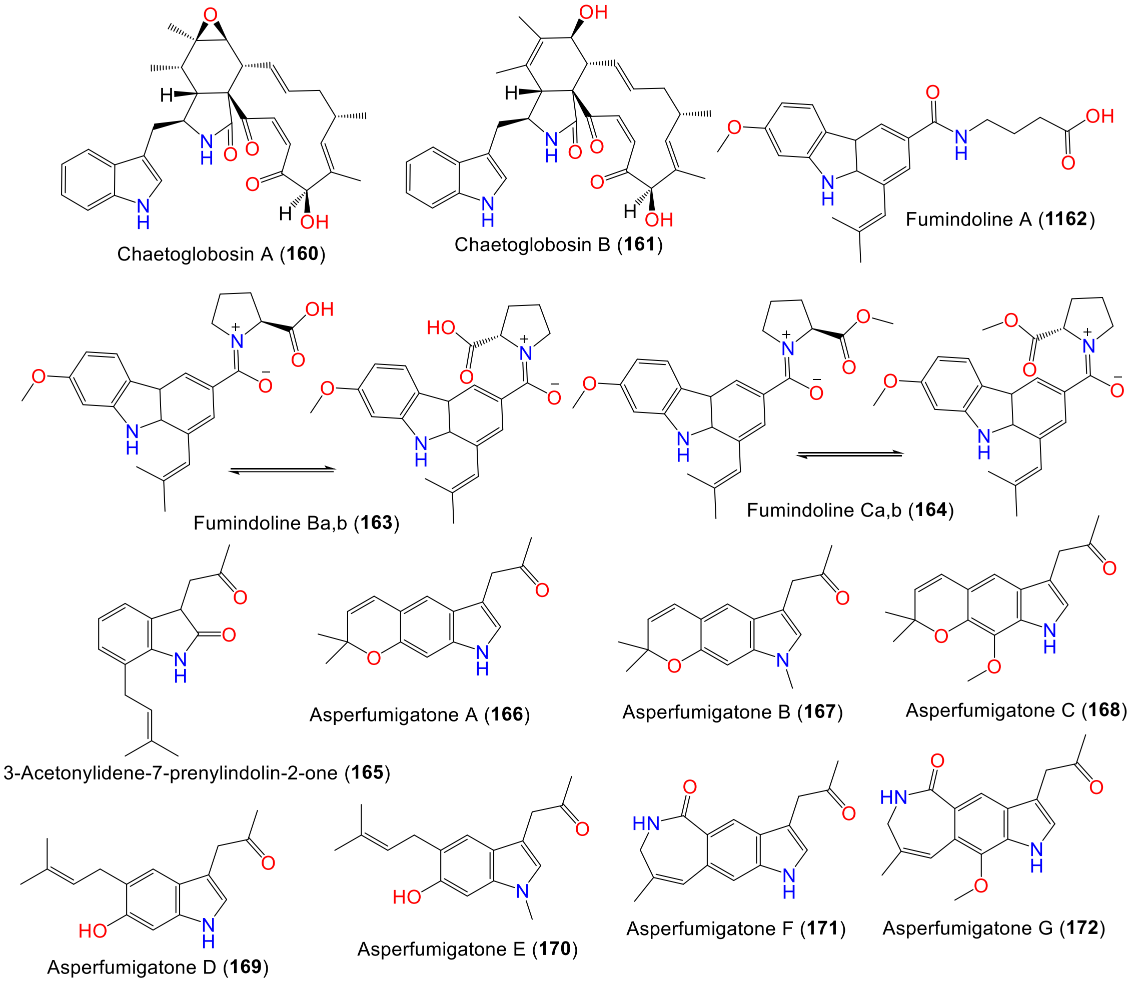

Fig. S12. Chemical structures of indoline (160–165) and indole (166–172) alkaloids.

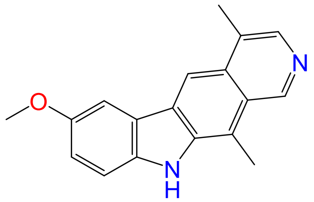

9-Methoxyolivacine (**173**)

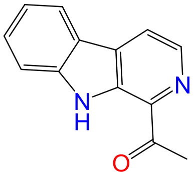

1-Acetyl-β-carboline (**174**)

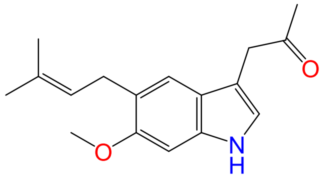

1-(6-Methoxy-5-prenyl-1H-indol-3-yl)-propan-2-one (**175**)

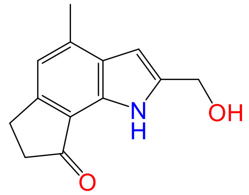

Dilemmaone B (**176**)

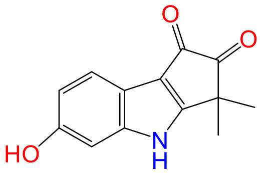

Bruceolline H (**177**)

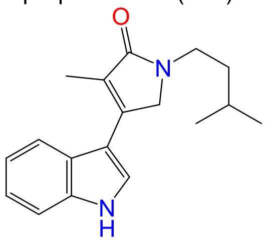

Psammocindole B (**178**)

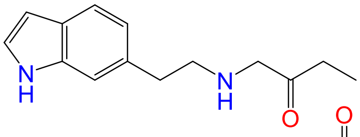

N-Acetyltyramine (**179**)

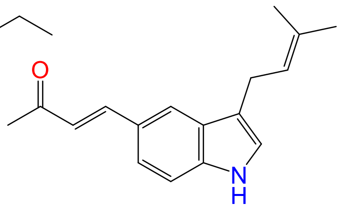

3-Prenyl-5-(3-keto-but-1-enyl)-indole (**180**)

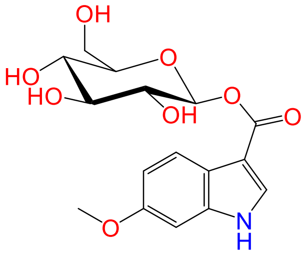

6-Methoxyindole-3-carboxylic acid *O*-β-D-glucopyranosyl ester (**181**)

Fig. S13. **Chemical structures of indole alkaloids (173–181)**.

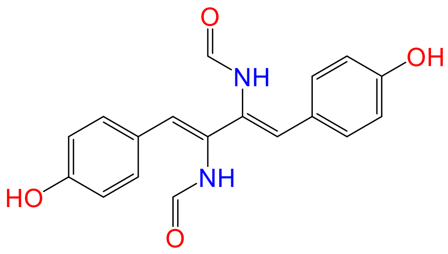

N,N`-((1*Z*,3*Z*)-1,4-Bis(4-hydroxyphenyl)buta-1,3-diene-2,3-diyl)diformamide (**182**)

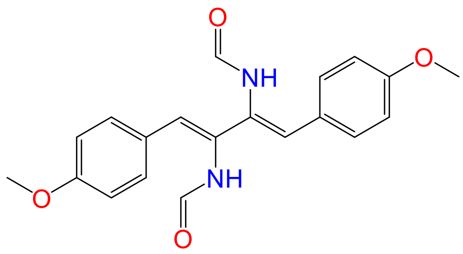

N,N`- ((1*Z*,3*Z*)-1,4-Bis(4-methoxyphenyl)buta-1,3-diene-2,3-diyl)diformamide (**183**)

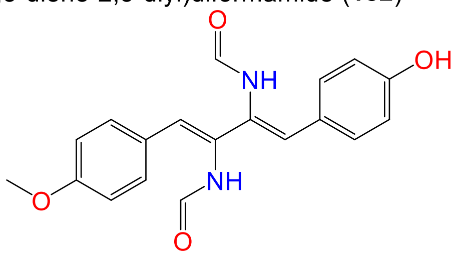

N,N`-((1*Z*,3*Z*)-1-(4-Hydroxyphenyl)-4-(4-methoxyphenyl) buta-1,3-diene-2,3-diyl)diformamide (**184**)

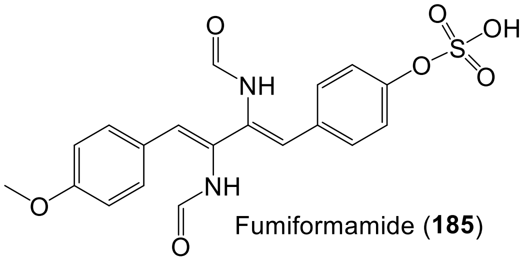

Fumiformamide (**185**)

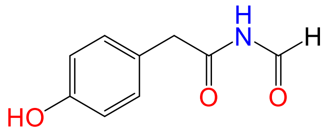

N-Formyl-4-hydroxyphenyl-acetamide (**186**)

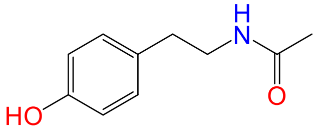

N-Acetyltyramine (**187**)

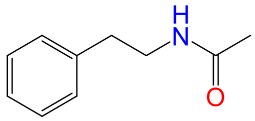

N-(2-Phenylethyl)acetamide (**188**)

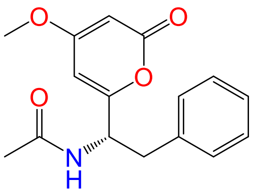

Pyrophen (**189**)

Fig. S14. Chemical structures of amide-containing alkaloids (182–189).

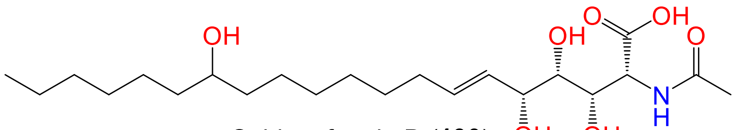

Sphingofungin D (**190**)

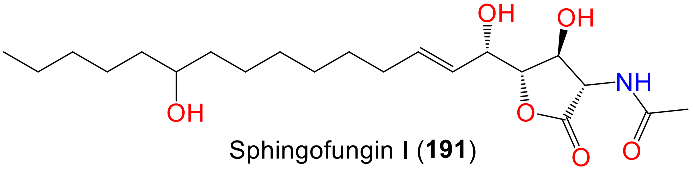

Sphingofungin I (**191**)

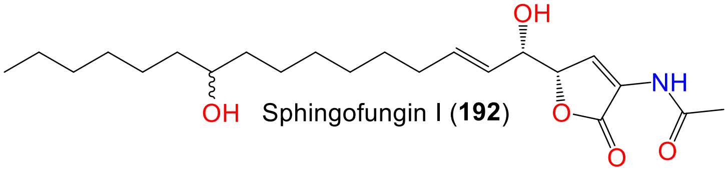

Sphingofungin I (**192**)

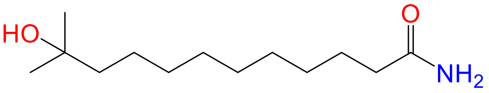

11-Methyl-11-hydroxyldodecanoic acid amide (**193**)

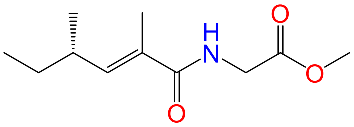

(*S*, *E*)-Methyl-2-(2,4-dimethylhex-2-enamido)acetate (**194**)

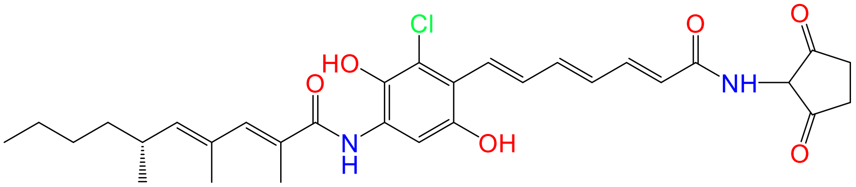

2,5-Dioxocyclopentylamino-7-oxohepta-1,3,5-trienyl-2,5-dihydroxy-3-chlorophenyl-2,4,6-trimethyldeca-2,4-dienamide (**195**)

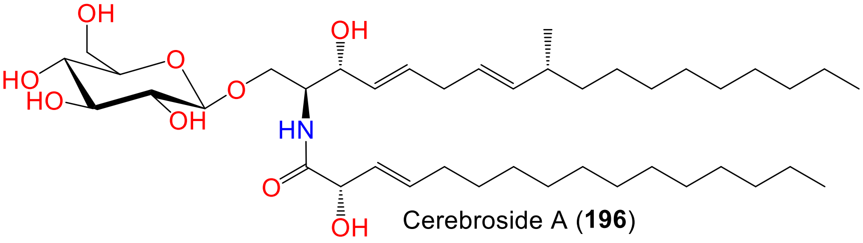

Cerebroside A (**196**)

Fig. S15. Chemical structures of amide-containing alkaloids (190–196).

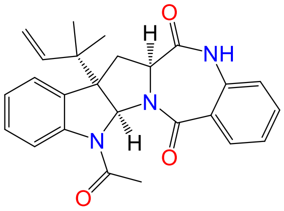

epi-Aszonalenin A (**209**)

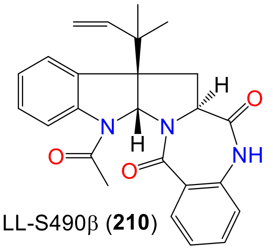

LL-S490β (**210**)

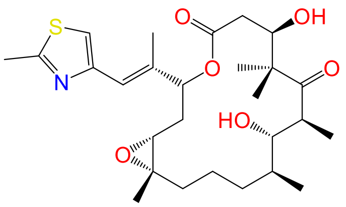

Epothilone B (**211**)

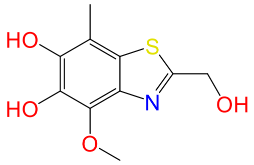

2-Hydroxymethyl-4-methoxy-7-methylbenzo[d]thiazole-5,6-diol (**212**)

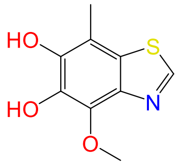

4-Methoxy-7-methylbenzo[*d*]thiazole-5,6-diol (**213**)

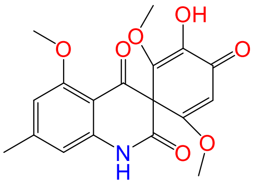

Asperfumoid (**214**)

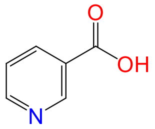

Nicotinic acid (**215**)

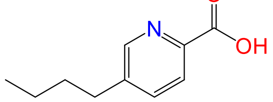

5-Butyl-2-pyridine carboxylic acid (**216**)

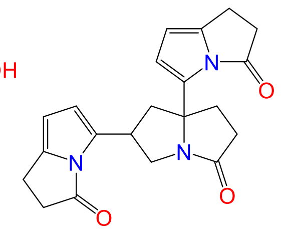

Pyrazoline-3-one trimer (**217**)

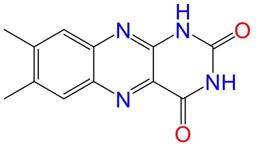

Lumichrome (**218**)

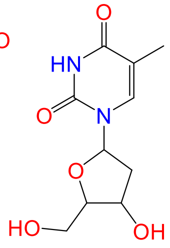

2`-Deoxy-thymidine (**219**)

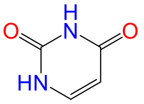

Uracil (**220**)

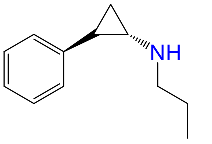

(1*S*,2*R*)-2-phenyl-N-propylcyclopropanamine (**221**)

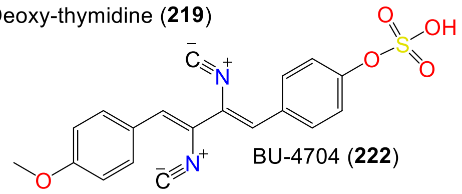

BU-4704 (**222**)

Fig. S16. **Chemical structures of other alkaloids (209–222)**.

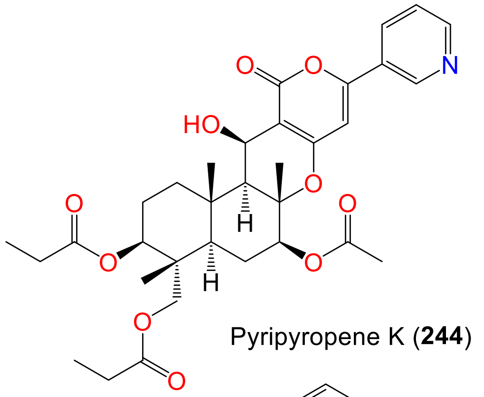

Pyripyropene K (**244**)

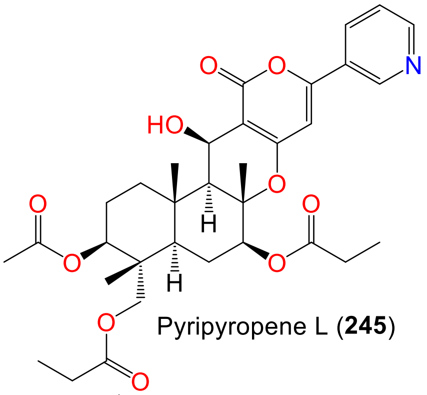

Pyripyropene L (**245**)

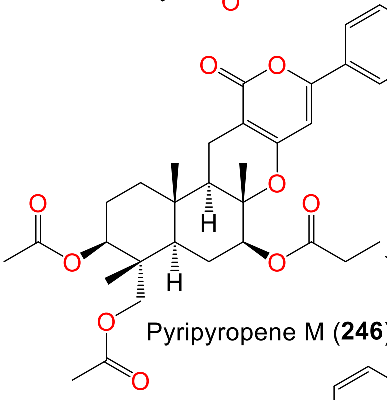

Pyripyropene M (**246**)

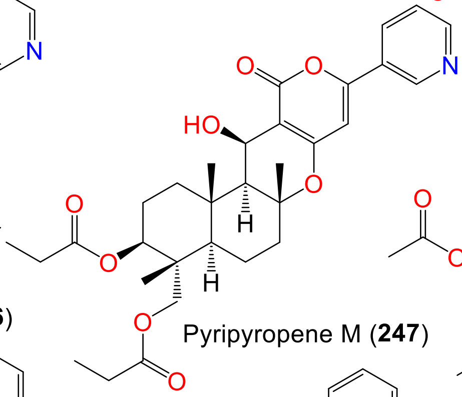

Pyripyropene M (**247**)

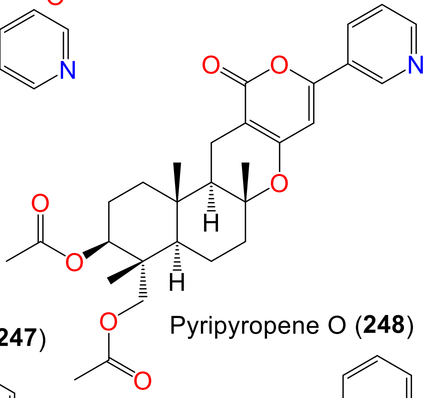

Pyripyropene O (**248**)

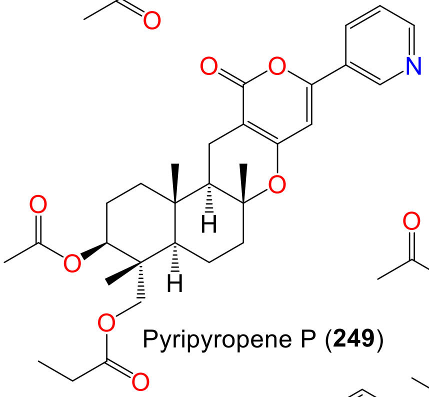

Pyripyropene P (**249**)

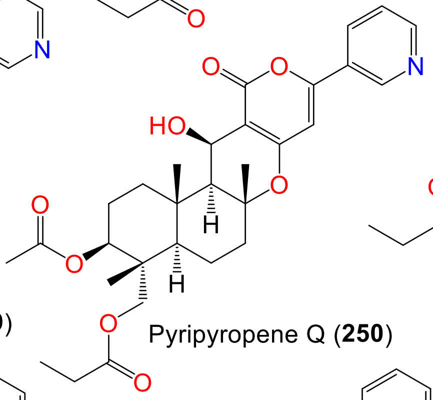

Pyripyropene Q (**250**)

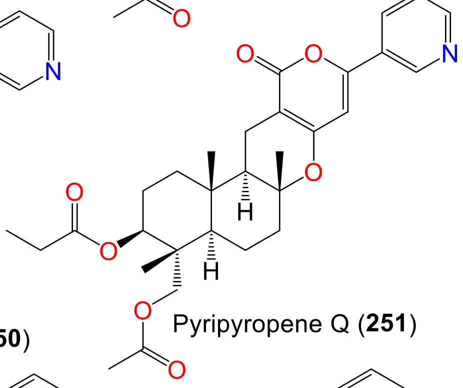

Pyripyropene Q (**251**)

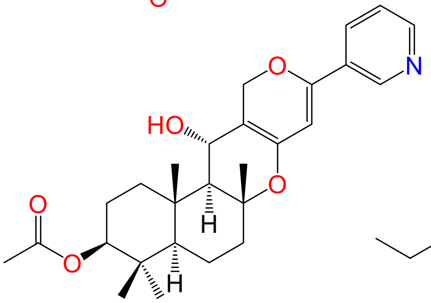

GERI-BP001 A (**252**)

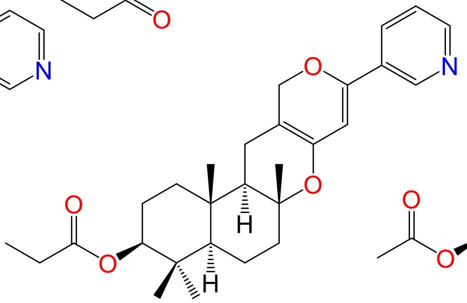

GERI-BP001 B (**253**)

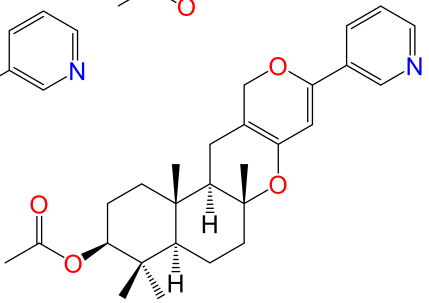

GERI-BP001 M (**254**)

Fig. S17. **Chemical structures of pyridino-α-pyrone sesquiterpenoids** (**244–254**).

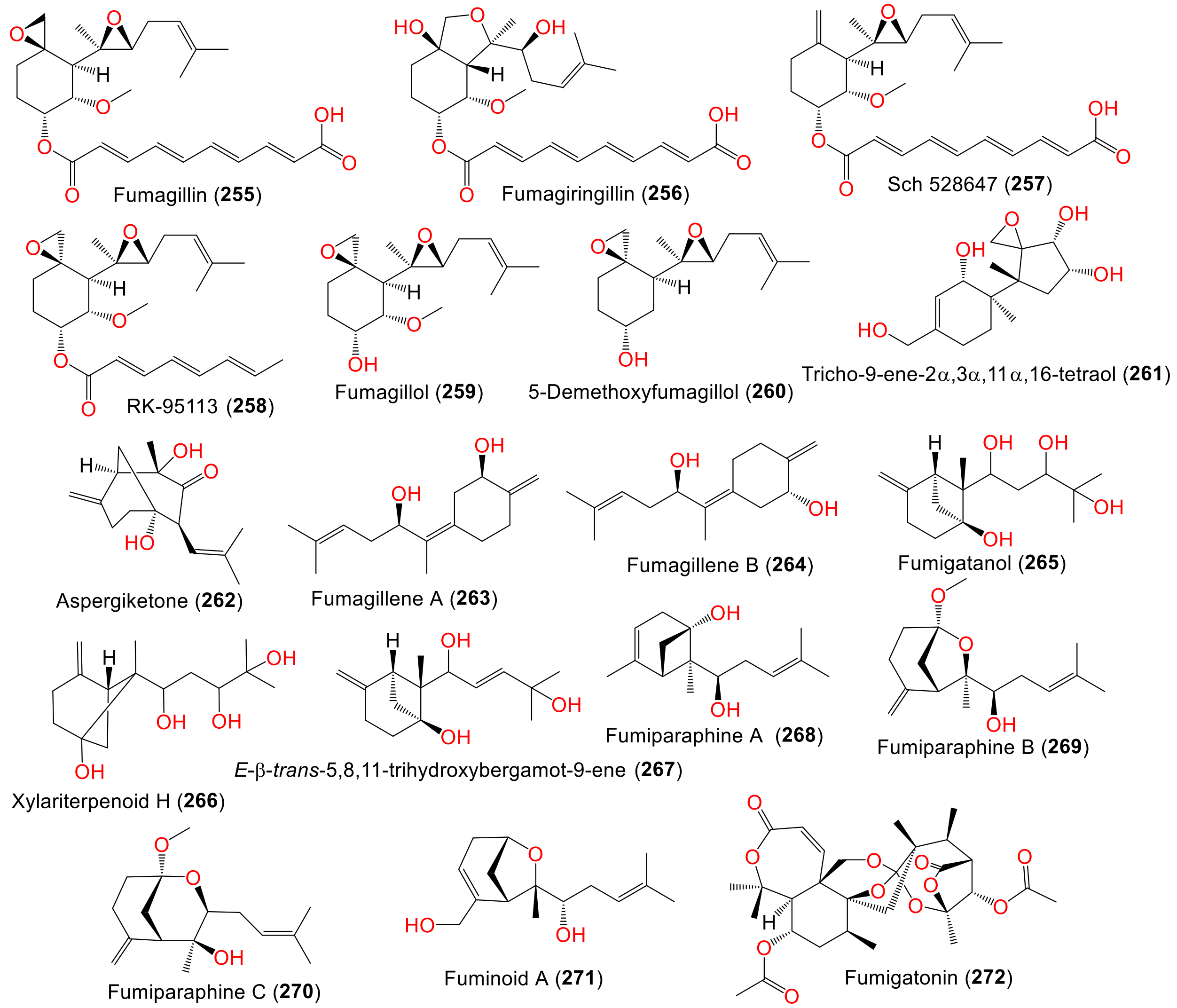

Fig. S18. Chemical structures of sesquiterpenoids (255–271) and meroterpenoid (272).

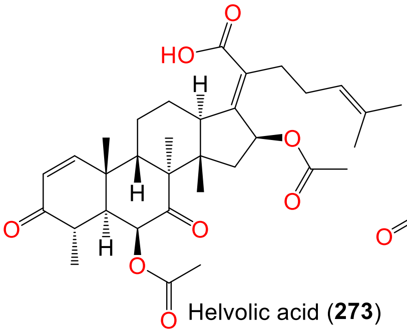

Helvolic acid (**273**)

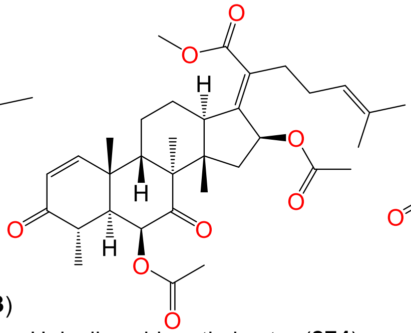

Helvolic acid methyl ester (**274**)

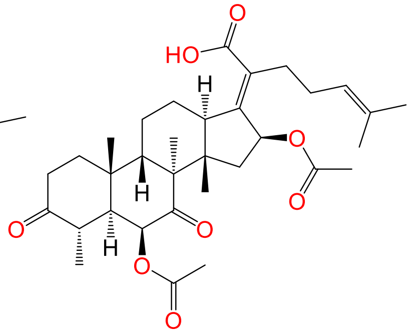

1,2-Dihydrohelvolic acid (**275**)

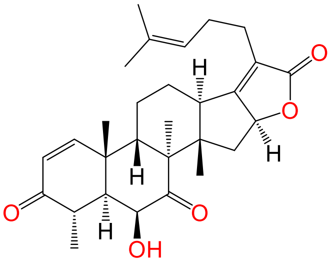

6,16-O-Dideacetylhelvolic acid 21,16-lactone (**276**)

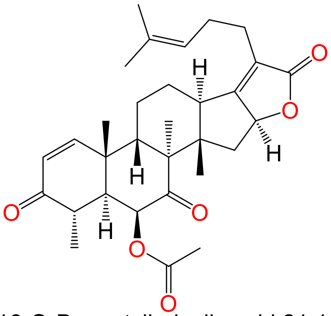

16-O-Deacetylhelvolic acid 21,16-lactone (**277**)

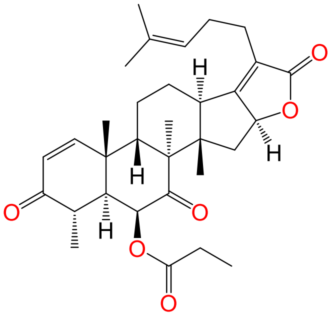

6-O-Propionyl-6,16-O-dideacetylhelvolic acid 21,16-lactone (**278**)

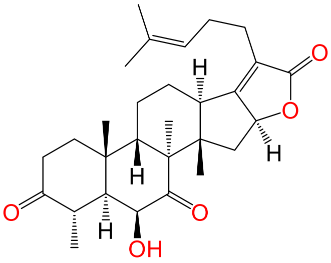

1,2-Dihydro-6,16-O-dideacetylhelvolic acid 21,16-lactone (**279**)

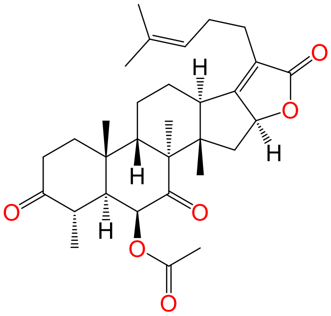

1,2-Dihydro-16-O-deacetylhelvolic acid 21,16-lactone (**280**)

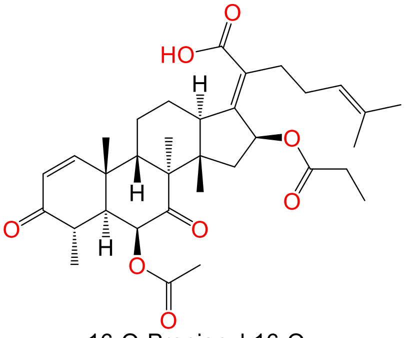

16-O-Propionyl-16-O-deacetylhelvolic acid (**281**)

Fig. S19. Chemical structures of triterpenoids (273-281).

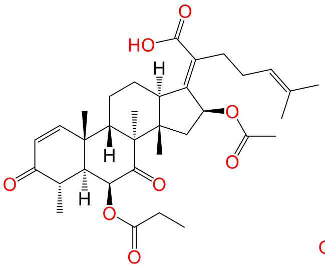

6-O-Propionyl-6-O-deacetylhelvolic acid (**282**)

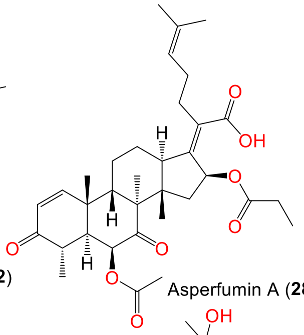

Asperfumin A (**283**)

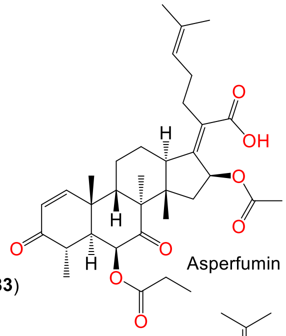

Asperfumin B (**284**)

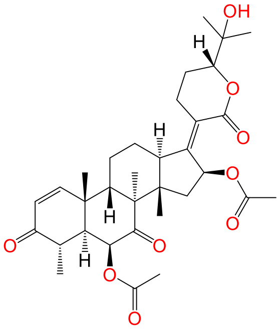

6β,16β-Diacetoxy-25-hydroxy-3,7-dioxo-29-nordammara-1,17(20)-dien-21,24-lactone (**285**)

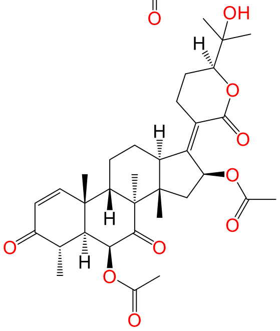

24-*epi*-6β,16β-Diacetoxy-25-hydroxy-3,7-dioxo-29-nordammara-1,17(20)-diene-21,24-lactone (**286**)

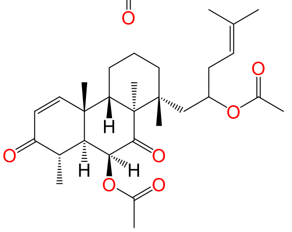

4,8,10,14-Tetramethyl-6-acetoxy-14-[16-acetoxy-19-(20,21-dimethyl)-18-ene]-phenanthrene-1-ene-3,7-dione (**287**)

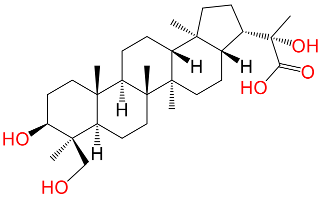

(*S*)-2-hydroxy-2-((3*S*,3a*S*,5a*R*,5b*R*,7a*R*,8*S*,9*S*,11a*R*,11b*R*,13a*R*,13b*S*)-9-Hydroxy-8-(hydroxymethyl)-5a,5b,8,11a,13b-pentamethylicosahydro-1*H*-cyclopenta[*a*]chrysen-3-yl)propanoic acid (**290**)

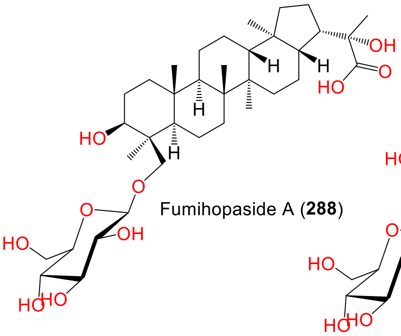

Fumihopaside A (**288**)

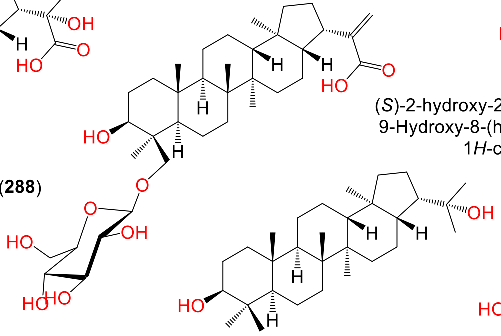

Fumihopaside B (**289**)

21βH-hopane-3β,22-diol (**291**)

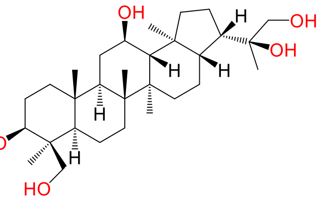

1,4,23-Trihydroxy-hopane-22,30-diol (**292**)

Fig. S20. **Chemical structures of triterpenoids (282–292)**.

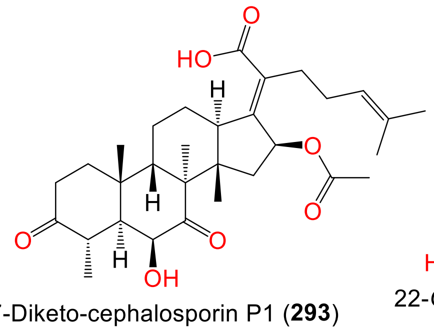

3,7-Diketo-cephalosporin P1 (**293**)

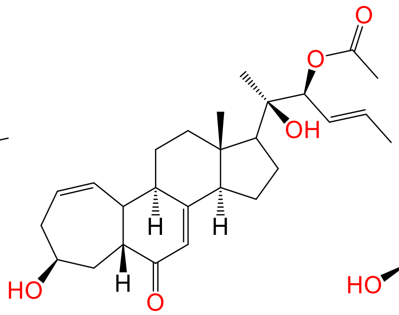

22-*O*-Acetylisocyclocitrinol A (**294**)

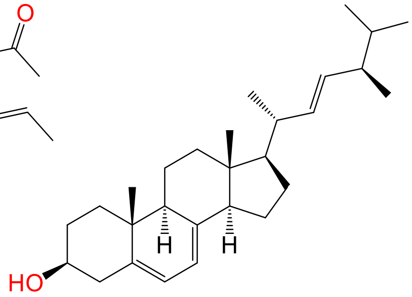

Ergosterol (**295**)

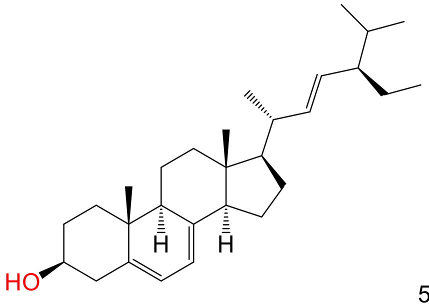

(3β,22*E*,24*R*)-Stigmasta-5,7,22-trien-3-β-ol (**296**)

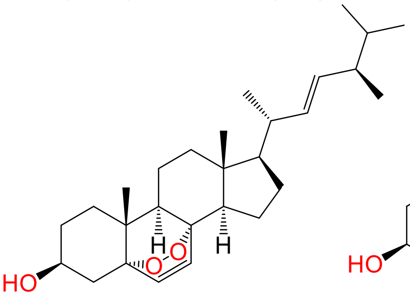

5α,8α-Epidioxy-ergosta-6,22-diene-3β-ol (**297**)

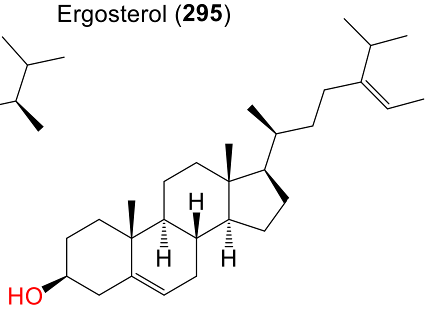

Isofucosterol (**298**)

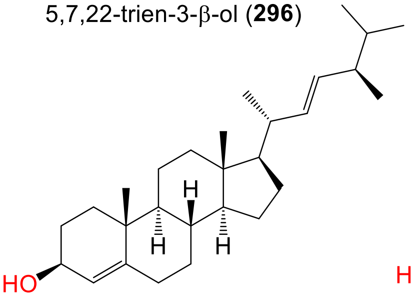

Ergosta-4,22-diene-3β-ol (**299**)

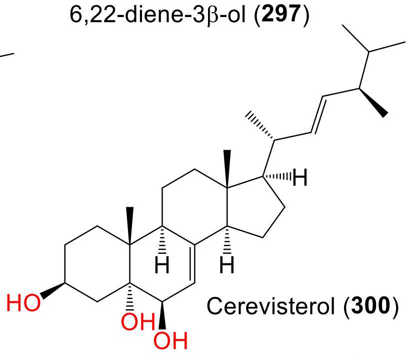

Cerevisterol (**300**)

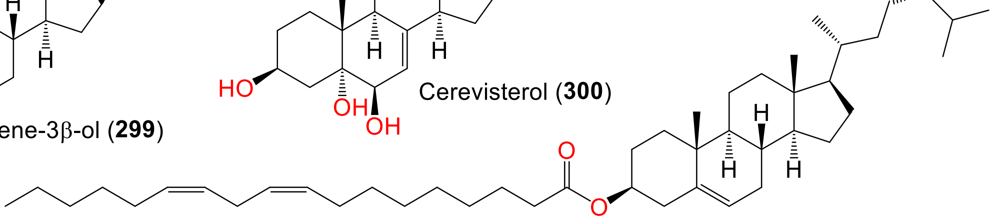

β-Sistosterol linoleate (**301**)

Fig. S21. Chemical structures of sterols (293-301).

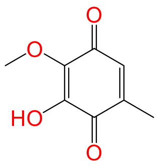
Fumigatin (**302**)

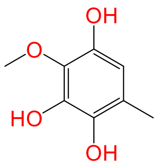
Fumigatin quinol (**303**)

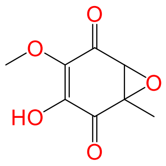
Fumigatin oxide (**304**)

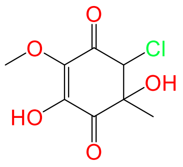
Fumigatin chlorohydrin (**305**)

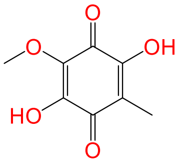
Spinulosin (**306**)

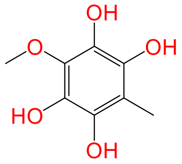
Spinulosin quinol (**307**)

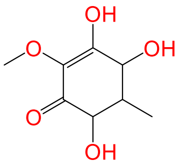
Dihydrospinulosin quinol (**308**)

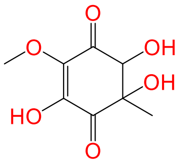
Spinulosin hydrate (**309**)

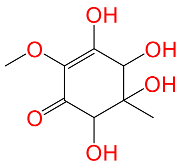
Spinulosin quinol-hydrate (**310**)

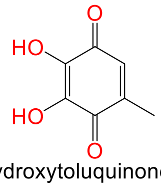
3,4-Dihydroxytoluquinone (**311**)

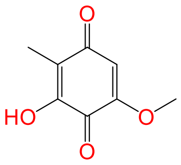
3-Hydroxy-5-methoxy-2-methylbenzoquinone (**312**)

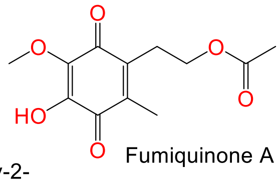
Fumiquinone A (**313**)

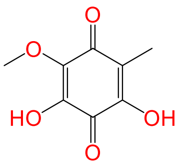
Fumiquinone B (**314**)

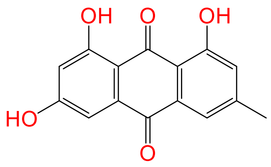
Emodin (**315**)

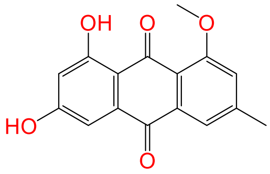
1-Methyl emodin (**316**)

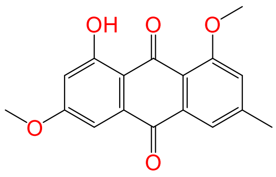
Emodin 1,6-dimethyl ether (**317**)

Fig. S22. Chemical structures of quinones (302-317).

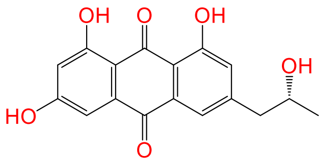

Isorhodoptilometrin (**318**)

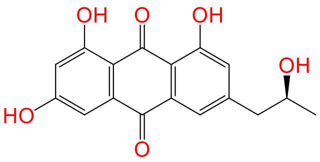

(+)-2`S-Isorhodoptilometrin (**319**)

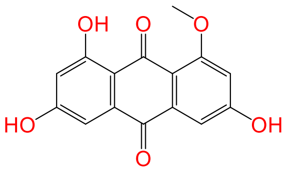

Questin (**320**)

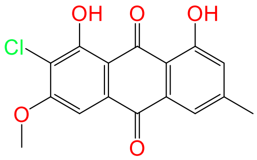

Fragilin (**321**)

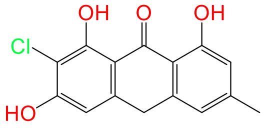

2-Chloro-1,3,8-trihydroxy-6-methylanthrone (**322**)

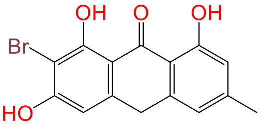

2-Bromo-1,3,8-trihydroxy-6-methylanthrone (**323**)

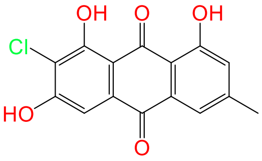

2-Chloro-1,3,8-trihydroxy-6-methylanthraquinone (**324**)

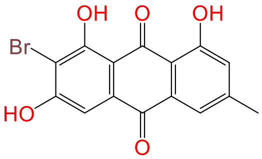

2-Bromo-1,3,8-trihydroxy-6-methylanthraquinone (**325**)

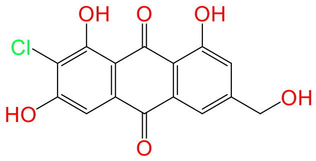

2-Chloro-1,3,8-trihydroxy-6-hydroxymethylanthraquinone (**326**)

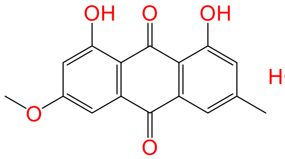

Physcion (**327**)

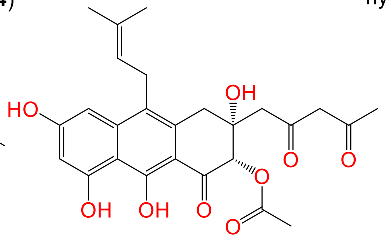

Neosartoricin (**328**)

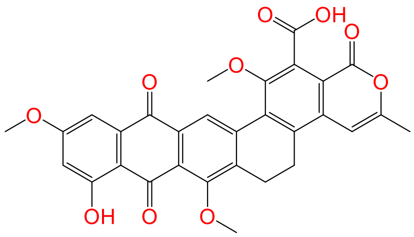

2,4,5,17-Tetramethoxy pradimicin lactone (**329**)

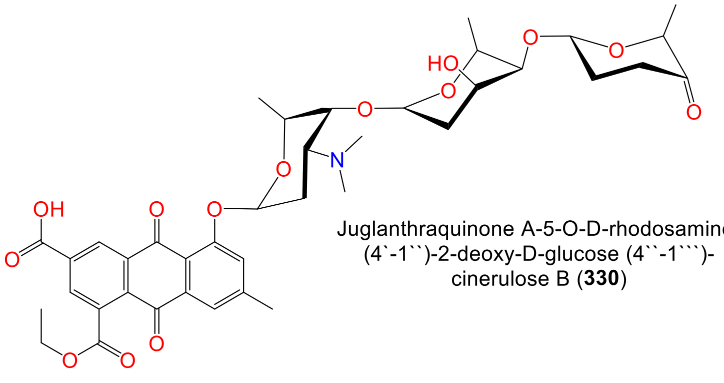

Juglanthraquinone A-5-O-D-rhodosamine-(4`-1``)-2-deoxy-D-glucose (4``-1```)-cinerulose B (**330**)

Fig. S23. Chemical structures of quinone derivatives (**318-330**).

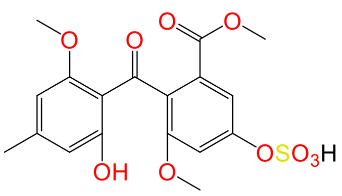

Monomethylsulochrin-4-sulphate (**331**)

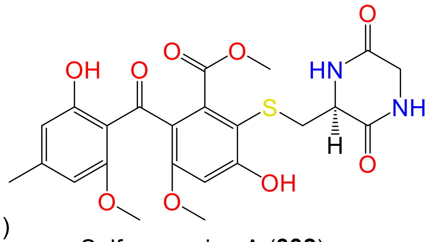

Sulfurasperine A (**332**)

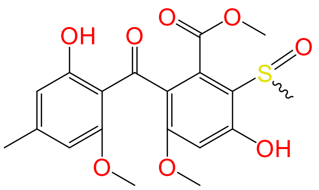

Sulfurasperine B ((±) (**333**)

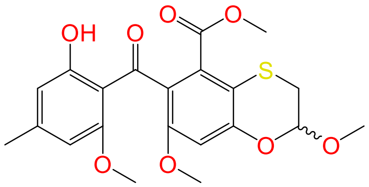

Sulfurasperine C ((±) (**334**)

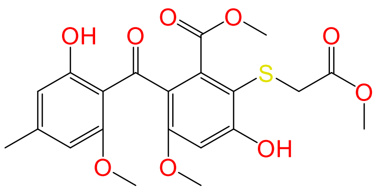

Sulfurasperine D (**335**)

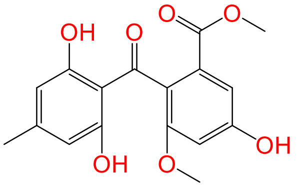

Sulochrin (**336**)

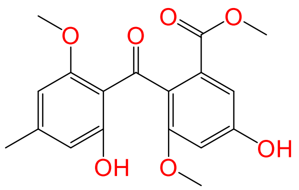

Monomethylsulochrin (**337**)

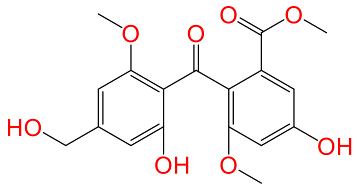

8`-Hydroxymonomethylsulochrin (**338**)

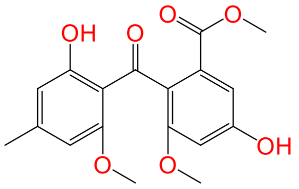

5-Hydroxy-2-(2-hydroxy-6-methoxy-4-methylbenzoyl)-3-methoxybenzoic acid (**339**)

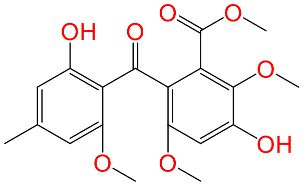

Asperfumin (**340**)

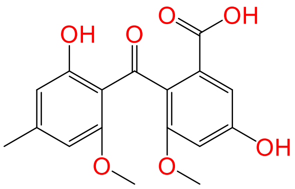

Rhizoctonic acid (**341**)

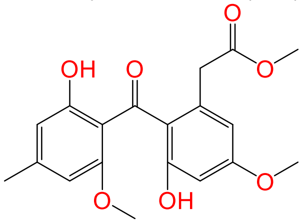

2,6`-Dihydroxy-2,4`-dimethoxy-8`- methyl-6-methoxy-acyl-ethyl-diphenylmethanone (**342**)

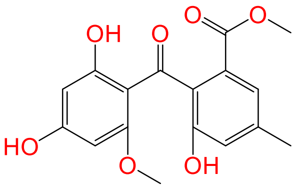

Penibenzophenone E (**343**)

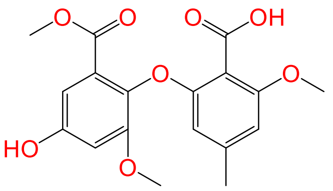

8`-*O*-Methylasterric acid (**344**)

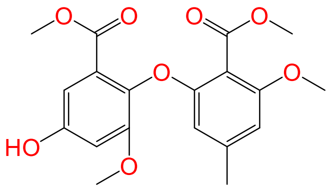

Dimethyl 2,3`-dimethylosoate (**345**)

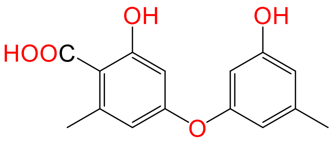

4-Carboxy-5,5`-dihydroxy-3,3`-dimethyl-diphenylether (**346**)

Fig. S24. Chemical structures of benzophenones (331–343) and diphenyl ethers (344–346).

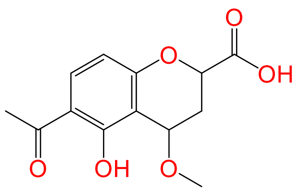

Ruakuric acid (**347**)

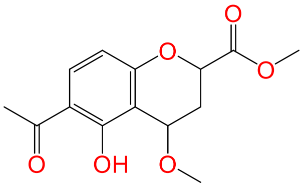

Ruakuric acid methyl ester (**348**)

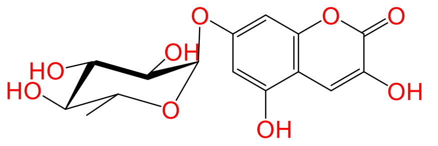

3,5-Dihydroxy-7-O-α-rhamnopyranoyl-2H-chromen-2-one (**349**)

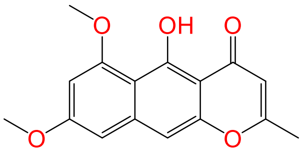

Rubrofusarin B (**350**)

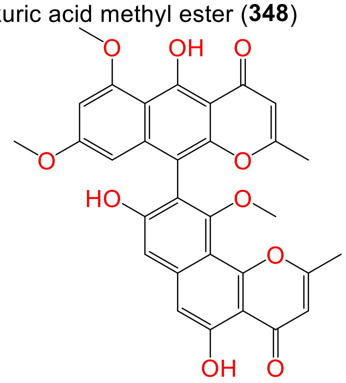

Asperpyrone A (**351**)

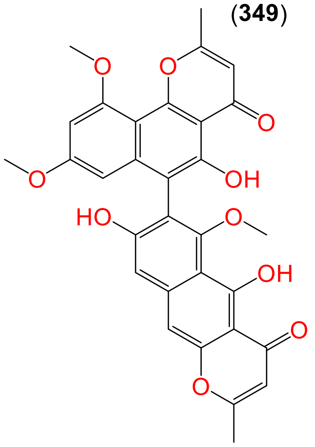

Asperpyrone D (**352**)

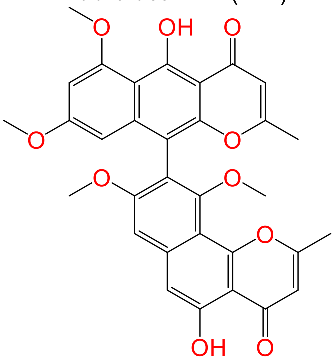

Fonsecinone A (**353**)

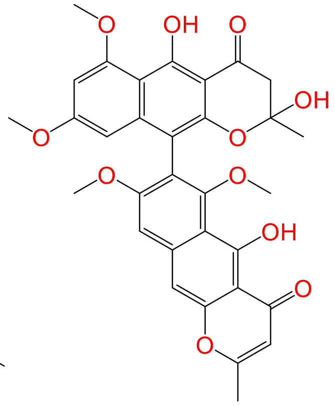

Fonsecinone B (**354**)

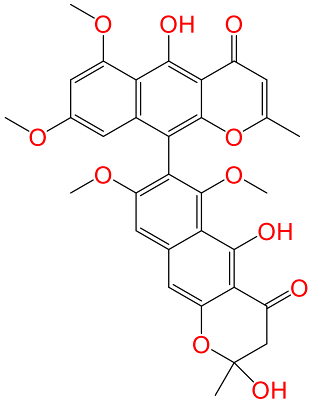

Fonsecinone D (**355**)

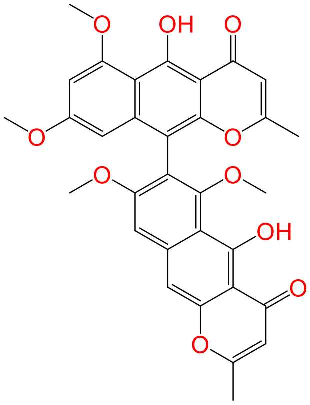

Aurasperone A (**356**)

Fig. S25. **Chemical structures of chromane derivatives (347–356).**

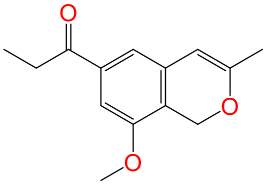

1-(8-Methoxy-3-methyl-1H-isochromen-6-yl)propan-1-one (**357**)

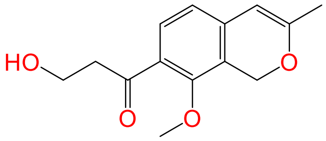

3-Hydroxy-1-(8- methoxy-3-methyl-1H-isochromen-7-yl)propan-1-one (**358**)

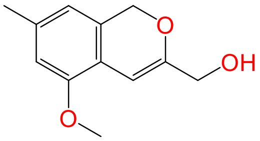

(5-Methoxy-7-methyl-1H-isochromen-3-yl)methanol (**359**)

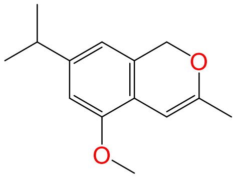

7-Isopropyl-5-methoxy-3-methyl-1H-isochromene (**360**)

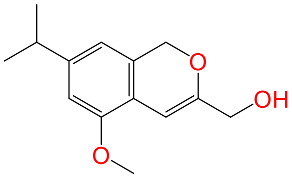

(7-Isopropyl-5-methoxy-1H-isochromen-3-yl)methanol (**361**)

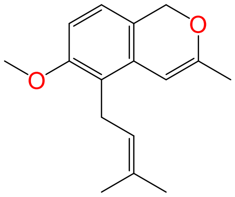

Versicolol B (**362**)

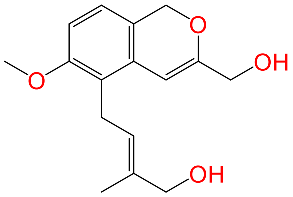

Oryzaein D (**363**)

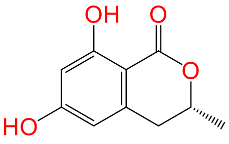

6-Hydroxy mellein (**364**)

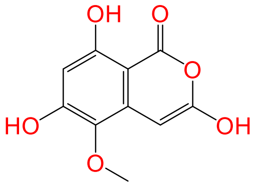

6,8-dihydroxy-5-methoxy-3-methyl-1H-isochromen-1-one (**365**)

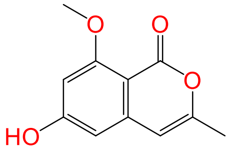

6-Hydroxy-8-methoxy-3-methylisocoumarin (**366**)

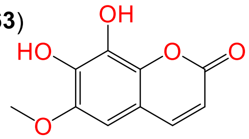

Fraxetin (**367**)

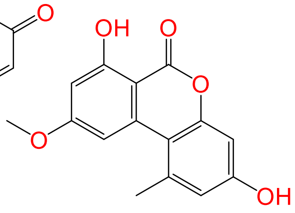

Alternariol 9-O-methyl ether (**368**)

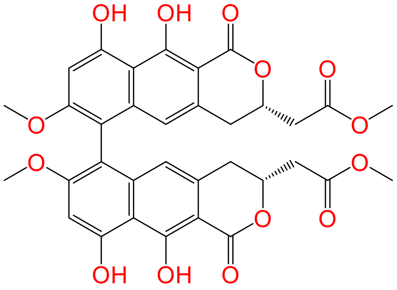

(-)-Viriditoxin (**369**)

Fig. S26. **Chemical structures of isochromane derivatives (357–369).**

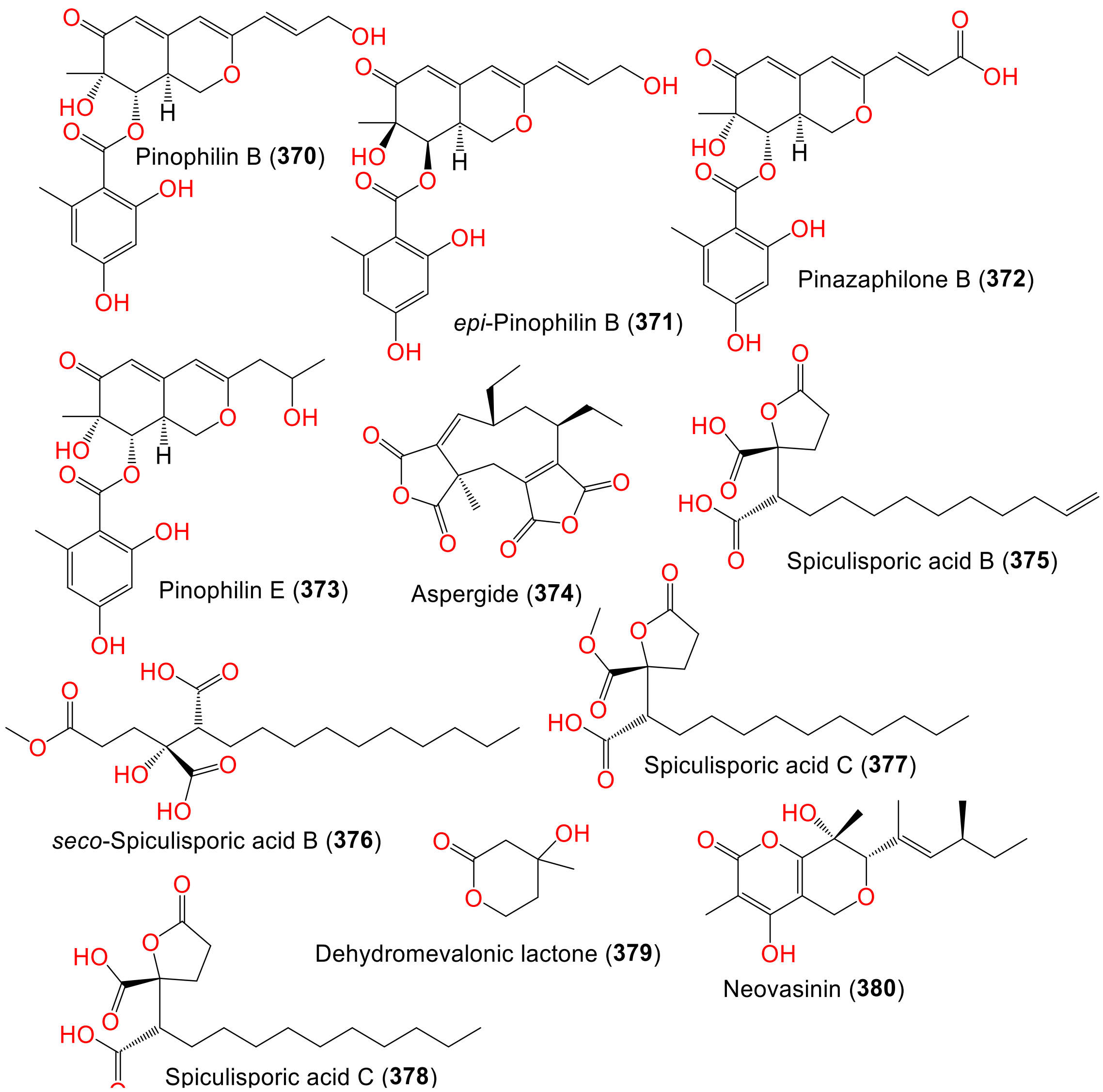

Fig. S27. Chemical structures of azophilane (370–373), anhydride (374–378), and pyranones (379 and 380).

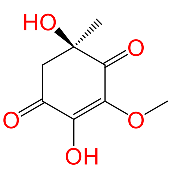

Asperfumtone A (**381**)

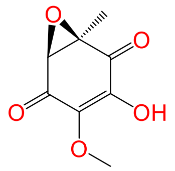

Asperfumtone B (**382**)

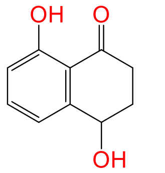

4,8-Dihydroxy-1-tetralone (**383**)

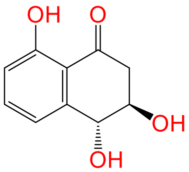

Trans-3,4-Dihydro-3,4,8-trihydroxynaphthalen-1(2H)-one (**384**)

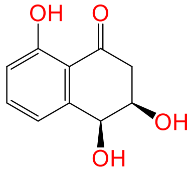

cis-3,4-Dihydro-3,4,8-trihydroxynaphthalen-1(2H)-one (**385**)

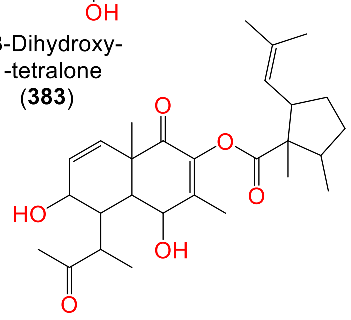

4,6-Dihydroxy, 3,8a-dimethyl-1-oxo-5-(3`-oxobutan-2`-yl)-1,4,4a,5,6,8a-hexahydronaphthalen-2-yl-1``,2``-dimethyl-5``-(2```-methylprop-1```-enyl)cyclopentanecarboxylate (**386**)

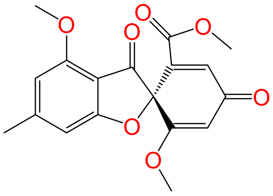

Trypacidin (**387**)

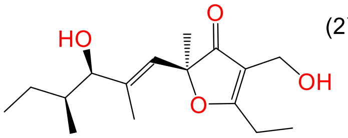

Neovasifuranone B (**388**)

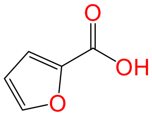

2-Furoic acid (**389**)

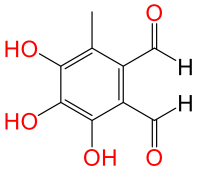

Flavipin (**390**)

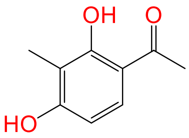

2,4-Dihydroxy-3-methyl-acetophenone (**391**)

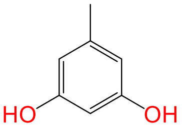

Orcinol (**392**)

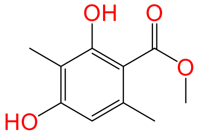

Atraric acid (**393**)

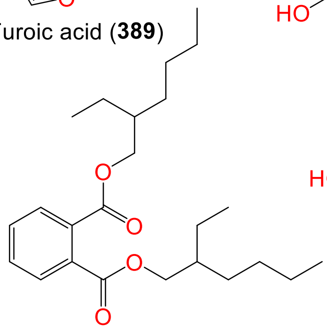

Di-(2-Ethylhexyl) phthalate (**394**)

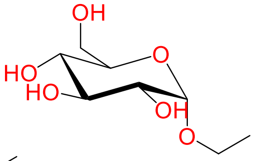

α–Ethyl glucoside (**395**)

Fig. S28. Chemical structures of phenolics (381-395).

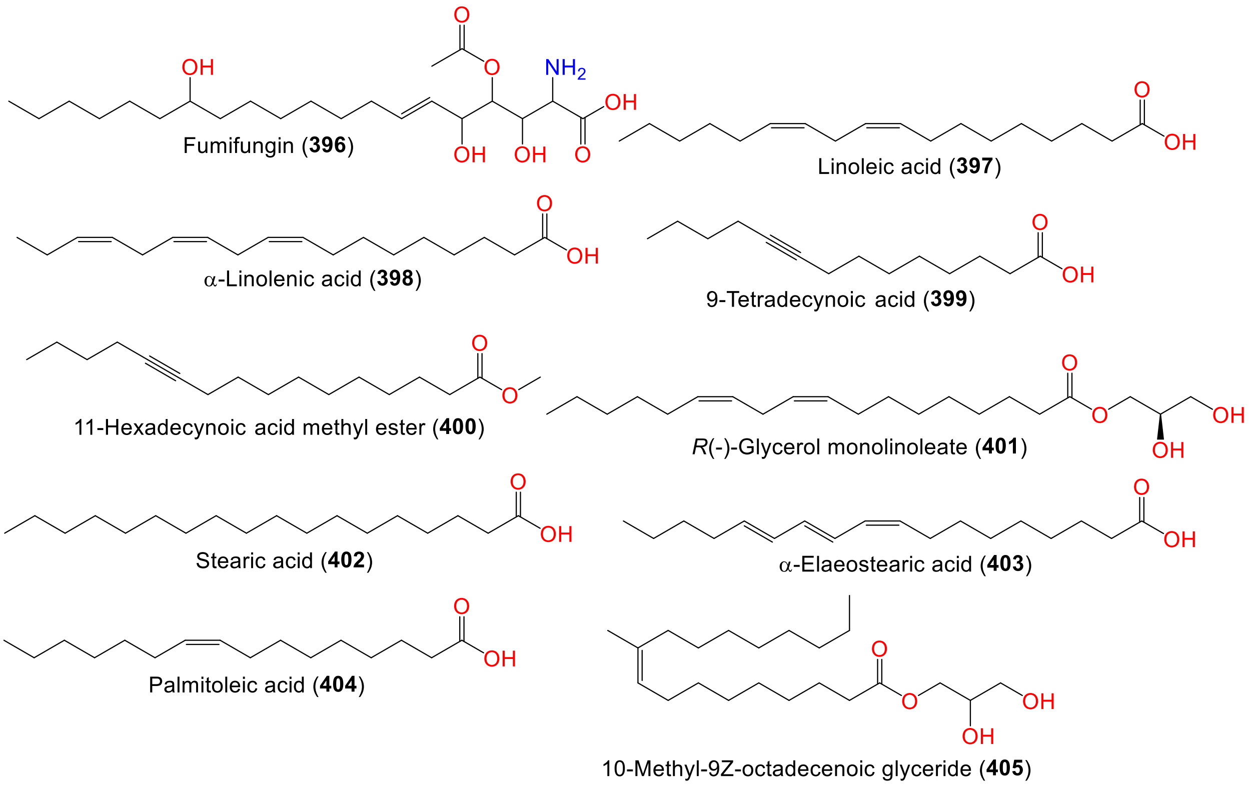

Fig. S29. Chemical structures of fatty acids and related derivatives (396–405).

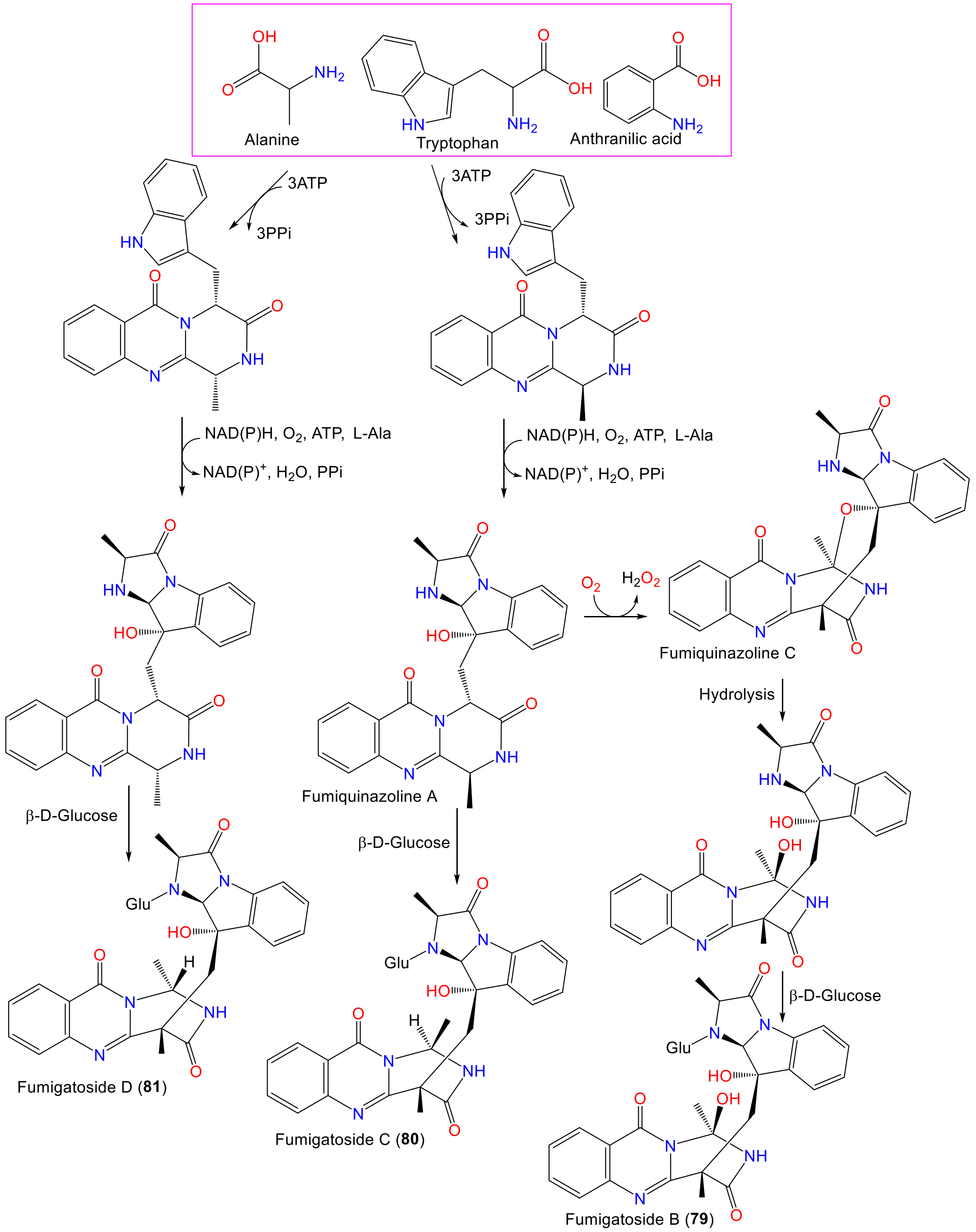

Scheme S1. Biosynthetic pathway of compounds 79–81 [74].

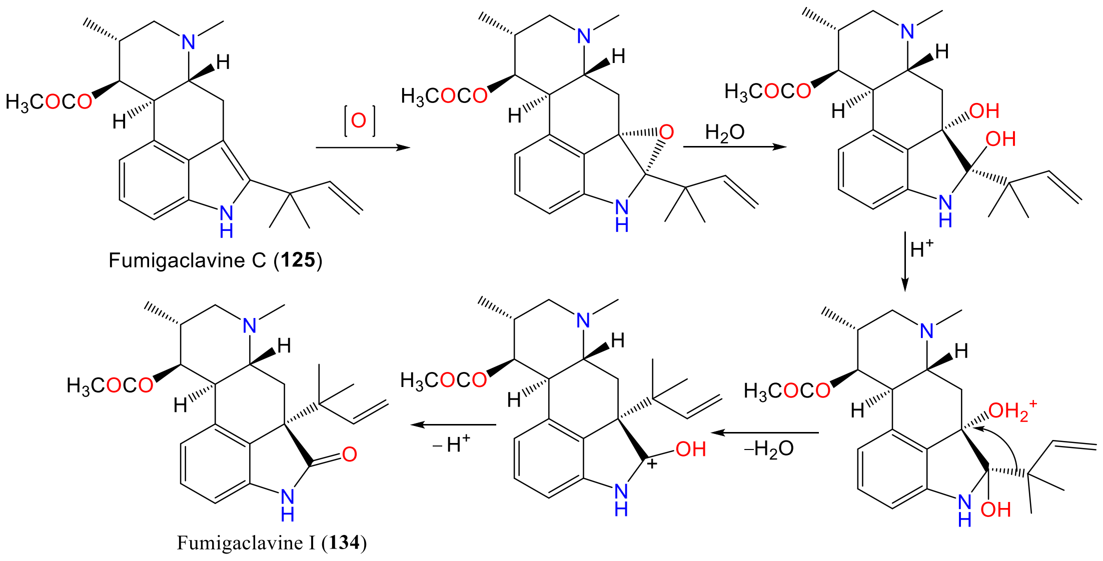

Scheme S2. Proposed biosynthesis of compound **134** from **125** [55].

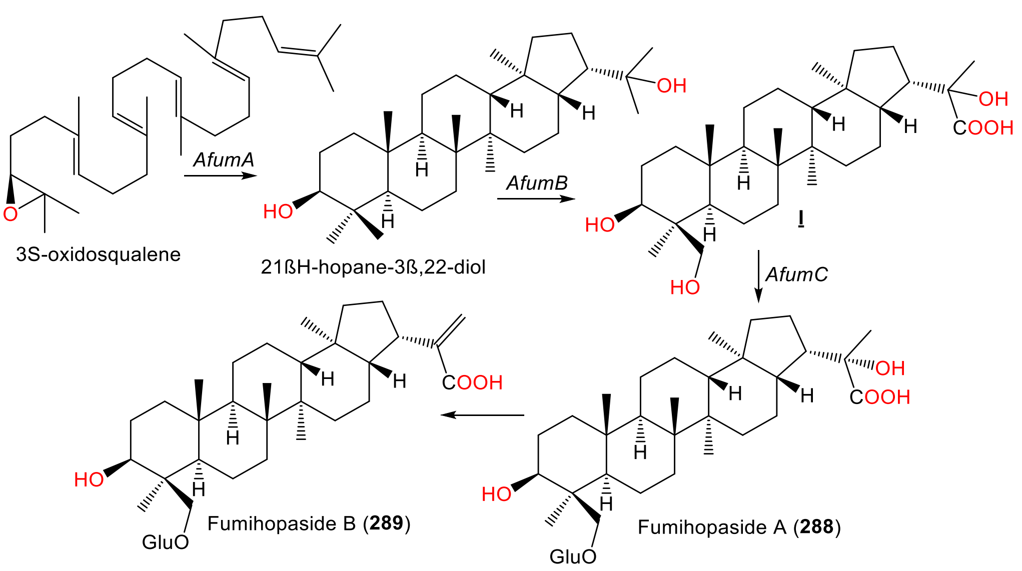

Scheme S3. Biosynthetic pathway of compounds 288 and 289 [131].

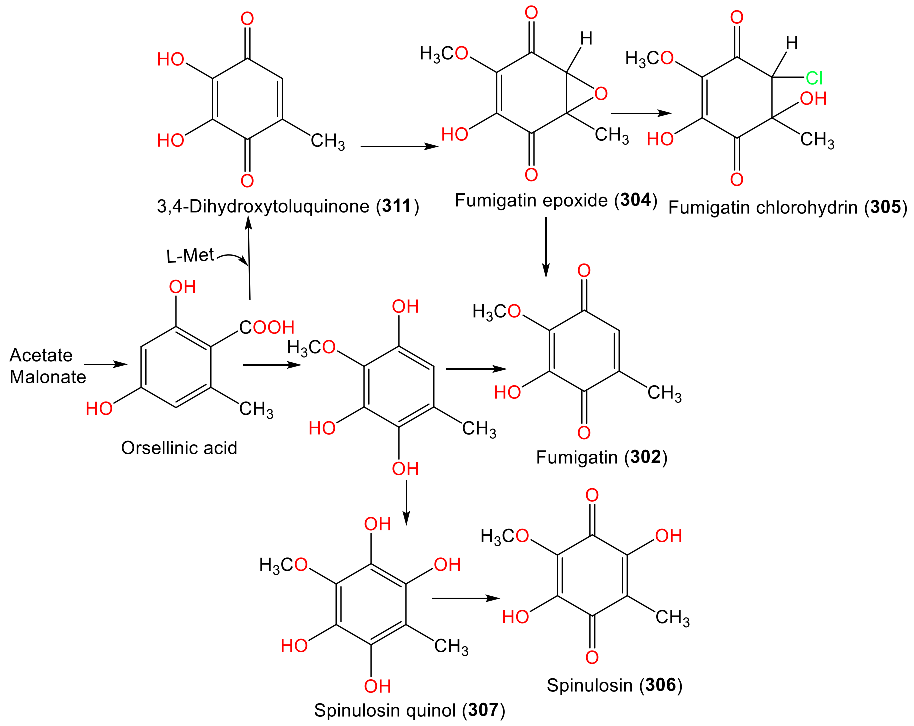

Scheme S4. **Biosynthetic pathway of quinones from orsellinic acid [133,135,136].**

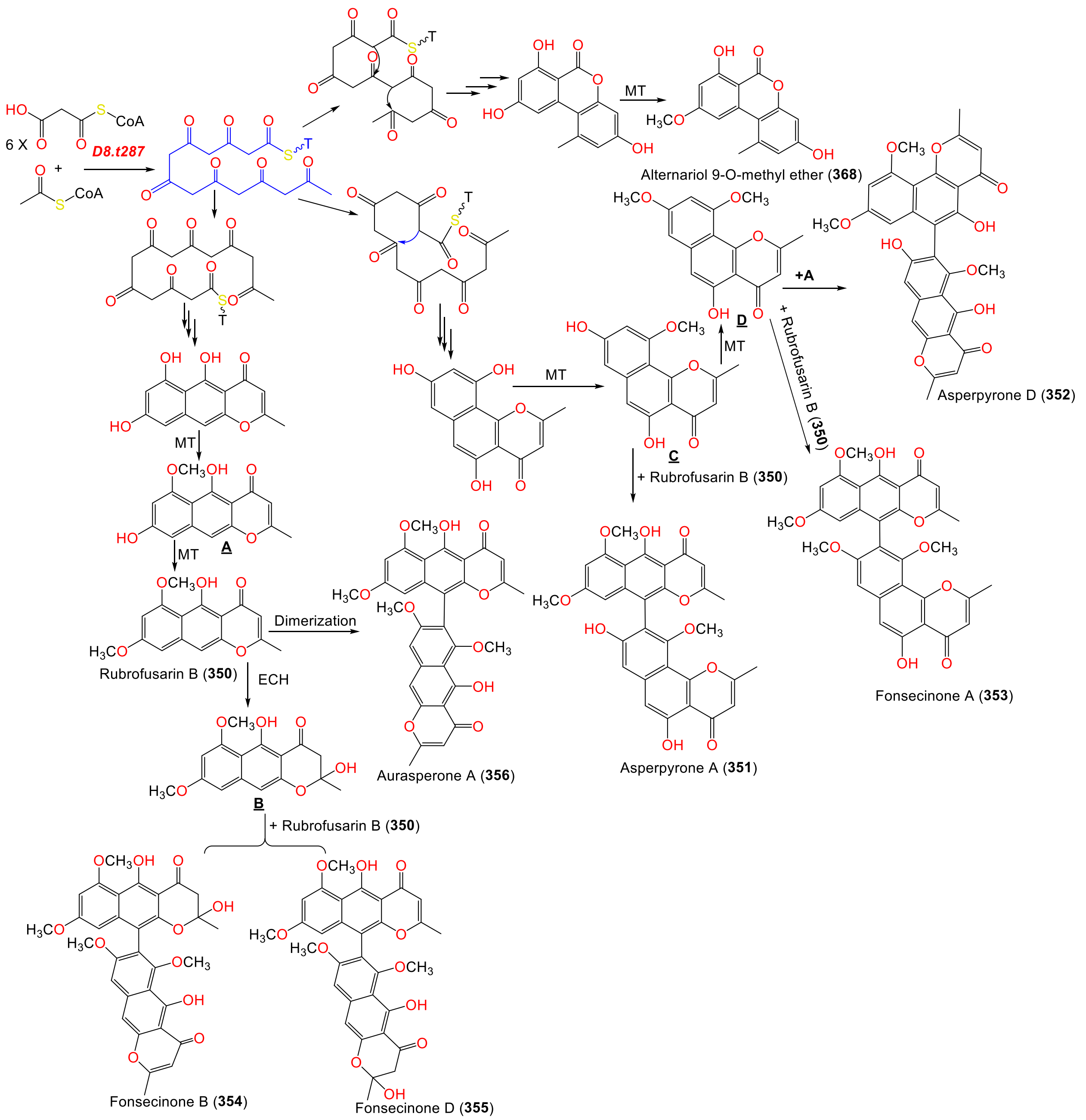

Scheme S5. Biosynthetic pathway of 350–356 and **368** [140].

Table S1. **List of indole-diketopiperazine and indole-quinazoline alkaloids isolated from *Aspergillus fumigatus.***

Compound Name/Chemical Class	M. Wt.	Mol. Formula	Strain, Host, and Location	Ref.
**Indole diketopiperazine**				
Fumitremorgin A (**1**)	579	$C_{32}H_{41}N_3O_7$	*A. fumigatus* Fres., cultured, Japan	[26]
Secofumitremorgin A (**2**)	407	$C_{23}H_{25}N_3O_4$	*A. fumigatus* SD-406, cultured, deep-sea sediment, East China Sea, China	[29]
Fumitremorgin B (**3**)	479	$C_{27}H_{33}N_3O_5$	*A. fumigatus* Fres., cultured, Japan	[26]
	-	-	*A. fumigatus* BM939, cultured, sea sediment sample, mouth of Oi River, Sizuoka prefecture, Japan	[30]
	-	-	*A. fumigatus* KMM 4631, cultured, marine isolate, Russia	[31]
	-	-	*A. fumigatus* Fres., cultured, *Stichopus japonicus* (marine holothurian), Lingshan Island, Qingdao, China	[32]
	-	-	*A. fumigatus* YK-7, sea mud, intertidal zone, Yingkou, China	[33]
	-	-	*A. fumigatus* LN-4, *Melia azedarach* (stem bark, Meliaceae), Yangling, Shaanxi province, China	[34]
	-	-	*A. fumigatus* MBC-F1-10/ *Streptomyces bullii* co-culture, hyper-arid Atacama Desert soil, Chile	[35]
	-	-	*A. fumigatus* AR05, *Astragalus membranaceus* (root), Hengshan Mountains, Shanxi Province, China	[36]
	-	-	*A. fumigatus* LN-4, *Melia azedarach* (stem bark), China	[37]
	-	-	*A. fumigatus* culture, *Crocus sativus* (lateral buds), Zhejiang, China	[38]
	-	-	*A. fumigatus* H22, seawater, Western Pacific	[39]
	-	-	*A. fumigatus*, *Delphinium grandiflorum*, Aba Tibetan Autonomous Prefecture, China	[40]
Secofumitremorgin B (**4**)	407	$C_{23}H_{25}N_3O_4$	*A. fumigatus* SD-406, cultured, deep-sea sediment, East China Sea, China	[29]
13-Oxofumitremorgin B (**5**)	477	$C_{27}H_{31}N_3O_5$	*A. fumigatus* Fres., cultured, *Stichopus japonicus* (marine holothurian), Lingshan Island, Qingdao, China	[32]
	-	-	*A. fumigatus* H22, seawater, Western Pacific	[39]
Derivative A-Fumitremorgin B (**6**)	495	$C_{27}H_{33}N_3O_6$	*A. fumigatus* Fres., cultured, *Stichopus japonicus* (marine holothurian), Lingshan Island, Qingdao, China.	[32]
Derivative A epimer-Fumitremorgin B (**7**)	495	$C_{27}H_{33}N_3O_6$	*A. fumigatus* Fres., cultured, *Stichopus japonicus* (marine holothurian), Lingshan Island, Qingdao, China.	[32]
Fumitremorgin C (**8**)	379	$C_{22}H_{25}N_3O_3$	*A. fumigatus* BM939, cultured, sea sediment sample, mouth of Oi River, Sizuoka prefecture, Japan	[30]
	-	-	*A. fumigatus* CANU A151, saline lake sand, Western Australia	[41]
	-	-	*A. fumigatus* CY018, *Cynodon dactylon* (leaf), Yancheng Biosphere Reserve, Jiangsu, China	[42]
	-	-	*A. fumigatus* Fres., cultured, *Stichopus japonicus* (marine holothurian), Lingshan Island, Qingdao, China.	[32]
	-	-	*A. fumigatus* YK-7, sea mud, intertidal zone, Yingkou, China	[33]
	-	-	*A. fumigatus*, *Erythrophloeum fordii* (stem), South of China	[43]
	-	-	*A. fumigatus* LN-4, *Melia azedarach* (stem bark, Meliaceae), Yangling, Shaanxi province, China	[34]
	-	-	*A. fumigatus* MBC-F1-10/ *Streptomyces bullii* co-culture, hyper-arid Atacama Desert soil, Chile	[35]

	-	-	*A. fumigatus* AR05, *Astragalus membranaceus* (root), Hengshan Mountains, Shanxi Province, China	[36]
	-	-	*A. fumigatus*, *Diphylleia sinensis* (rhizome), Honghegu, Shanxi, China	[44]
	-	-	*A. fumigatus*, *Heteroscyphus tener* (Chinese liverwort), Maoer Mountain, Guangxi Zhuang Autonomous Region, China	[45]
	-	-	*A. fumigatus*, coastal saline soil, Wudi, Shandong, China	[46]
	-	-	*A. fumigatus* SD-406, cultured, deep-sea sediment, East China Sea, China	[29]
	-	-	*A. fumigatus* HQD24, *Rhizophora mucronata* (flower), Dong Zhai Gang-Mangrove Garden, on Hainan Island, China	[47]
	-	-	*A. fumigatus* culture, *Crocus sativus* (lateral buds), Zhejiang, China	[38]
	-	-	*A. fumigatus* H22, seawater, Western Pacific	[39]
	-	-	*A. fumigatus* M1, *Aconitum brevicalcaratum* (roots), Kunming, Yunnan, China	[48]
	-	-	*A. fumigatus* UIAU-3F, soil, River Oyun in Kwara State, Nigeria	[49]
	-	-	*A. fumigatus* WJ-131, *Gardenia jasminoides* (stem), Kunming, Yunnan, China	[50]
	-	-	*A. fumigatus* GXIMD00544, Zhulin solar saltern, Beihai, China	[51]
12α-Fumitremorgin C (**9**)	379	$C_{22}H_{25}N_3O_3$	*A. fumigatus* CY018, *Cynodon dactylon* (leaf), Yancheng Biosphere Reserve, Jiangsu, China	[52]
	-	-	*A. fumigatus* LN-4, *Melia azedarach* (stem bark), China	[37
12α,13α-Dihydroxyfumitremorgin C (**10**)	411	$C_{22}H_{25}N_3O_5$	*A. fumigatus* DSM 790, Germany	[15]
	-	-	*A. fumigatus* BM939, cultured, sea sediment sample, mouth of Oi River, Sizuoka prefecture, Japan	[30]
	-	-	*A. fumigatus* Fres., cultured, *Stichopus japonicus* (marine holothurian), Lingshan Island, Qingdao, China.	[32]
	-	-	*A. fumigatus* YK-7, sea mud, intertidal zone, Yingkou, China	[33]
	-	-	*A. fumigatus*, *Erythrophloeum fordii* (stem), South of China	[43]
	-	-	*A. fumigatus* MBC-F1-10/ *Streptomyces bullii* co-culture, hyper-arid Atacama Desert soil, Chile	[35]
	-	-	*A. fumigatus*, *Diphylleia sinensis* (rhizome), Honghegu, Shanxi, China	[44]
	-	-	*A. fumigatus*, *Heteroscyphus tener* (Chinese liverwort), Maoer Mountain, Guangxi Zhuang Autonomous Region, China	[45]
	-	-	*A. fumigatus* MH773172, *Ligusticum wallichii*, Dujiangyan city, Suburb of Chengdu, China	[53]
	-	-	*A. fumigatus* SD-406, cultured, deep-sea sediment, East China Sea, China	[29]
	-	-	*A. fumigatus* M580, *Colochirus quadrangularis*, Co To-Thanh Lan Island, Vietnam	[54]
	-	-	*A. fumigatus* culture, *Crocus sativus* (lateral buds), Zhejiang, China	[38]
	-	-	*A. fumigatus* H22, seawater, Western Pacific	[39]
	-	-	*A. fumigatus* M1, *Aconitum brevicalcaratum* (roots), Kunming, Yunnan, China	[48]

	-	-	*A. fumigatus* WJ-131, *Gardenia jasminoides* (stem), Kunming, Yunnan, China	[50]
	-	-	*A. fumigatus*, *Delphinium grandiflorum*, Aba Tibetan Autonomous Prefecture, China	[40]
9α-Hydroxyfumitremorgin C (**11**)	395	$C_{22}H_{25}N_3O_4$	*A. fumigatus* YK-7, sea mud, intertidal zone, Yingkou, China	[33]
Demethoxyfumitremorgin C (**12**)	349	$C_{21}H_{23}N_3O_2$	*A. fumigatus* BM939, cultured, sea sediment sample, mouth of Oi River, Sizuoka prefecture, Japan	[30]
	-	-	*A. fumigatus* CY018, *Cynodon dactylon* (leaf), Yancheng Biosphere Reserve, Jiangsu, China	[52]
	-	-	*A. fumigatus*, *Heteroscyphus tener* (Chinese liverwort), Maoer Mountain, Guangxi Zhuang Autonomous Region, China	[45]
	-	-	*A. fumigatus*, coastal saline soil, Wudi, Shandong, China	[46]
	-	-	*A. fumigatus* H22, seawater, Western Pacific	[39]
12α-Hydroxy-13-oxofumitremorgin C (**13**)	409	$C_{22}H_{23}N_3O_5$	*A. fumigatus* H22, seawater, Western Pacific	[39]
12β-Hydroxy-13-oxofumitremorgin C (**14**)	409	$C_{22}H_{23}N_3O_5$	*A. fumigatus* VDL36, *Vaccinium dunalianum* (leaves), Wuding, Yunnan, China	[55]
rel-(8*S*,19*S*)-19,20-Dihydro-9,19,20-trihydroxy-8-methoxy-9-epi-fumitremorgin C (**15**)	459	$C_{23}H_{29}N_3O_7$	*A. fumigatus*, *Erythrophloeum fordii* (stem), South of China	[43]
rel-(8*R*)-9-Hydroxy-8-methoxy-18-epi-fumitremorgin C (**16**)	425	$C_{23}H_{27}N_3O_5$	*A. fumigatus*, *Erythrophloeum fordii* (stem), South of China	[43]
rel-(8*S*)-19,20-Dihydro-9,20-dihydroxy-8-methoxy-9,18-di-epi-fumitremorgin C (**17**)	443	$C_{23}H_{29}N_3O_6$	*A. fumigatus*, *Erythrophloeum fordii* (stem), South of China	[43]
Secofumitremorgin C (**18**)	363	$C_{21}H_{21}N_3O_3$	*A. fumigatus* GXIMD00544, Zhulin solar saltern, Beihai, China	[51]
Fumitremorgin D (**19**)	737	$C_{41}H_{43}N_3O_{10}$	*A. fumigatus*, *Diphylleia sinensis* (rhizome), Honghegu, Shanxi, China	[44]
Secofumitremorgin D (**20**)	363	$C_{21}H_{21}N_3O_3$	*A. fumigatus* GXIMD00544, Zhulin solar saltern, Beihai, China	[51]
Tryprostatin A (**21**)	381	$C_{22}H_{27}N_3O_3$	*A. fumigatus* BM939, cultured, sea sediment sample, mouth of Oi River, Sizuoka prefecture, Japan	[30]
	-	-	*A. fumigatus* Fres., cultured, *Stichopus japonicus* (marine holothurian), Lingshan Island, Qingdao, China.	[32]
	-	-	*A. fumigatus* CY018, *Cynodon dactylon* (leaf), Yancheng Biosphere Reserve, Jiangsu, China	[52]
	-	-	*A. fumigatus* LN-4, *Melia azedarach* (stem bark, Meliaceae), Yangling, Shaanxi province, China	[34]
	-	-	*A. fumigatus* LN-4, *Melia azedarach* (stem bark), China	[37]
	-	-	*A. fumigatus*, coastal saline soil, Wudi, Shandong, China	[46]
18-Oxotryprostatin A (**22**)	395	$C_{22}H_{25}N_3O_4$	*A. fumigatus*, *Erythrophloeum fordii* (stem), South of China	[43]
	-	-	*A. fumigatus* LN-4, *Melia azedarach* (stem bark), China	[37]
Fumitryprostatin A (**23**)	479	$C_{27}H_{33}N_3O_5$	*A. fumigatus*, Wuhan, China	[56]
Cyclotryprostatin A (**24**)	411	$C_{22}H_{25}N_3O_5$	*A. fumigatus* BM939, sea sediment, mouth of Oi River, Sizuoka prefecture, Japan	[57]
	-	-	*A. fumigatus* Fres., cultured, *Stichopus japonicus* (marine holothurian), Lingshan Island, Qingdao, China	[32]
	-	-	*A. fumigatus* YK-7, sea mud, intertidal zone, Yingkou, China	[33]

	-	-	*A. fumigatus* LN-4, *Melia azedarach* (stem bark, Meliaceae), Yangling, Shaanxi province, China	[34]
	-	-	*A. fumigatus* LN-4, *Melia azedarach* (stem bark), China	[37]
	-	-	*A. fumigatus*, coastal saline soil, Wudi, Shandong, China	[46]
	-	-	*A. fumigatus* culture, *Crocus sativus* (lateral buds), Zhejiang, China	[38]
	-	-	*A. fumigatus* VDL36, *Vaccinium dunalianum* (leaves), Wuding, Yunnan, China	[55]
13-Ethoxycyclotryprostatin A (**25**)	439	$C_{24}H_{29}N_3O_5$	*A. fumigatus* VDL36, *Vaccinium dunalianum* (leaves), Wuding, Yunnan, China	[55]
13-Dehydroxycyclotryprostatin A (**26**)	395	$C_{22}H_{25}N_3O_4$	*A. fumigatus* VDL36, *Vaccinium dunalianum* (leaves), Wuding, Yunnan, China	[55]
Tryprostatin B (**27**)	351	$C_{21}H_{25}N_3O_2$	*A. fumigatus* BM939, cultured, sea sediment sample, mouth of Oi River, Sizuoka prefecture, Japan	[30]]
	-	-	*A. fumigatus* Fres., cultured, *Stichopus japonicus* (marine holothurian), Lingshan Island, Qingdao, China.	[32]
	-	-	*A. fumigatus* CY018, *Cynodon dactylon* (leaf), Yancheng Biosphere Reserve, Jiangsu, China	[52]
	-	-	*A. fumigatus*, *Erythrophloeum fordii* (stem), South of China	[43]
	-	-	*A. fumigatus* M580, *Colochirus quadrangularis*, Co To-Thanh Lan Island, Vietnam	[54]
	-	-	*A. fumigatus* KFQG-2/*Alternaria alternata* KFZ-32 coculture, *Coffea arabica*, Baoshan, Yunnan, China	[58]
	-	-	*A. fumigatus* GXIMD00544, Zhulin solar saltern, Beihai, China	[51]
6-Hydroxytryprostatin B (**28**)	367	$C_{21}H_{25}N_3O_3$	*A. fumigatus* YK-7, sea mud, intertidal zone, Yingkou, China	[33]
Cyclotryprostatin B (**29**)	425	$C_{23}H_{27}N_3O_5$	*A. fumigatus* BM939, sea sediment, mouth of Oi River, Sizuoka prefecture, Japan	[57]
	-	-	*A. fumigatus* YK-7, sea mud, intertidal zone, Yingkou, China	[33]
	-	-	*A. fumigatus* LN-4, *Melia azedarach* (stem bark, Meliaceae), Yangling, Shaanxi province, China	[34]
	-	-	*A. fumigatus* AR05, *Astragalus membranaceus* (root), Hengshan Mountains, Shanxi Province, China	[36]
	-	-	*A. fumigatus* LN-4, *Melia azedarach* (stem bark), China	[37]
	-	-	*A. fumigatus* SD-406, cultured, deep-sea sediment, East China Sea, China	[29]
	-	-	*A. fumigatus* culture, *Crocus sativus* (lateral buds), Zhejiang, China	[38]
	-	-	*A. fumigatus* H22, seawater, Western Pacific	[39]
Prenylcyclotryprostatin B (**30**)	493	$C_{28}H_{35}N_3O_5$	*A. fumigatus* YK-7, sea mud, intertidal zone, Yingkou, China	[33]
20-Hydroxycyclotryprostatin B (**31**)	395	$C_{22}H_{25}N_3O_4$	*A. fumigatus* YK-7, sea mud, intertidal zone, Yingkou, China	[33]
	-	-	*A. fumigatus*, *Heteroscyphus tener* (Chinese liverwort), Maoer Mountain, Guangxi Zhuang Autonomous Region, China	[45]
Cyclotryprostatin C (**32**)	381	$C_{21}H_{23}N_3O_4$	*A. fumigatus* BM939, sea sediment, mouth of Oi River, Sizuoka prefecture, Japan	[57]
	-	-	*A. fumigatus*, *Heteroscyphus tener* (Chinese liverwort), Maoer Mountain, Guangxi Zhuang Autonomous Region, China	[45]

13-Dehydroxycyclotryprostatin C (**33**)	365	$C_{21}H_{23}N_3O_3$	*A. fumigatus*, *Heteroscyphus tener* (Chinese liverwort), Maoer Mountain, Guangxi Zhuang Autonomous Region, China	[45]
	-	-	*A. fumigatus* culture*, Crocus sativus* (lateral buds), Zhejiang, China	[38]
Cyclotryprostatin D (**34**)	379	$C_{21}H_{21}N_3O_4$	*A. fumigatus* BM939, sea sediment, mouth of Oi River, Sizuoka prefecture, Japan	[57]
Asperfumigatin (**35**)	511	$C_{27}H_{33}N_3O_7$	*A. fumigatus*, *Heteroscyphus tener* (Chinese liverwort), Maoer Mountain, Guangxi Zhuang Autonomous Region, China	[45]
	-	-	*A. fumigatus* H22, seawater, Western Pacific	[39]
	-	-	*A. fumigatus* GXIMD00544, Zhulin solar saltern, Beihai, China	[51]
	-	-	*A. fumigatus*, *Delphinium grandiflorum*, Aba Tibetan Autonomous Prefecture, China	[40]
12β,13β-hydroxy-asperfumigatin (**36**)	511	$C_{27}H_{33}N_3O_7$	*A. fumigatus* H22, seawater, Western Pacific	[39]
	-	-	*A. fumigatus* GXIMD00544, Zhulin solar saltern, Beihai, China	[51]
Verruculogen (**37**)	511	$C_{27}H_{33}N_3O_7$	*A. fumigatus* strains (AFu-3, AFu-4, AFu-5, and AFu-6), molded silage, USA	[59]
	-	-	*A. fumigatus* BM939, cultured, sea sediment sample, mouth of Oi River, Sizuoka prefecture, Japan	[30]
	-	-	*A. fumigatus* Fres., cultured, *Stichopus japonicus* (marine holothurian), Lingshan Island, Qingdao, China.	[32]
	-	-	*A. fumigatus* CY018, *Cynodon dactylon* (leaf), Yancheng Biosphere Reserve, Jiangsu, China	[52]
	-	-	*A. fumigatus* YK-7, sea mud, intertidal zone, Yingkou, China	[33]
	-	-	*A. fumigatus* LN-4, *Melia azedarach* (stem bark, Meliaceae), Yangling, Shaanxi province, China	[34]
	-	-	*A. fumigatus* MBC-F1-10/ *Streptomyces bullii* co-culture, hyper-arid Atacama Desert soil, Chile	[35]
	-	-	*A. fumigatus* AR05, *Astragalus membranaceus* (root), Hengshan Mountains, Shanxi Province, China	[36]
	-	-	*A. fumigatus* LN-4, *Melia azedarach* (stem bark), China	[37] [38]
	-	-	*A. fumigatus*, *Diphylleia sinensis* (rhizome), Honghegu, Shanxi, China	[44]
	-	-	*A. fumigatus* MH773172, *Ligusticum wallichii*, Dujiangyan city, Suburb of Chengdu, China	[53]
	-	-	*A. fumigatus* culture*, Crocus sativus* (lateral buds), Zhejiang, China	[38]
	-	-	*A. fumigatus* H22, seawater, Western Pacific	[39]
13-Oxoverruculogen (**38**)	509	$C_{27}H_{31}N_3O_7$	*A. fumigatus* Fres., cultured, *Stichopus japonicus* (marine holothurian), Lingshan Island, Qingdao, China	[32]
	-	-	*A. fumigatus*, *Diphylleia sinensis* (rhizome), Honghegu, Shanxi, China	[44]
Verruculogen TR-2 (**39**)	429	$C_{22}H_{27}N_3O_6$	*A. fumigatus* strains (AFu-3, AFu-4, AFu-5, and AFu-6), molded silage, USA	[59]
	-	-	*A. fumigatus* LN-4, *Melia azedarach* (stem bark, Meliaceae), Yangling, Shaanxi province, China	[34]
	-	-	*A. fumigatus* LN-4, *Melia azedarach* (stem bark), China	[37]

	-	-	*A. fumigatus*, *Heteroscyphus tener* (Chinese liverwort), Maoer Mountain, Guangxi Zhuang Autonomous Region, China	[45]
	-	-	*A. fumigatus* M1, *Aconitum brevicalcaratum* (roots), Kunming, Yunnan, China	[48]
	-	-	*A. fumigatus* GXIMD00544, Zhulin solar saltern, Beihai, China	[51]
	-	-	*A. fumigatus*, *Delphinium grandiflorum*, Aba Tibetan Autonomous Prefecture, China	[40]
12β-Hydroxyverruculogen TR-2 (**40**)	429	$C_{22}H_{27}N_3O_6$	*A. fumigatus* LN-4, *Melia azedarach* (stem bark, Meliaceae), Yangling, Shaanxi province, China	[34]
	-	-	*A. fumigatus* LN-4, *Melia azedarach* (stem bark), China	[37]
	-	-	*A. fumigatus* GXIMD00544, Zhulin solar saltern, Beihai, China	[51]
12β-Hydroxy-13α-methoxyverruculogen TR-2 (**41**)	443	$C_{23}H_{29}N_3O_6$	*A. fumigatus* LN-4, *Melia azedarach* (stem bark, Meliaceae), Yangling, Shaanxi province, China	[34]
	-	-	*A. fumigatus* LN-4, *Melia azedarach* (stem bark), China	[37]
Spirotryprostatin A (**42**)	395	$C_{22}H_{25}N_3O_4$	*A. fumigatus* BM939, sea sediment, mouth of Oi River, Sizuoka prefecture, Japan	[60]
	-	-	*A. fumigatus* Fres., cultured, *Stichopus japonicus* (marine holothurian), Lingshan Island, Qingdao, China	[32]
	-	-	*A. fumigatus* CY018, *Cynodon dactylon* (leaf), Yancheng Biosphere Reserve, Jiangsu, China	[52]
	-	-	*A. fumigatus* KMM 4631, *Sinularia* sp. (superficial mycobiota, soft coral), Kunachir island, Kuril Islands, Russia	[61]
	-	-	*A. fumigatus* MBC-F1-10/ *Streptomyces bullii* co-culture, hyper-arid Atacama Desert soil, Chile	[35]
	-	-	*A. fumigatus* LN-4, *Melia azedarach* (stem bark), China	[37]
	-	-	*A. fumigatus* KR019681, *Edgeworthia chrysantha*, coastal region of Hangzhou Bay, Hangzhou, China	[62]
	-	-	*A. fumigatus* culture, *Crocus sativus* (lateral buds), Zhejiang, China	[38]
	-	-	*A. fumigatus* H22, seawater, Western Pacific	[39]
	-	-	*A. fumigatus* culture, *Scutellaria formosana* (flowers), Hainan, Yunnan, Guangdong, southern China	[63]
Spiro [5H,10H-dipyrrolo[1,2-a:1`,2`-d]pyrazine-2-(3H),2`-[2H]indole]-3`,5,10(1`H)-trione (**43**)	427	$C_{22}H_{25}N_3O_6$	*A. fumigatus* Fres., cultured, *Stichopus japonicus* (marine holothurian), Lingshan Island, Qingdao, China	[32]
	-	-	*A. fumigatus* KMM 4631, *Sinularia* sp. (superficial mycobiota, soft coral), Kunachir island, Kuril Islands, Russia	[61]
	-	-	*A. fumigatus*, *Erythrophloeum fordii* (stem), South of China	[64]
	-	-	*A. fumigatus*, *Delphinium grandiflorum*, Aba Tibetan Autonomous Prefecture, China	[40]
(3*S*,8*S*,9*S*,18*S*)-8,9-Dihydroxyspirotryprostatin A (**44**)	427	$C_{22}H_{25}N_3O_6$	*A. fumigatus*, *Erythrophloeum fordii* (stem), South of China	[43]
Spirotryprostatin B (**45**)	363	$C_{21}H_{21}N_3O_3$	*A. fumigatus* BM939, sea sediment, mouth of Oi River, Sizuoka prefecture, Japan	[60]
	-	-	*A. fumigatus*, *Heteroscyphus tener* (Chinese liverwort), Maoer Mountain, Guangxi Zhuang Autonomous Region, China	[45]

	-	-	*A. fumigatus* culture, *Scutellaria formosana* (flowers), Hainan, Yunnan, Guangdong, southern China	[63]
6-Methoxyspirotryprostatin B = Spirotryprostatin G (**46**)	393	$C_{22}H_{23}N_3O_4$	*A. fumigatus* CY018, *Cynodon dactylon* (leaf), Yancheng Biosphere Reserve, Jiangsu, China	[52]
	-	-	*A. fumigatus* KMM 4631, *Sinularia* sp. (superficial mycobiota, soft coral), Kunachir island, Kuril Islands, Russia	[61]
	-	-	*A. fumigatus* LN-4, *Melia azedarach* (stem bark, Meliaceae), Yangling, Shaanxi province, China	[34]
	-	-	*A. fumigatus* MBC-F1-10/ *Streptomyces bullii* co-culture, hyper-arid Atacama Desert soil, Chile	[35]
	-	-	*A. fumigatus* LN-4, *Melia azedarach* (stem bark), China	[37]
	-	-	*A. fumigatus*, *Erythrophloeum fordii* (stem), South of China	[64]
			A. fumigatus KR019681, *Edgeworthia chrysantha*, coastal region of Hangzhou Bay, Hangzhou, China	[62]
	-	-	*A. fumigatus* M580, *Colochirus quadrangularis*, Co To-Thanh Lan Island, Vietnam	[54]
	-	-	*A. fumigatus* H22, seawater, Western Pacific	[39]
Spirotryprostatin C (**47**)	495	$C_{27}H_{33}N_3O_6$	*A. fumigatus* Fres., cultured, *Stichopus japonicus* (marine holothurian), Lingshan Island, Qingdao, China	[32]
	-	-	*A. fumigatus* H22, seawater, Western Pacific	[39]
	-	-	*A. fumigatus* GXIMD00544, Zhulin solar saltern, Beihai, China	[51]
Spirotryprostatin D (**48**)	511	$C_{27}H_{33}N_3O_7$	*A. fumigatus* Fres., cultured, *Stichopus japonicus* (marine holothurian), Lingshan Island, Qingdao, China	[32]
Spirotryprostatin E (**49**)	527	$C_{27}H_{33}N_3O_8$	*A. fumigatus* Fres., cultured, *Stichopus japonicus* (marine holothurian), Lingshan Island, Qingdao, China	[32]
Spirotryprostatin K (**50**)	383	$C_{21}H_{25}N_3O_4$	*A. fumigatus*, *Erythrophloeum fordii* (stem), South of China	[64]
Indole-quinazoline alkaloids				
Tryptoquivaline = Tryptoquivaline C (FTC) (**51**)	546	$C_{29}H_{30}N_4O_7$	*A. fumigatus* 0011, Cultured, Japan	[65-67]
Nortryptoquivaline = Tryptoquivaline D (FTD) (**52**)	532	$C_{28}H_{28}N_4O_7$	*A. fumigatus* 0011, Cultured, Japan	[66,67]
Deoxytryptoquivaline (**53**)	530	$C_{29}H_{30}N_4O_6$	*A. fumigatus* 0011, Cultured, Japan	[66]
Deoxynortryptoquivaline (**54**)	516	$C_{28}H_{28}N_4O_6$	*A. fumigatus* 0011, Cultured, Japan	[66]
Nortryptoquivalone (**55**)	488	$C_{26}H_{24}N_4O_5$	*A. fumigatus* 0011, Cultured, Japan	[66]
Deoxynortryptoquivalone (**56**)	472	$C_{26}H_{24}N_4O_5$	*A. fumigatus* 0011, Cultured, Japan	[66]
Tryptoquivaline E (FTE) (**57**)	418	$C_{22}H_{18}N_4O_5$	*A. fumigatus* 0011, Cultured, Japan	65]
Tryptoquivaline F (FTF) = Fumitremorgin F (**58**)	402	$C_{22}H_{18}N_4O_4$	*A. fumigatus* 0011, Cultured, Japan	65]
	-	-	*A. fumigatus* CY018, *Cynodon dactylon* (leaf), Yancheng Biosphere Reserve, Jiangsu, China	[52]
	-	-	*A. fumigatus* NRRL 35693, cultured, France	[13]
	-	-	*A. fumigatus* KMM 4631, *Sinularia* sp. (superficial mycobiota, soft coral), Kunachir island, Kuril Islands, Russia	[61]
2-*epi*-Tryptoquivaline F (**59**)	402	$C_{22}H_{18}N_4O_4$	*A. fumigatus* H22, seawater, Western Pacific	[39]
	-	-	*A. fumigatus* WJ-131, *Gardenia jasminoides* (stem), Kunming, Yunnan, China	[50]
Tryptoquivaline G (FTG) (**60**)	432	$C_{23}H_{20}N_4O_5$	*A. fumigatus* 0011, Cultured, Japan	65]

Tryptoquivaline H (FTH) (**61**)	418	$C_{22}H_{18}N_4O_5$	*A. fumigatus* 0011, Cultured, Japan	65]
Tryptoquivaline I (FTI) (**62**)	502	$C_{27}H_{26}N_4O_6$	*A. fumigatus* 0011, Cultured, Japan	[66]
	-	-	*A. fumigatus* IFM 54246, soil, Brazil	[68]
Tryptoquivaline J (FTJ) (**63**)	402	$C_{22}H_{18}N_4O_4$	*A. fumigatus* 0011, Cultured, Japan	[66]
	-	-	*A. fumigatus* KMM 4631, cultured, marine isolate, Russia	[31]
	-	-	*A. fumigatus* HQD24, *Rhizophora mucronata* (flower), Dong Zhai Gang-Mangrove Garden, on Hainan Island, China	[47]
Tryptoquivaline L (FTL) (**64**)	432	$C_{23}H_{20}N_4O_5$	*A. fumigatus* IFM 4482, cultured, Japan	[67]
Tryptoquivaline M (FTM) (**65**)	532	$C_{28}H_{28}N_4O_7$	*A. fumigatus* IFM 4482, cultured, Japan	[67]
Tryptoquivaline N (FTN) (**66**)	472	$C_{26}H_{24}N_4O_5$	*A. fumigatus* IFM 4482, cultured, Japan	[67]
Tryptoquivaline O (FTO) (**67**)	402	$C_{22}H_{18}N_4O_4$	*A. fumigatus* LN-4, *Melia azedarach* (stem bark, Meliaceae), Yangling, Shaanxi province, China	[34]
Fumiquinazoline A (**68**)	445	$C_{24}H_{23}N_5O_4$	*A. fumigatus, Pseudolabrus japonicus* (GIT of the saltwater fish), Osaka, Japan	[69]
	-	-	*A. fumigatus* LN-4, *Melia azedarach* (stem bark, Meliaceae), Yangling, Shaanxi province, China	[34]
	-	-	*A. fumigatus* MH773172, *Ligusticum wallichii*, Dujiangyan city, Suburb of Chengdu, China	[53]
3-Hydroxyfumiquinazoline A (**69**)	461	$C_{24}H_{23}N_5O_5$	*A. fumigatus* LN-4, *Melia azedarach* (stem bark, Meliaceae), Yangling, Shaanxi province, China	[34]
Fumiquinazoline B (**70**)	445	$C_{24}H_{23}N_5O_4$	*A. fumigatus, Pseudolabrus japonicus* (GIT of the saltwater fish), Osaka, Japan	[69]
	-	-	*A. fumigatus*, coastal saline soil, Wudi, Shandong, China	[46]
Fumiquinazoline C (**71**)	443	$C_{24}H_{21}N_5O_4$	*A. fumigatus, Pseudolabrus japonicus* (GIT of the saltwater fish), Osaka, Japan	[69]
	-	-	*A. fumigatus* KMM 4631, cultured, marine isolate, Russia	[31]
	-	-	*A. fumigatus* NRRL 35693, cultured, France	[13]
	-	-	*A. fumigatus*, *Diphylleia sinensis* (rhizome), Honghegu, Shanxi, China	[44]
	-	-	*A. fumigatus*, *Heteroscyphus tener* (Chinese liverwort), Maoer Mountain, Guangxi Zhuang Autonomous Region, China	[45]
	-	-	*A. fumigatus*, coastal saline soil, Wudi, Shandong, China	[46]
	-	-	*A. fumigatus* SCSIO 41012, deep-sea sediments, Indian Ocean	[70]
	-	-	*A. fumigatus* SD-406, cultured, deep-sea sediment, East China Sea, China	[29]
	-	-	*A. fumigatus* M580, *Colochirus quadrangularis*, Co To-Thanh Lan Island, Vietnam	[54]
	-	-	*A. fumigatus* H22, seawater, Western Pacific	[39]
	-	-	*A. fumigatus*, *Delphinium grandiflorum*, Aba Tibetan Autonomous Prefecture, China	[40]
29-Hydroxyfumiquinazoline C (**72**)	459	$C_{24}H_{21}N_5O_5$	*A. fumigatus* SD-406, cultured, deep-sea sediment, East China Sea, China	[29]
Fumiquinazoline D (**73**)	443	$C_{24}H_{21}N_5O_4$	*A. fumigatus* KMM 4631, cultured, marine isolate, Russia	[31]
	-	-	*A. fumigatus* R7 culture, *Ipomoea batatas* (leaves), Egypt	[71]
	-	-	*A. fumigatus* M580, *Colochirus quadrangularis*, Co To-Thanh Lan Island, Vietnam	[54]
	-	-	*A. fumigatus*, *Delphinium grandiflorum*, Aba Tibetan Autonomous Prefecture, China	[40]

Fumiquinazoline F (**74**)	358	$C_{21}H_{18}N_4O_2$	*A. fumigatus* LN-4, *Melia azedarach* (stem bark, Meliaceae), Yangling, Shaanxi province, China	[34]
	-	-	*A. fumigatus* R7 culture, *Ipomoea batatas* (leaves), Egypt	[71]
Fumiquinazoline G (**75**)	358	$C_{21}H_{18}N_4O_2$	*A. fumigatus* LN-4, *Melia azedarach* (stem bark, Meliaceae), Yangling, Shaanxi province, China	[34]
	-	-	*A. fumigatus* SCSIO 41012, deep-sea sediments, Indian Ocean	[70]
Fumiquinazoline J (**76**)	342	$C_{20}H_{14}N_4O_2$	*A. fumigatus* AR05, *Astragalus membranaceus* (root), Hengshan Mountains, Shanxi Province, China	[36]
	-	-	*A. fumigatus*, *Heteroscyphus tener* (Chinese liverwort), Maoer Mountain, Guangxi Zhuang Autonomous Region, China	[45]
	-	-	*A. fumigatus* HQD24, *Rhizophora stylosa* (roots), South China Sea, China	[72]
	-	-	*A. fumigatus* M580, *Colochirus quadrangularis*, Co To-Thanh Lan Island, Vietnam	[54]
	-	-	*A. fumigatus* SAI12, *Sonneratia apetala* (leaves), Dongzhaigang, Hainan, China	[73]
	-	-	*A. fumigatus* WJ-131, *Gardenia jasminoides* (stem), Kunming, Yunnan, China	[50]
	-	-	*A. fumigatus* GXIMD00544, Zhulin solar saltern, Beihai, China	[51]
	-	-	*A. fumigatus*, *Delphinium grandiflorum*, Aba Tibetan Autonomous Prefecture, China	[40]
Fumiquinazoline K (**77**)	356	$C_{21}H_{16}N_4O_2$	*A. fumigatus* KMM 4631, *Sinularia* sp. (superficial mycobiota, soft coral), Kunachir island, Kuril Islands, Russia	[61]
Fumigatoside B (**79**)	623	$C_{30}H_{33}N_5O_{10}$	*A. fumigatus*, *Nemopilema nomurai* (jellyfish), Southern coast of Korea	[74]
	-	-	*A. fumigatus* SAI12, *Sonneratia apetala* (leaves), Dongzhaigang, Hainan, China	[73]
Fumiquinazolinine (**78**)	434	$C_{23}H_{22}N_4O_5$	*A. fumigatus* VDL36, *Vaccinium dunalianum* (leaves), Wuding, Yunnan, China	[55]
Fumigatoside C (**80**)	607	$C_{30}H_{33}N_5O_9$	*A. fumigatus*, *Nemopilema nomurai* (jellyfish), Southern coast of Korea	[74]
Fumigatoside D (**81**)	607	$C_{30}H_{33}N_5O_9$	*A. fumigatus*, *Nemopilema nomurai* (jellyfish), Southern coast of Korea	[74]
Fumigatoside E (**82**)	372	$C_{21}H_{16}N_4O_3$	*A. fumigatus* SCSIO 41012, deep-sea sediments, Indian Ocean	[70]
Fumigatoside F (**83**)	420	$C_{22}H_{20}N_4O_5$	*A. fumigatus* SCSIO 41012, deep-sea sediments, Indian Ocean	[70]
Fumigatoside G (**84**)	605	$C_{30}H_{31}N_5O_9$	*A. fumigatus* SAI12, *Sonneratia apetala* (leaves), Dongzhaigang, Hainan, China	[73]
Fumigatoside H (**85**)	582	$C_{28}H_{30}N_4O_{10}$	*A. fumigatus* SAI12, *Sonneratia apetala* (leaves), Dongzhaigang, Hainan, China	[73]
(+)-Alantrypinone (**86**)	372	$C_{21}H_{16}N_4O_3$	*A. fumigatus* H22, seawater, Western Pacific	[39]
Oxoglyantrypine (**87**)	358	$C_{20}H_{14}N_4O_3$	*A. fumigatus* H22, seawater, Western Pacific	[39]
Chaetominine (**88**)	402	$C_{22}H_{18}N_4O_4$	*A. fumigatus*, *Heteroscyphus tener* (Chinese liverwort), Maoer Mountain, Guangxi Zhuang Autonomous Region, China	[45]
	-	-	*A. fumigatus* MF029, *Hymeniacidon perleve* (sponge), Bohai Sea, China	[75]
	-	-	*A. fumigatus*, *Albizia lucidior* (leaf), Zoological Garden, Giza, Egypt	[76]

	-	-	*A. fumigatus* HQD24, *Rhizophora mucronata* (flower), Dong Zhai Gang-Mangrove Garden, on Hainan Island, China	[47]
	-	-	*A. fumigatus* H22, seawater, Western Pacific	[39]
	-	-	*A. fumigatus* WJ-131, *Gardenia jasminoides* (stem), Kunming, Yunnan, China	[50]
	-	-	*A. fumigatus* GXIMD00544, Zhulin solar saltern, Beihai, China	[51]
	-	-	*A. fumigatus*, *Delphinium grandiflorum*, Aba Tibetan Autonomous Prefecture, China	[40]
	-	-	*A. fumigatus* VDL36, *Vaccinium dunalianum* (leaves), Wuding, Yunnan, China	[55]
Chaetominine A (**89**)	418	$C_{22}H_{18}N_4O_5$	*A. fumigatus* MF029, *Hymeniacidon perleve* (sponge), Bohai Sea, China	[75]
Isochaetominine = 11-*epi*-chaetominine (**90**)	402	$C_{22}H_{18}N_4O_4$	*A. fumigatus*, *Heteroscyphus tener* (Chinese liverwort), Maoer Mountain, Guangxi Zhuang Autonomous Region, China	[45]
	-	-	*A. fumigatus* H22, seawater, Western Pacific	[39]
Methyl (*S*)-{2-((3*R*,4a*S*,9a*R*)-hydroxy-2-oxo-3-(4-oxoquinazolin-3(4H)-yl)-2,3,4,4a,9,9a-hexahydro-1*H*-pyrido[2,3-b]indol-1-yl)propanoate} (**91**)	434	$C_{23}H_{22}N_4O_5$	*A. fumigatus* GXIMD00544, Zhulin solar saltern, Beihai, China	[51]

Table S2: List of diketopiperazine alkaloids isolated from *Aspergillus fumigatus*.

Compound Name	M. Wt.	Mol. Formula	Strain, Host, and Location	Ref.
3β-Hydroxy cyclo-L-tryptophyl-L-proline (**92**)	299	$C_{16}H_{17}N_3O_3$	*A. fumigatus* Fres., cultured, *Stichopus japonicus* (marine holothurian), Lingshan Island, Qingdao, China	[32]
Cyclo-L-tryptophyl-L-proline = Brevianamide F (**93**)	283	$C_{16}H_{17}N_3O_2$	*A. fumigatus* Fres., cultured, *Stichopus japonicus* (marine holothurian), Lingshan Island, Qingdao, China	[32]
	-	-	*A. fumigatus* LN-4, *Melia azedarach* (stem bark, Meliaceae), Yangling, Shaanxi province, China	[34]
	-	-	*A. fumigatus* MBC-F1-10/ *Streptomyces bullii* co-culture, hyper-arid Atacama Desert soil, Chile	[35]
	-	-	*A. fumigatus* LN-4, *Melia azedarach* (stem bark), China	[37]
	-	-	*A. fumigatus*, *Heteroscyphus tener* (Chinese liverwort), Maoer Mountain, Guangxi Zhuang Autonomous Region, China	[45]
	-	-	*A. fumigatus* M580, *Colochirus quadrangularis*, Co To-Thanh Lan Island, Vietnam	[54]
	-	-	*A. fumigatus* M1, *Aconitum brevicalcaratum* (roots), Kunming, Yunnan, China	[48]
	-	-	*A. fumigatus* KFQG-2/*Alternaria alternata* KFZ-32 coculture, *Coffea arabica*, Baoshan, Yunnan, China	[58]
	-	-	*A. fumigatus* culture, *Scutellaria formosana* (flowers), Hainan, Yunnan, Guangdong, southern China	[63]
N-Prenyl-cyclo-L-tryptophyl-L-proline (**94**)	351	$C_{21}H_{25}N_3O_2$	*A. fumigatus* Fres., cultured, *Stichopus japonicus* (marine holothurian), Lingshan Island, Qingdao, China	[32]
	-	-	*A. fumigatus*, *Erythrophloeum fordii* (stem), South of China	[43]
Cyclo (L)-Pro-(L)-Phe (**95**)	244	$C_{14}H_{16}N_2O_2$	*A. fumigatus*, soil, Pantanal-MS, Brazil	[81]
	-	-	*A. fumigatus* M580, *Colochirus quadrangularis*, Co To-Thanh Lan Island, Vietnam	[54]
Cyclo-(Gly-Phe) (**96**)	204	$C_{11}H_{12}N_2O_2$	*A. fumigatus* LN-4, *Melia azedarach* (stem bark, Meliaceae), Yangling, Shaanxi province, China	[34]
Cyclo (L)-4-OH-Pro-(L)-Phe (**97**)	260	$C_{14}H_{16}N_2O_3$	*A. fumigatus*, soil, Pantanal-MS, Brazil	[81]
	-	-	*A. fumigatus* LN-4, *Melia azedarach* (stem bark, Meliaceae), Yangling, Shaanxi province, China	[34]
	-	-	*A. fumigatus* culture, *Scutellaria formosana* (flowers), Hainan, Yunnan, Guangdong, southern China	[63]
Cyclo-(L-Tyr-L-Pro) (**98**)	260	$C_{14}H_{16}N_2O_3$	*A. fumigatus* culture, *Scutellaria formosana* (flowers), Hainan, Yunnan, Guangdong, southern China	[63]
(3*R*,6*S*)-3-Benzyl-6-(hydroxymethyl)-3-	264	$C_{13}H_{16}N_2O_4$	*A. fumigatus*, marine sediments, Jiaozhou Bay, China	[79]

methoxypiperazine-2,5-dione (**99**)				
(3*S*,6*S*)-3-Benzyl-6-(hydroxymethyl)-3-methoxypiperazine-2,5-dione (**100**)	264	$C_{13}H_{16}N_2O_4$	*A. fumigatus*, marine sediments, Jiaozhou Bay, China	[79]
(*R*)-6-Benzylidene-3-hydroxy-3-(hydroxymethyl)piperazine-2,5-dione (**101**)	248	$C_{12}H_{12}N_2O_4$	*A. fumigatus*, marine sediments, Jiaozhou Bay, China	[79]
Cyclo-(L-phenyl-L-valine) (**102**)	246	$C_{14}H_{18}N_2O_2$	*A. fumigatus* culture, *Scutellaria formosana* (flowers), Hainan, Yunnan, Guangdong, southern China	[63]
Cyclo(L-phenyl-L-isoleucine) (**103**)	260	$C_{15}H_{20}N_2O_2$	*A. fumigatus* culture, *Scutellaria formosana* (flowers), Hainan, Yunnan, Guangdong, southern China	[63]
Cyclo (L)-Pro-(L)-Leu (**104**)	210	$C_{11}H_{18}N_2O_2$	*A. fumigatus*, soil, Pantanal-MS, Brazil	[81]
	-	-	*A. fumigatus*, *Erythrophloeum fordii* (stem), South of China	[43]
	-	-	*A. fumigatus* M580, *Colochirus quadrangularis*, Co To-Thanh Lan Island, Vietnam	[54]
Cyclo-(L-Val-L-Leu) (**105**)	212	$C_{11}H_{20}N_2O_2$	*A. fumigatus, Erythrophleum fordii* (stem), China	[80]
Cyclo (L)-Pro- (L)-Pro (**106**)	194	$C_{10}H_{14}N_2O_2$	*A. fumigatus*, soil, Pantanal-MS, Brazil	[81]
Cyclo-(Pro-*trans*-4-OH-Pro) (**107**)	210	$C_{10}H_{14}N_2O_3$	*A. fumigatus* LN-4, *Melia azedarach* (stem bark, Meliaceae), Yangling, Shaanxi province, China	[34]
Cyclo-(Ser-*trans*-4-OH-Pro) (**108**)	200	$C_8H_{12}N_2O_4$	*A. fumigatus* LN-4, *Melia azedarach* (stem bark, Meliaceae), Yangling, Shaanxi province, China	[34]
Cyclo-(Ala-*trans*-4-OH-Pro) (**109**)	184	$C_8H_{12}N_2O_3$	*A. fumigatus* LN-4, *Melia azedarach* (stem bark, Meliaceae), Yangling, Shaanxi province, China	[34]
Cyclo (L)-Pro-(L)-Val (**110**)	196	$C_{10}H_{16}N_2O_2$	*A. fumigatus*, soil, Pantanal-MS, Brazil	[81]
Cyclo (L)-4-OH-Pro-(L)-Leu (**111**)	226	$C_{11}H_{18}N_2O_3$	*A. fumigatus*, soil, Pantanal-MS, Brazil	[81]
	-	-	*A. fumigatus* LN-4, *Melia azedarach* (stem bark, Meliaceae), Yangling, Shaanxi province, China	[34]
	-	-	*A. fumigatus* culture, *Scutellaria formosana* (flowers), Hainan, Yunnan, Guangdong, southern China	[63]
Cyclo (L-isoleucyl-L-prolyl) (**112**)	210	$C_{11}H_{18}N_2O_2$	*A. fumigatus*, *Erythrophloeum fordii* (stem), South of China	[43]
Cyclo-(Pro-Ser) (**113**)	184	$C_8H_{12}N_2O_3$	*A. fumigatus* LN-4, *Melia azedarach* (stem bark, Meliaceae), Yangling, Shaanxi province, China	[34]
Cyclo-(Pro-Ala) (**114**)	168	$C_8H_{12}N_2O_2$	*A. fumigatus* LN-4, *Melia azedarach* (stem bark, Meliaceae), Yangling, Shaanxi province, China	[34]
Cyclo-(D-Pro-L-Ala) (**115**)	168	$C_8H_{12}N_2O_2$	*A. fumigatus* LN-4, *Melia azedarach* (stem bark, Meliaceae), Yangling, Shaanxi province, China	[34]
	-	-	*A. fumigatus, Scutellaria formosana* (flowers), Hainan, Yunnan, Guangdong, southern China	[63]

Cyclo (L)-Pro-Gly (**116**)	154	$C_7H_{10}N_2O_2$	*A. fumigatus*, soil, Pantanal-MS, Brazil	[81]
	-	-	*A. fumigatus* LN-4, *Melia azedarach* (stem bark, Meliaceae), Yangling, Shaanxi province, China	[34]
	-	-	*A. fumigatus, Scutellaria formosana* (flowers), Hainan, Yunnan, Guangdong, southern China	[63]
Cyclo (L-Ala-L-Leu) (**117**)	184	$C_9H_{16}N_2O_2$	*A. fumigatus* CY018, *Cynodon dactylon* (leaf), Yancheng Biosphere Reserve, Jiangsu, China	[42]
Cyclo (L-Ala-L-Ile) (**118**)	184	$C_9H_{16}N_2O_2$	*A. fumigatus* CY018, *Cynodon dactylon* (leaf), Yancheng Biosphere Reserve, Jiangsu, China	[42]
Cyclo-(L-Ala-L-Val) (**119**)	170	$C_8H_{14}N_2O_2$	*A. fumigatus*, marine sediments, Jiaozhou Bay, China	[79]
Cyclo-(Gly-Ala) (**120**)	128	$C_5H_8N_2O_2$	*A. fumigatus* LN-4, *Melia azedarach* (stem bark, Meliaceae), Yangling, Shaanxi province, China	[34]

Table S3: List of clavine-type ergot and spiro-heterocyclic γ-lactam alkaloids isolated from *Aspergillus fumigatus*.

**Compound Name/chemical class**	**M. Wt.**	**Mol. Formula**	**Strain, Host, and Location**	**Ref.**
Clavine-type ergot alkaloids				
Fumigaclavine A (**121**)	298	$C_{18}H_{22}N_2O_2$	*A. fumigatus* strains (AFu-3, AFu-4, AFu-5, and AFu-6), molded silage, USA	[59]
	-	-	*A. fumigatus*, *Diphylleia sinensis* (rhizome), Honghegu, Shanxi, China	[44]
	-	-	*A. fumigatus* WJ-131, *Gardenia jasminoides* (stem), Kunming, Yunnan, China	[50]
Secofumigaclavine A (**122**)	398	$C_{23}H_{30}N_2O_4$	*A. fumigatus* CY018, human feces, China	[82]
Fumigaclavine B (**123**)	256	$C_{16}H_{20}NO_2$	*A. fumigatus* strains (AFu-3, AFu-4, AFu-5, and AFu-6), molded silage, USA	[59]
	-	-	*A. fumigatus* LN-4, *Melia azedarach* (stem bark, Meliaceae), Yangling, Shaanxi province, China	[34]
Secofumigaclavine B (**124**)	398	$C_{23}H_{30}N_2O_4$	*A. fumigatus* CY018, human feces, China	[82]
Fumigaclavine C (**125**)	366	$C_{23}H_{30}N_2O_2$	*A. fumigatus* strains (AFu-3, AFu-4, AFu-5, and AFu-6), molded silage, USA	[59]
	-	-	*A. fumigatus* CY018, *Cynodon dactylon* (leaf), Yancheng Biosphere Reserve, Jiangsu, China	[42]
	-	-	*A. fumigatus*, surface of marine green algae, Seosaeng-myeon, Ulsan, Korea	[83]
	-	-	*A. fumigatus*, *Diphylleia sinensis* (rhizome), Honghegu, Shanxi, China	[44]
	-	-	*A. fumigatus*, *Heteroscyphus tener* (Chinese liverwort), Maoer Mountain, Guangxi Zhuang Autonomous Region, China	[45]
	-	-	*A. fumigatus* SZW01, sediment, Shenzhen, Guangdong, China	[84]
	-	-	*A. fumigatus*, *Ceriops decandra* (leaves), Kolagachia, Sundarbans, Bangladesh	[85]
	-	-	*A. fumigatus* H22, seawater, Western Pacific	[39]
	-	-	*A. fumigatus* WJ-131, *Gardenia jasminoides* (stem), Kunming, Yunnan, China	[50]
	-	-	*A. fumigatus* GXIMD00544, Zhulin solar saltern, Beihai, China	[51]
	-	-	*A. fumigatus* VDL36, *Vaccinium dunalianum* (leaves), Wuding, Yunnan, China	[55]
Secofumigaclavine C (**126**)	384	$C_{22}H_{28}N_2O_4$	*A. fumigatus* CY018, human feces, China	[82]
9-Deacetylfumigaclavine C (**127**)	324	$C_{21}H_{28}N_2O$	*A. fumigatus* CY018, *Cynodon dactylon* (leaf), Yancheng Biosphere Reserve, Jiangsu, China	[52]
	-	-	*A. fumigatus* CY018, human feces, China	[82]
9-Deacetoxyfumigaclavine C (**128**)	308	$C_{21}H_{28}N_2$	*A. fumigatus* CY018, *Cynodon dactylon* (leaf), Yancheng Biosphere Reserve, Jiangsu, China	[52]
Fumigaclavine D (**129**)	352	$C_{22}H_{28}N_2O_2$	*A. fumigatus* CY018, human feces, China	[82]
Secofumigaclavine D (**130**)	384	$C_{22}H_{28}N_2O_4$	*A. fumigatus* CY018, human feces, China	[82]
Fumigaclavine E (**131**)	324	$C_{21}H_{28}N_2O$	*A. fumigatus* CY018, human feces, China	[82]
Secofumigaclavine E (**132**)	320	$C_{20}H_{20}N_2O_2$	*A. fumigatus* CY018, human feces, China	[82]
Fumigaclavine G (**133**)	366	$C_{23}H_{30}N_2O_2$	*A. fumigatus* HQD24, *Rhizophora stylosa* (roots), South China Sea, China	[72]
Fumigaclavine I (**134**)	382	$C_{23}H_{30}N_2O_3$	*A. fumigatus* VDL36, *Vaccinium dunalianum* (leaves), Wuding, Yunnan, China	[55]

Fumigaclavine J (**135**)	382	$C_{23}H_{30}N_2O_3$	*A. fumigatus* VDL36, *Vaccinium dunalianum* (leaves), Wuding, Yunnan, China	[55]
2-(3,3-Dimethylprop-1-ene)-costaclavine (**136**)	308	$C_{21}H_{28}N_2$	*A. fumigatus* ZTF001, *Zoanthus* sp., (zoanthid), Ayamaru Cape, Amami Island, Kagoshima Prefecture, Japan	[86]
2-(3,3-Dimethylprop-1-ene)-epicostaclavine (**137**)	308	$C_{21}H_{28}N_2$	*A. fumigatus* ZTF001, *Zoanthus* sp., (zoanthid), Ayamaru Cape, Amami Island, Kagoshima Prefecture, Japan	[86]
Costaclavine (**138**)	240	$C_{16}H_{20}N_2$	*A. fumigatus* ZTF001, *Zoanthus* sp., (zoanthid), Ayamaru Cape, Amami Island, Kagoshima Prefecture, Japan	[86]
Fumgaclavine A (**139**)	298	$C_{18}H_{22}N_2O_2$	*A. fumigatus* ZTF001, *Zoanthus* sp., (zoanthid), Ayamaru Cape, Amami Island, Kagoshima Prefecture, Japan	[86]
Fumgaclavine C (**140**)	366	$C_{23}H_{30}N_2O_2$	*A. fumigatus* ZTF001, *Zoanthus* sp., (zoanthid), Ayamaru Cape, Amami Island, Kagoshima Prefecture, Japan	[86]
Spiro-heterocyclic γ-lactam alkaloids				
Pseurotin A (**141**)	431	$C_{22}H_{25}NO_8$	*A. fumigatus* HA-57-88, soil, Sardinia	[87]
	-	-	*A. fumigatus* 030402d, marine sediment, Vanuatu	[88]
	-	-	*A. fumigatus*, soil, Kitakyushu, Japan	[89]
	-	-	*A. fumigatus* CY018, *Cynodon dactylon* (leaf), Yancheng Biosphere Reserve, Jiangsu, China	[52]
	-	-	*A. fumigatus*, soil, Rajshahi University, Bangladesh	[90]
	-	-	*A. fumigatus* LN-4, *Melia azedarach* (stem bark, Meliaceae), Yangling, Shaanxi province, China	[34]
	-	-	*A. fumigatus*, *Diphylleia sinensis* (rhizome), Honghegu, Shanxi, China	[44]
	-	-	*A. fumigatus*, *Erythrophloeum fordii* (stem), South of China	[64]
	-	-	*A. fumigatus*, *Heteroscyphus tener* (Chinese liverwort), Maoer Mountain, Guangxi Zhuang Autonomous Region, China	[45]
	-	-	*A. fumigatus*, coastal saline soil, Wudi, Shandong, China	[46]
	-	-	*A. fumigatus* MR2012, sediment, Red Sea, Hurghada, Egypt	[91]
	-	-	*A. fumigatus* KR019681, *Edgeworthia chrysantha*, coastal region of Hangzhou Bay, Hangzhou, China	[62]
	-	-	*A. fumigatus* CUGBMF170049, sea sediment, Bohai Sea, China	[92]
	-	-	*A. fumigatus* MH773172, *Ligusticum wallichii*, Dujiangyan city, Suburb of Chengdu, China	[53]
	-	-	*A. fumigatus* SD-406, cultured, deep-sea sediment, East China Sea, China	[29]
	-	-	*A. fumigatus*, *Albizia lucidior* (leaf), Zoological Garden, Giza, Egypt	[76]
	-	-	*A. fumigatus* H22, seawater, Western Pacific	[39]
	-	-	*A. fumigatus* M1, *Aconitum brevicalcaratum* (roots), Kunming, Yunnan, China	[48]
	-	-	*A. fumigatus* VDL36, *Vaccinium dunalianum* (leaves), Wuding, Yunnan, China	[55]

10*R*-15-Methylpseurotin A (**143**)	445	$C_{23}H_{27}NO_8$	*A. fumigatus* SD-406, cultured, deep-sea sediment, East China Sea, China	[29]
8-*O*-Demethylpseurotin A (**142**)	417	$C_{21}H_{23}NO_8$	*A. fumigatus* HA-57-88, soil, Sardinia	[87]
	-	-	*A. fumigatus* 030402d, marine sediment, Vanuatu	[88]
11-O-Methylpseurotin A (**144**)	445	$C_{23}H_{25}NO_8$	*A. fumigatus* MBC-F1-10/ *Streptomyces bullii* co-culture, hyper-arid Atacama Desert soil, Chile	[35]
	-	-	*A. fumigatus* MR2012, sediment, Red Sea, Hurghada, Egypt	[91]
	-	-	*A. fumigatus* GXIMD00544, Zhulin solar saltern, Beihai, China	[51]
14- Norpseurotin A (**145**)	417	$C_{21}H_{23}NO_8$	*A. fumigatus* CY018, *Cynodon dactylon* (leaf), Yancheng Biosphere Reserve, Jiangsu, China	[52]
	-	-	*A. fumigatus* SD-406, cultured, deep-sea sediment, East China Sea, China	[29]
	-	-	*A. fumigatus* H22, seawater, Western Pacific	[39]
11-Dehyroxypseurotin A (**146**)	415	$C_{22}H_{25}NO_7$	*A. fumigatus* VDL36, *Vaccinium dunalianum* (leaves), Wuding, Yunnan, China	[55]
Pseurotin A1 (**147**)	431	$C_{22}H_{25}NO_8$	*A. fumigatus* LN-4, *Melia azedarach* (stem bark, Meliaceae), Yangling, Shaanxi province, China	[34]
11-O-Methylpseurotin A2 (**148**)	445	$C_{23}H_{25}NO_8$	*A. fumigatus* MBC-F1-10/ *Streptomyces bullii* co-culture, hyper-arid Atacama Desert soil, Chile	[35]
Pseurotin A2 (**149**)	431	$C_{22}H_{25}NO_8$	*A. fumigatus* MR2012, sediment, Red Sea, Hurghada, Egypt	[91]
Pseurotin F1 (**150**)	417	$C_{21}H_{23}NO_8$	*A. fumigatus* MR2012, sediment, Red Sea, Hurghada, Egypt	[91]
	-	-	*A. fumigatus* H22, seawater, Western Pacific	[39]
	-	-	*A. fumigatus* KFQG-2/*Alternaria alternata* KFZ-32 coculture, *Coffea arabica*, Baoshan, Yunnan, China	[58]
Pseurotin F2 (**151**)	417	$C_{21}H_{23}NO_8$	*A. fumigatus* H22, seawater, Western Pacific	[39]
Pseurotin D (**152**)	431	$C_{22}H_{25}NO_8$	*A. fumigatus* MR2012, sediment, Red Sea, Hurghada, Egypt	[91]
	-	-	*A. fumigatus* UIAU-3F, soil, River Oyun in Kwara State, Nigeria	[49]
Synerazol (**153**)	413	$C_{22}H_{23}NO_7$	*A. fumigatus* SANK 10588, soil, Prachuap Khiri Khan, Thailand	[93]
Azaspirofuran A (**154**)	411	$C_{22}H_{21}NO_7$	*A. fumigatus* MR2012, sediment, Red Sea, Hurghada, Egypt	[91]
	-	-	*A. fumigatus* H22, seawater, Western Pacific	[39]
	-	-	*A. fumigatus* GXIMD00544, Zhulin solar saltern, Beihai, China	[51]
Azaspirofuran B (**155**)	397	$C_{21}H_{19}NO_7$	*A. fumigatus* MR2012, sediment, Red Sea, Hurghada, Egypt	[91]
	-	-	*A. fumigatus* H22, seawater, Western Pacific	[39]
	-	-	*A. fumigatus*, *Ceriops decandra* (leaves), Kolagachia, Sundarbans, Bangladesh	[85]
	-	-	*A. fumigatus* GXIMD00544, Zhulin solar saltern, Beihai, China	[51]
Cephalimysin A (**156**)	399	$C_{22}H_{25}NO_6$	*A. fumigatus* OUPS-T106B-5, *Mugil cephalus* (marine fish), Japan	[94]
	-	-	*A. fumigatus* MH773172, *Ligusticum wallichii*, Dujiangyan city, Suburb of Chengdu, China	[53]
Cephalimysin M (**157**)	417	$C_{22}H_{27}NO_7$	*A. fumigatus* CUGBMF170049, sea sediment, Bohai Sea, China	[92]

Cephalimysin N (**158**)	427	$C_{22}H_{21}NO_8$	*A. fumigatus* CUGBMF170049, sea sediment, Bohai Sea, China	[92]
FD-838 (**159**)	411	$C_{22}H_{21}NO_7$	*A. fumigatus* CUGBMF170049, sea sediment, Bohai Sea, China	[92]

Table S4: List of indoline, indole, amide-containing, thiodiketopiperazine, and other alkaloids isolated from *Aspergillus fumigatus*.

**Compound Name/chemical class**	**M. Wt.**	**Mol. Formula**	**Strain, Host, and Location**	**Ref.**
Indoline alkaloids				
Chaetoglobosin A (**160**)	528	$C_{32}H_{36}N_2O_5$	*A. fumigatus* AF3-093A, *Fucus vesiculosus* (brown alga), Canada	[97]
chaetoglobosin B (**161**)	528	$C_{32}H_{36}N_2O_5$	*A. fumigatus* AF3-093A, *Fucus vesiculosus* (brown alga), Canada	[97]
Fumindoline A (**162**)	382	$C_{22}H_{26}N_2O_4$	*A. fumigatus* H22, seawater, Western Pacific	[39]
Fumindoline Ba,b (**163**)	394	$C_{23}H_{26}N_2O_4$	*A. fumigatus* H22, seawater, Western Pacific	[39]
Fumindoline Ca,b (**164**)	408	$C_{24}H_{28}N_2O_4$	*A. fumigatus* H22, seawater, Western Pacific	[39]
3-Acetonylidene-7-prenylindolin-2-one (**165**)	257	$C_{16}H_{19}NO_2$	*A. fumigatus* 0338, *Nicotiana tabacum* (stem), Tengchong Prefecture, Yunnan, China	[98]
Indole alkaloids				
Asperfumigatone A (**166**)	255	$C_{16}H_{17}NO_2$	*A. fumigatus* 0338, *Nicotiana tabacum* (stem), Tengchong Prefecture, Yunnan, China	[98]
Asperfumigatone B (**167**)	269	$C_{17}H_{19}NO_2$	*A. fumigatus* 0338, *Nicotiana tabacum* (stem), Tengchong Prefecture, Yunnan, China	[98]
Asperfumigatone C (**168**)	285	$C_{17}H_{19}NO_3$	*A. fumigatus* 0338, *Nicotiana tabacum* (stem), Tengchong Prefecture, Yunnan, China	[98]
Asperfumigatone D (**169**)	257	$C_{16}H_{19}NO_2$	*A. fumigatus* 0338, *Nicotiana tabacum* (stem), Tengchong Prefecture, Yunnan, China	[98]
Asperfumigatone E (**170**)	271	$C_{17}H_{21}NO_2$	*A. fumigatus* 0338, *Nicotiana tabacum* (stem), Tengchong Prefecture, Yunnan, China	[98]
Asperfumigatone F (**171**)	268	$C_{16}H_{16}N_2O_2$	*A. fumigatus* 0338, *Nicotiana tabacum* (stem), Tengchong Prefecture, Yunnan, China	[98]
Asperfumigatone G (**172**)	298	$C_{17}H_{18}N_2O_3$	*A. fumigatus* 0338, *Nicotiana tabacum* (stem), Tengchong Prefecture, Yunnan, China	[98]
9-Methoxyolivacine (**173**)	276	$C_{18}H_{16}N_2O$	*A. fumigatus* 0338, *Nicotiana tabacum* (stem), Tengchong Prefecture, Yunnan, China	[98]
1-Acetyl-β-carboline (**174**)	210	$C_{13}H_{10}N_2O$	*A. fumigatus* HQD24, *Rhizophora mucronata* (flower), Dong Zhai Gang-Mangrove Garden, on Hainan Island, China	[47]
1-(6-Methoxy-5-prenyl-1*H*-indol-3-yl)-propan-2-one (**175**)	271	$C_{17}H_{21}NO_2$	*A. fumigatus* 0338, *Nicotiana tabacum* (stem), Tengchong Prefecture, Yunnan, China	[98]
Dilemmaone B (**176**)	215	$C_{13}H_{13}NO_2$	*A. fumigatus* 0338, *Nicotiana tabacum* (stem), Tengchong Prefecture, Yunnan, China	[98]
Bruceolline H (**177**)	229	$C_{13}H_{11}NO_3$	*A. fumigatus* 0338, *Nicotiana tabacum* (stem), Tengchong Prefecture, Yunnan, China	[98]
Psammocindole B (**178**)	282	$C_{18}H_{22}N_2O$	*A. fumigatus* 0338, *Nicotiana tabacum* (stem), Tengchong Prefecture, Yunnan, China	[98]
N-β-acetyltryptamine (**179**)	230	$C_{14}H_{18}N_2O$	*A. fumigatus*, *Erythrophloeum fordii* (stem), South of China	[64]
3-Prenyl-5-(3-keto-but-1-enyl)-indole (**180**)	253	$C_{17}H_{19}NO$	*A. fumigatus* 0338, *Nicotiana tabacum* (stem), Tengchong Prefecture, Yunnan, China	[98]
6-Methoxyindole-3-carboxylic acid *O*-*β*-D-glucopyranosyl ester (**181**)	353	$C_{16}H_{19}NO_8$	*A. fumigatus* M580, *Colochirus quadrangularis*, Co To-Thanh Lan Island, Vietnam	[54]

Amide-containing alkaloids				
N,N`- ((1*Z*,3*Z*)-1,4-Bis(4-methoxyphenyl)buta-1,3-diene-2,3-diyl)diformamide (**183**)	352	$C_{20}H_{20}N_2O_4$	*A. fumigatus/Streptomyces peucetius* 29050 co-culture, USA	[99]
N,N`-((1Z,3Z)-1,4-Bis(4-hydroxyphenyl)buta-1,3-diene-2,3-diyl)diformamide (**182**)	324	$C_{18}H_{16}N_2O_4$	*A. fumigatus/Streptomyces peucetius* 29050 co-culture, USA	[99]
N,N`-((1Z,3Z)-1-(4-Hydroxyphenyl)-4-(4-methoxyphenyl)buta-1,3-diene-2,3-diyl)diformamide (**184**)	338	$C_{19}H_{18}N_2O_4$	*A. fumigatus/Streptomyces peucetius* 29050 co-culture, USA	[99]
Fumiformamide (**185**)	418	$C_{19}H_{18}N_2O_7S$	*A. fumigatus/Streptomyces peucetius* 29050 co-culture, USA	[99]
N-Formyl-4-hydroxyphenyl-acetamide (**186**)	179	$C_9H_{93}NO_3$	*A. fumigatus*, soil, McGillivray Trail, Southeast Manitoba, Canada	[100]
N-Acetyltyramine (**187**)	179	$C_{10}H_{13}NO_2$	*A. fumigatus*, marine sediments, Jiaozhou Bay, China	[79]
N-(2-Phenylethyl)acetamide (**188**)	163	$C_{10}H_{13}NO$	*A. fumigatus* D/*Fusarium oxysporum* R1 co-culture, *Edgeworthia chrysantha*, coastal region of Hangzhou Bay, Hangzhou, China	[101]
Pyrophen (**189**)	287	$C_{16}H_{17}NO_4$	*A. fumigatus* KARSV04, Indonesia	[102]
Sphingofungin D (**190**)	431	$C_{22}H_{41}NO_7$	*A. fumigatus* MF029, *Hymeniacidon perleve* (sponge), Bohai Sea, China	[75]
Sphingofungin I (**191**)*	399	$C_{21}H_{37}NO_6$	*A. fumigatus* MF029, *Hymeniacidon perleve* (sponge), Bohai Sea, China	[75]
Sphingofungin I (**192**)*	395	$C_{22}H_{37}NO_5$	*A. fumigatus* SD-406, cultured, deep-sea sediment, East China Sea, China	[29]
11-Methyl-11-hydroxyldodecanoic acid amide (**193**)	229	$C_{13}H_{27}NO_2$	*A. fumigatus* JRJ111048, *Acrostichum specioum* (leaves), mangrove forest of Dongzhaigang, Haikou, Hainan, China	[103]
(*S*, *E*)-Methyl-2-(2,4-dimethylhex-2-enamido)acetate (**194**)	213	$C_{11}H_{19}NO_3$	*A. fumigatus* D/*Fusarium oxysporum* R1 co-culture, *Edgeworthia chrysantha*, coastal region of Hangzhou Bay, Hangzhou, China	[101]
2,5-Dioxocyclopentylamino-7-oxohepta-1,3,5-trienyl- 2,5-dihydroxy-3-chlorophenyl-2,4,6-trimethyldeca-2,4-dienamide (**195**)	568	$C_{31}H_{37}ClN_2O_6$	*A. fumigatus* MF-1, Marine soil, Bay of Bengal, Kakinada, India	[104]
Cerebroside A (**196**)	725	$C_{41}H_{75}NO_9$	*A. fumigatus* R7 culture, *Ipomoea batatas* (leaves), Egypt	[71]
Thiodiketopiperazine alkaloids				
Gliotoxin (**197**)	326	$C_{13}H_{14}N_2O_4S_2$	*A. fumigatus,* marine sediments, Jiaozhou Bay, China	[105]
	-	-	*A. fumigatus* YK-7, sea mud, intertidal zone, Yingkou, China	[33]
	-	-	*A. fumigatus* KR019681, *Edgeworthia chrysantha*, coastal region of Hangzhou Bay, Hangzhou, China	[62]
	-	-	*A. fumigatus* MH773172, *Ligusticum wallichii*, Dujiangyan city, Suburb of Chengdu, China	[53]
Spirogliotoxin (**198**)	328	$C_{13}H_{16}N_2O_4S_2$	*A. fumigatus* YK-7, sea mud, intertidal zone, Yingkou, China	[33]
Gliotoxin analogue (**199**)	280	$C_{13}H_{16}N_2O_3S$	*A. fumigatus,* marine sediments, Jiaozhou Bay, China	[105]
Didehydrobisdethiobis (methylthio) gliotoxin (**200**)	354	$C_{15}H_{18}N_2O_4S_2$	*A. fumigatus,* marine sediments, Jiaozhou Bay, China	[105]

	-	-	*A. fumigatus* YK-7, sea mud, intertidal zone, Yingkou, China	[33]
	-	-	*A. fumigatus* MH773172, *Ligusticum wallichii*, Dujiangyan city, Suburb of Chengdu, China	[53]
Bisdethiobis(methylthio)gliotoxin (**201**)	356	$C_{15}H_{20}N_2O_4S_2$	*A. fumigatus* KMM 4631, cultured, marine isolate, Russia	[31]
	-	-	*A. fumigatus* CY018, *Cynodon dactylon* (leaf), Yancheng Biosphere Reserve, Jiangsu, China	[52]
	-	-	*A. fumigatus,* marine sediments, Jiaozhou Bay, China	[105]
	-	-	*A. fumigatus* YK-7, sea mud, intertidal zone, Yingkou, China	[33]
	-	-	*A. fumigatus* LN-4, *Melia azedarach* (stem bark, Meliaceae), Yangling, Shaanxi province, China	[34]
	-	-	*A. fumigatus* R7 culture, *Ipomoea batatas* (leaves), Egypt	[71]
	-	-	*A. fumigatus* KR019681, *Edgeworthia chrysantha*, coastal region of Hangzhou Bay, Hangzhou, China	[62]
	-	-	*A. fumigatus* HQD24, *Rhizophora stylosa* (roots), South China Sea, China	[72]
	-	-	*A. fumigatus* M580, *Colochirus quadrangularis*, Co To-Thanh Lan Island, Vietnam	[54]
	-	-	*A. fumigatus* H22, seawater, Western Pacific	[39]
Gliotoxin G (**202**)	390	$C_{13}H_{14}N_2O_4S_4$	*A. fumigatus* F15, Australia	[106]
Bis-N-norgliovictin (**203**)	326	$C_{14}H_{18}N_2O_3S_2$	*A. fumigatus,* marine sediments, Jiaozhou Bay, China	[105]
Glionitrin A (**204**)	353	$C_{13}H_{11}N_3O_5S_2$	*A. fumigatus* KMC-901/*Sphingomonas sp.* KMK-001 coculture, contaminated acid mine, Young-dong abandoned coal mine, Gangneung, South Korea	[107]
Glionitrin B (**205**)	383	$C_{15}H_{17}N_3O_5S_2$	*A. fumigatus* KMC-901/*Sphingomonas sp.* KMK-001 coculture, contaminated acid mine, Young-dong abandoned coal mine, Gangneung, South Korea	[108]
Emestrin A (**206**)	598	$C_{27}H_{22}N_2O_{10}S_2$	*A. fumigatus* MBC-F1-10/ *Streptomyces bullii* co-culture, hyper-arid Atacama Desert soil, Chile	[35]
Emestrin B (**207**)	630	$C_{27}H_{22}N_2O_{10}S_3$	*A. fumigatus* MBC-F1-10/ *Streptomyces bullii* co-culture, hyper-arid Atacama Desert soil, Chile	[35]
Alternarosin A (**208**)	492	$C_{22}H_{24}N_2O_7S_2$	*A. fumigatus* SZW01, sediment, Shenzhen, Guangdong, China	[84]
Other alkaloids				
epi-Aszonalenin A (**209**)	415	$C_{25}H_{25}N_3O_3$	*A. fumigatus* SCSIO 41012, deep-sea sediments, Indian Ocean	[70]
LL-S490β (**210**)	415	$C_{25}H_{25}N_3O_3$	*A. fumigatus* culture, soil, Kitakyushu, Japan	[89]
Epothilone B (**211**)	507	$C_{27}H_{41}NO_6S$	*A. fumigatus*, *Trigonella foenum-graecum* (leaves), Zagazig, Alsharqia province, Egypt	[109]
2-Hydroxymethyl-4-methoxy-7-methylbenzo[d]thiazole-5,6-diol (**212**)	241	$C_{10}H_{11}NO_4S$	*A. fumigatus* GZWMJZ-152, deep cave soil, Fanjing Mountain of Guizhou, China	[110]
4-Methoxy-7-methylbenzo[d]thiazole-5,6-diol (**213**)	211	$C_9H_9NO_3S$	*A. fumigatus* GZWMJZ-152, deep cave soil, Fanjing Mountain of Guizhou, China	[110]
Asperfumoid (**214**)	359	$C_{18}H_{17}NO_7$	*A. fumigatus* CY018, *Cynodon dactylon* (leaf), Yancheng Biosphere Reserve, Jiangsu, China	[42]

Nicotinic acid (**215**)	123	$C_6H_5NO_2$	*A. fumigatus* HQD24, *Rhizophora mucronata* (flower), Dong Zhai Gang-Mangrove Garden on Hainan Island, China	[47]
5-Butyl-2-pyridine carboxylic acid (**216**)	179	$C_{10}H_{13}NO_2$	*A. fumigatus* nHF-01 culture, India	[111]
Pyrazoline-3-one trimer (**217**)	363	$C_{21}H_{21}N_3O_5$	*A. fumigatus* R7, *Ipomoea batatas* (leaves), Egypt	[71]
Lumichrome (**218**)	242	$C_{12}H_{10}N_4O_2$	*A. fumigatus*, *Erythrophloeum fordii* (stem), south China	[64]
2`-Deoxy-thymidine (**219**)	242	$C_{10}H_{14}N_2O_5$	*A. fumigatus* R7, *Ipomoea batatas* (leaves), Egypt	[71]
Uracil (**220**)	112	$C_4H_4N_2O_2$	*A. fumigatus* LN-4, *Melia azedarach* (stem bark), Yangling, Shaanxi province, China	[34]
(1*S*,2*R*)-2-phenyl-N-propylcyclopropanamine (**221**)	175	$C_{12}H_{17}N$	*A. fumigatus* M1, *Aconitum brevicalcaratum* (roots), Kunming, Yunnan, China	[48]
BU-4704 (**222**)	382	$C_{19}H_{14}N_2O_5S$	*A. fumigatus/Streptomyces peucetius* 29050 co-culture, USA	[99]

*Same names with different structures

Table S5: List of peptides isolated from *Aspergillus fumigatus*.

**Compound Name**	**M. Wt.**	**Mol. Formula**	**Strain, Host, and Location**	**Ref.**
Micropeptin (**223**)	1061	$C_{52}H_{75}N_{11}O_{13}$	*A. fumigatus* 3T-EGY, soil, Mansoura, Egypt	[115]
Avellanin A (**224**)	561	$C_{31}H_{39}N_5O_5$	*A. fumigatus* GXIMD 03099, *Acanthus ilicifolius*, Guangxi Shankou Mangrove Nature Reserve, China	[114]
Avellanin B (**225**)	547	$C_{30}H_{37}N_5O_5$	*A. fumigatus* GXIMD 03099, *Acanthus ilicifolius*, Guangxi Shankou Mangrove Nature Reserve, China	[114]
Avellanin D (**226**)	547	$C_{30}H_{37}N_5O_5$	*A. fumigatus* GXIMD 03099, *Acanthus ilicifolius*, Guangxi Shankou Mangrove Nature Reserve, China	[114]
Avellanin E (**227**)	575	$C_{32}H_{41}N_5O_5$	*A. fumigatus* GXIMD 03099, *Acanthus ilicifolius*, Guangxi Shankou Mangrove Nature Reserve, China	[114]
Avellanin F (**228**)	533	$C_{29}H_{35}N_5O_5$	*A. fumigatus* GXIMD 03099, *Acanthus ilicifolius*, Guangxi Shankou Mangrove Nature Reserve, China	[114]
Avellanin G (**229**)	533	$C_{29}H_{35}N_5O_5$	*A. fumigatus* GXIMD 03099, *Acanthus ilicifolius*, Guangxi Shankou Mangrove Nature Reserve, China	[114]
PF1171B (**230**)	527	$C_{28}H_{41}N_5O_5$	*A. fumigatus* GXIMD 03099, *Acanthus ilicifolius*, Guangxi Shankou Mangrove Nature Reserve, China	[114]
PF1171E (**231**)	561	$C_{31}H_{39}N_5O_5$	*A. fumigatus* GXIMD 03099, *Acanthus ilicifolius*, Guangxi Shankou Mangrove Nature Reserve, China	[114]

Table S6: List of terpenoids isolated from *Aspergillus fumigatus*.

Compound Name/chemical class	M. Wt.	Mol. Formula	Strain, Host, and Location	Ref.
Pyridino-α-pyrone sesquiterpenes				
Pyripyropene A (**232**)	583	$C_{31}H_{37}NO_{10}$	*A. fumigatus* FO-1289-2501, soil, Jingugaien, Sinjuku-Ku, Tokyo	[116]
	-	-	*A. fumigatus*, *Heteroscyphus tener* (Chinese liverwort), Maoer Mountain, Guangxi Zhuang Autonomous Region, China	[45]
	-	-	*A. fumigatus* HQD24, *Rhizophora mucronata* (flower), Dong Zhai Gang-Mangrove Garden, on Hainan Island, China	[47]
	-	-	*A. fumigatus* culture, *Crocus sativus* (lateral buds), Zhejiang, China	[38]
	-	-	*A. fumigatus* H22, seawater, Western Pacific	[39]
	-	-	*A. fumigatus* WJ-131, *Gardenia jasminoides* (stem), Kunming, Yunnan, China	[50]
	-	-	*A. fumigatus* GXIMD00544, Zhulin solar saltern, Beihai, China	[51]
	-	-	*A. fumigatus*, *Delphinium grandiflorum*, Aba Tibetan Autonomous Prefecture, China	[40]
1,11-Dideacetyl-pyripyropene A (**233**)	499	$C_{27}H_{33}NO_8$	*A. fumigatus* HQD24, *Rhizophora mucronata* (flower), Dong Zhai Gang-Mangrove Garden, on Hainan Island, China	[47]
7-Deacetylpyripyropene A (**234**)	541	$C_{29}H_{35}NO_9$	*A. fumigatus*, *Delphinium grandiflorum*, Aba Tibetan Autonomous Prefecture, China	[40]
Pyripyropene B (**235**)	597	$C_{32}H_{39}NO_{10}$	*A. fumigatus* FO-1289-2501, soil, Jingugaien, Sinjuku-Ku, Tokyo	[116]
Pyripyropene C (**236**)	597	$C_{32}H_{39}NO_{10}$	*A. fumigatus* FO-1289-2501, soil, Jingugaien, Sinjuku-Ku, Tokyo	[116]
Pyripyropene D (**237**)	597	$C_{32}H_{39}NO_{10}$	*A. fumigatus* FO-1289-2501, soil, Jingugaien, Sinjuku-Ku, Tokyo	[116]
Pyripyropene E (**238**)	451	$C_{27}H_{33}NO_5$	*A. fumigatus* FO-1289-2501, soil, Jingugaien, Sinjuku-Ku, Tokyo	[116]
	-	-	*A. fumigatus* HQD24, *Rhizophora mucronata* (flower), Dong Zhai Gang-Mangrove Garden, Hainan Island, China	[47]
Pyripyropene F (**239**)	465	$C_{28}H_{35}NO_5$	*A. fumigatus* FO-1289-2501, soil, Jingugaien, Sinjuku-Ku, Tokyo	[116]
Pyripyropene G (**240**)	467	$C_{27}H_{33}NO_6$	*A. fumigatus* FO-1289-2501, soil, Jingugaien, Sinjuku-Ku, Tokyo	[116]
Pyripyropene H (**241**)	481	$C_{28}H_{35}NO_6$	*A. fumigatus* FO-1289-2501, soil, Jingugaien, Sinjuku-Ku, Tokyo	[116]
Pyripyropene I (**242**)	625	$C_{34}H_{43}NO_{10}$	*A. fumigatus* FO-1289-2501, soil, Jingugaien, Sinjuku-Ku, Tokyo	[116]
Pyripyropene J (**243**)	611	$C_{33}H_{41}NO_{10}$	*A. fumigatus* FO-1289-2501, soil, Jingugaien, Sinjuku-Ku, Tokyo	[116]
Pyripyropene K (**244**)	611	$C_{33}H_{41}NO_{10}$	*A. fumigatus* FO-1289-2501, soil, Jingugaien, Sinjuku-Ku, Tokyo	[116]
Pyripyropene L (**245**)	611	$C_{33}H_{41}NO_{10}$	*A. fumigatus* FO-1289-2501, soil, Jingugaien, Sinjuku-Ku, Tokyo	[116]
Pyripyropene M (**246**)	581	$C_{32}H_{39}NO_9$	*A. fumigatus* FO-1289-2501, soil, Jingugaien, Sinjuku-Ku, Tokyo	[116]

Pyripyropene N (**247**)	553	$C_{31}H_{39}NO_8$	*A. fumigatus* FO-1289-2501, soil, Jingugaien, Sinjuku-Ku, Tokyo	[116]
Pyripyropene O (**248**)	509	$C_{29}H_{35}NO_7$	*A. fumigatus* FO-1289-2501, soil, Jingugaien, Sinjuku-Ku, Tokyo	[116]
Pyripyropene P (**249**)	523	$C_{30}H_{37}NO_7$	*A. fumigatus* FO-1289-2501, soil, Jingugaien, Sinjuku-Ku, Tokyo	[116]
Pyripyropene Q (**250**)	539	$C_{30}H_{37}NO_8$	*A. fumigatus* FO-1289-2501, soil, Jingugaien, Sinjuku-Ku, Tokyo	[116]
Pyripyropene R (**251**)	523	$C_{30}H_{37}NO_7$	*A. fumigatus* FO-1289-2501, soil, Jingugaien, Sinjuku-Ku, Tokyo	[116]
GERI-BP001 A (**252**)	453	$C_{27}H_{35}NO_5$	*A. fumigatus* F37, soil, Mountain Dukyou, Cheonbuk, Korea	[122]
GERI-BP001 B (**253**)	451	$C_{28}H_{37}NO_4$	*A. fumigatus* F37, soil, Mountain Dukyou, Cheonbuk, Korea	[122,123]
GERI-BP001 M (**254**)	437	$C_{27}H_{35}NO_4$	*A. fumigatus* F37, soil, Mountain Dukyou, Cheonbuk, Korea	[122,123]
Sesquiterpenoids				
Fumagillin (**255**)	458	$C_{26}H_{34}O_7$	*A. fumigatus,* cultured, USA	[124]
	-	-	*A. fumigatus* CANU A151, saline lake sand, Western Australia	[41]
	-	-	*A. fumigatus* H22, seawater, Western Pacific	[39]
Fumagiringillin (**256**)	476	$C_{26}H_{36}O_8$	*A. fumigatus* CANU A151, saline lake sand, Western Australia	[41]
	-	-	*A. fumigatus* H22, seawater, Western Pacific	[39]
Sch 528647 (**257**)	442	$C_{26}H_{34}O_6$	*A. fumigatus,* cultured, USA	[124]
RK-95113 (**258**)	402	$C_{24}H_{34}O_5$	*A. fumigatus* RK95-113 (JCM 12527, FERM P-19233), soil, Wako, Saitama, Japan	[125]
Fumagillol (**259**)	282	$C_{16}H_{26}O_4$	*A. fumigatus* H-3, cultured, Japan	[126]
	-	-	*A. fumigatus* RK95-113 (JCM 12527, FERM P-19233), soil, Wako, Saitama, Japan	[125]
5-Demethoxyfumagillol (**260**)	252	$C_{15}H_{24}O_3$	*A. fumigatus* IMI-069714, cultured, Korea	[127]
Tricho-9-ene-2α,3α,11α,16-tetraol (**261**)	282	$C_{15}H_{24}O_5$	*A. fumigatus* R7 culture, *Ipomoea batatas* (leaves), Egypt	[71]
Aspergiketone (**262**)	250	$C_{15}H_{22}O_3$	*A. fumigatus*, coastal saline soil, Wudi, Shandong, China	[46]
Fumagillene A (**263**)	236	$C_{15}H_{24}O_2$	*A. fumigatus* MH773172, *Ligusticum wallichii*, Dujiangyan city, Suburb of Chengdu, China	[53]
Fumagillene B (**264**)	236	$C_{15}H_{24}O_2$	*A. fumigatus* MH773172, *Ligusticum wallichii*, Dujiangyan city, Suburb of Chengdu, China	[53]
Fumigatanol (**265**)	270	$C_{15}H_{26}O_4$	*A. fumigatus* M1, *Aconitum brevicalcaratum* (roots), Kunming, Yunnan, China	[48]
Xylariterpenoid H (**266**)	270	$C_{15}H_{26}O_4$	*A. fumigatus*, *Delphinium grandiflorum*, Aba Tibetan Autonomous Prefecture, China	[40]
E-β-*trans*-5,8,11-trihydroxybergamot-9-ene (**267**)	252	$C_{15}H_{24}O_3$	*A. fumigatus* M1, *Aconitum brevicalcaratum* (roots), Kunming, Yunnan, China	[48]
Fumiparaphine A (**268**)	236	$C_{15}H_{24}O_2$	*A. fumigatus* YXG-12-2/*Paraphaeosphaeria* sp. YXG-18 coculture, *Ginkgo biloba*, Yunnan University, Yunnan, China	[128]
Fumiparaphine B (**269**)	266	$C_{16}H_{26}O_3$	*A. fumigatus* YXG-12-2/*Paraphaeosphaeria* sp. YXG-18 coculture, *Ginkgo biloba*, Yunnan University, Yunnan, China	[128]
Fumiparaphine C (**270**)	266	$C_{16}H_{26}O_3$	*A. fumigatus* YXG-12-2/*Paraphaeosphaeria* sp. YXG-18 coculture, *Ginkgo biloba*, Yunnan University, Yunnan, China	[128]

Fuminoid A (**271**)	252	$C_{15}H_{24}O_3$	*A. fumigatus*, Wuhan, China	[56]
Fumigatonin (**272**)	562	$C_{29}H_{38}O_{11}$	*A. fumigatus* IFM4482, cultured, Japan	[129]
Triterpenoids				
Helvolic acid (**273**)	568	$C_{33}H_{44}O_8$	*A. fumigatus* CY018, *Cynodon dactylon* (leaf), Yancheng Biosphere Reserve, Jiangsu, China	[42]
	-	-	*A. fumigatus* LN-4, *Melia azedarach* (stem bark, Meliaceae), Yangling, Shaanxi province, China	[34]
	-	-	*A. fumigatus*, *Diphylleia sinensis* (rhizome), Honghegu, Shanxi, China	[44]
	-	-	*A. fumigatus* HNMF0047, unidentified sponge (HNMF00), Wenchang beach, Hainan, China	[130]
	-	-	*A. fumigatus* SCSIO 41012, deep-sea sediments, Indian Ocean	[70]
	-	-	*A. fumigatus* CUGBMF170049, sea sediment, Bohai Sea, China	[92]
	-	-	*A. fumigatus*, *Albizia lucidior* (leaf), Zoological Garden, Giza, Egypt	[76]
	-	-	*A. fumigatus* culture, *Crocus sativus* (lateral buds), Zhejiang, China	[38]
	-	-	*A. fumigatus* H22, seawater, Western Pacific	[39]
	-	-	*A. fumigatus* WJ-131, *Gardenia jasminoides* (stem), Kunming, Yunnan, China	[50]
	-	-	*A. fumigatus*, *Delphinium grandiflorum*, Aba Tibetan Autonomous Prefecture, China	[40]
Helvolic acid methyl ester (**274**)	582	$C_{34}H_{46}O_8$	*A. fumigatus* WJ-131, *Gardenia jasminoides* (stem), Kunming, Yunnan, China	[50]
1,2-Dihydrohelvolic acid (**275**)	570	$C_{33}H_{46}O_8$	*A. fumigatus* CUGBMF170049, sea sediment, Bohai Sea, China	[92]
6,16-O-Dideacetylhelvolic acid 21,16-lactone (**276**)	466	$C_{29}H_{38}O_5$	*A. fumigatus* HNMF0047, unidentified sponge (HNMF00), Wenchang beach, Hainan, China	[130]
16-O-Deacetylhelvolic acid 21,16-lactone (**277**)	508	$C_{31}H_{40}O_6$	*A. fumigatus* HNMF0047, unidentified sponge (HNMF00), Wenchang beach, Hainan, China	[130]
	-	-	*A. fumigatus* HQD24, *Rhizophora stylosa* (roots), South China Sea, China	[72]
6-O-Propionyl-6,16-O-dideacetylhelvolic acid 21,16-lactone (**278**)	522	$C_{32}H_{42}O_6$	*A. fumigatus* HNMF0047, unidentified sponge (HNMF00), Wenchang beach, Hainan, China	[130]
	-	-	*A. fumigatus* H22, seawater, Western Pacific	[39]
1,2-Dihydro-6,16-O-dideacetylhelvolic acid 21,16-lactone (**279**)	468	$C_{29}H_{40}O_5$	*A. fumigatus* HNMF0047, unidentified sponge (HNMF00), Wenchang beach, Hainan, China	[130]
1,2-Dihydro-16-O-deacetylhelvolic acid 21,16-lactone (**280**)	510	$C_{31}H_{42}O_6$	*A. fumigatus* HNMF0047, unidentified sponge (HNMF00), Wenchang beach, Hainan, China	[130]
16-O-Propionyl-16-O-deacetylhelvolic acid (**281**)	582	$C_{34}H_{46}O_8$	*A. fumigatus* HNMF0047, unidentified sponge (HNMF00), Wenchang beach, Hainan, China	[130]
	-	-	*A. fumigatus* CUGBMF170049, sea sediment, Bohai Sea, China	[92]
	-	-	*A. fumigatus* H22, seawater, Western Pacific	[39]
6-O-Propionyl-6-O-deacetylhelvolic acid (**282**)	582	$C_{34}H_{46}O_8$	*A. fumigatus* HNMF0047, unidentified sponge (HNMF00), Wenchang beach, Hainan, China	[130]
	-	-	*A. fumigatus* CUGBMF170049, sea sediment, Bohai Sea, China	[92]
Asperfumin A (**283**)	582	$C_{34}H_{46}O_8$	*A. fumigatus* HNMF0047, *Cleidion brevipetiolatum* (root), Mengla Country Yunnan, China	[95]

Asperfumin B (**284**)	582	$C_{34}H_{46}O_8$	*A. fumigatus* HNMF0047*, Cleidion brevipetiolatum* (root), Mengla Country Yunnan, China	[95]
6β,16β-Diacetoxy-25-hydroxy-3,7-dioxo-29-nordammara-1,17(20)-dien-21,24-lactone (**285**)	584	$C_{33}H_{44}O_9$	*A. fumigatus* HNMF0047*,* unidentified sponge (HNMF00), Wenchang beach, Hainan, China	[130]
24-*epi*-6β,16β-Diacetoxy-25-hydroxy-3,7-dioxo-29-nordammara-1,17(20)-diene-21,24-lactone (**286**)	584	$C_{33}H_{44}O_9$	*A. fumigatus* HNMF0047*,* unidentified sponge (HNMF00), Wenchang beach, Hainan, China	[130]
4,8,10,14-Tetramethyl-6-acetoxy-14-[16-acetoxy-19-(20,21-dimethyl)-18-ene]-phenanthrene-1-ene-3,7-dione (**287**)	486	$C_{29}H_{42}O_6$	*A. fumigatus*, *Diphylleia sinensis* (rhizome), Honghegu, Shanxi, China	[44]
Fumihopaside A (**288**)	652	$C_{36}H_{60}O_{10}$	*A. fumigatus* CEA17.1, cultured, China	[131]
Fumihopaside B (**289**)	634	$C_{36}H_{58}O_9$	*A. fumigatus* CEA17.1, cultured, China	[131]
(S)-2-hydroxy-2-((3*S*,3a*S*,5a*R*,5b*R*,7a*R*,8*S*,9*S*,11a*R*,11b*R*,13a*R*,13b*S*)-9-hydroxy-8-(hydroxymethyl)-5a,5b,8,11a,13b-pentamethylicosahydro-1H-cyclopenta[a]chrysen-3-yl)propanoic acid (**290**)	490	$C_{30}H_{50}O_5$	*A. fumigatus* CEA17.1, cultured, China	[131]
21*β*H-hopane-3*β*,22-diol (**291**)	444	$C_{30}H_{52}O_2$	*A. fumigatus* CEA17.1, cultured, China	[131]
1,4,23-Trihydroxy-hopane-22,30-diol (**292**)	490	$C_{30}H_{52}O_5$	*A. fumigatus* SD-406, cultured, deep-sea sediment, East China Sea, China	[29]

Table S7: List of sterols isolated from *Aspergillus fumigatus*.

**Compound Name**	**M. Wt.**	**Mol. Formula**	**Strain, Host, and Location**	**Ref.**
3,7-Diketo-cephalosporin P1 (**293**)	528	$C_{31}H_{44}O_7$	*A. fumigatus* SCSIO 41012, deep-sea sediments, Indian Ocean	[70]
22-*O*-Acetylisocyclocitrinol A (**294**)	442	$C_{27}H_{38}O_5$	*A. fumigatus* SCSIO 41012, deep-sea sediments, Indian Ocean	[70]
Ergosterol (**295**)	396	$C_{28}H_{44}O$	*A. fumigatus* J-4, Japan	[132]
	-	-	*A. fumigatus* CY018, *Cynodon dactylon* (leaf), Yancheng Biosphere Reserve, Jiangsu, China	[42]
	-	-	*A. fumigatus* MBC-F1-10/ *Streptomyces bullii* co-culture, hyper-arid Atacama Desert soil, Chile	[35]
	-	-	*A. fumigatus* AR05, *Astragalus membranaceus* (root), Hengshan Mountains, Shanxi Province, China	[36]
	-	-	*A. fumigatus*, *Albizia lucidior* (leaf), Zoological Garden, Giza, Egypt	[76]
	-	-	*A. fumigatus* WJ-131*, Gardenia jasminoides* (stem), Kunming, Yunnan, China	[50]
(3*β*,22*E*,24*R*)-Stigmasta-5,7,22-trien-3-*β*-ol (**296**)	410	$C_{29}H_{46}O$	*A. fumigatus* WJ-131*, Gardenia jasminoides* (stem), Kunming, Yunnan, China	[50]

5α,8α-Epidioxy-ergosta-6,22-diene-3β-ol (**297**)	428	$C_{28}H_{44}O_3$	*A. fumigatus* CY018, *Cynodon dactylon* (leaf), Yancheng Biosphere Reserve, Jiangsu, China	[42]
	-	-	*A. fumigatus* AR05, *Astragalus membranaceus* (root), Hengshan Mountains, Shanxi Province, China	[36]
	-	-	*A. fumigatus*, *Diphylleia sinensis* (rhizome), Honghegu, Shanxi, China	[44]
	-	-	*A. fumigatus*, *Albizia lucidior* (leaf), Zoological Garden, Giza, Egypt	[76]
Isofucosterol (**298**)	412	$C_{29}H_{48}O$	*A. fumigatus* WJ-131, *Gardenia jasminoides* (stem), Kunming, Yunnan, China	[50]
Ergosta- 4,22-diene-3β-ol (**299**)	398	$C_{28}H_{46}O$	*A. fumigatus* CY018, *Cynodon dactylon* (leaf), Yancheng Biosphere Reserve, Jiangsu, China	[42]
Cerevisterol (**300**)	430	$C_{28}H_{46}O_3$	*A. fumigatus* AR05, *Astragalus membranaceus* (root), Hengshan Mountains, Shanxi Province, China	[36]
β-Sistosterol linoleate (**301**)	1074	$C_{75}H_{126}O_3$	*A. fumigatus* M1, *Aconitum brevicalcaratum* (roots), Kunming, Yunnan, China	[48]

Table S8: List of quinone, anthraquinone, and anthracenone derivatives isolated from *Aspergillus fumigatus*.

**Compound Name/chemical class**	**M. Wt.**	**Mol. Formula**	**Strain, Host, and Location**	**Ref.**
Fumigatin (**302**)	168	$C_8H_8O_4$	*A. fumigatus* DH 413, cultured, Osaka, Japan	[135]
Fumigatin quinol (**303**)	170	$C_8H_{10}O_4$	*A. fumigatus* DH 413, cultured, Osaka, Japan	[135]
Fumigatin epioxide (**304**)	184	$C_8H_8O_5$	*A. fumigatus* DH 413, cultured, Osaka, Japan	[135]
Fumigatin chlorohydrin (**305**)	220	$C_8H_9ClO_5$	*A. fumigatus* DH 413, cultured, Osaka, Japan	[136]
Spinulosin (**306**)	184	$C_8H_8O_5$	*A. fumigatus* DH 413, cultured, Osaka, Japan	[135]
	-	-	*A. fumigatus*, soil, Kitakyushu, Japan	[89]
Spinulosin quinol (**307**)	186	$C_8H_{10}O_5$	*A. fumigatus* DH 413, cultured, Osaka, Japan	[136]
Dihydrospinulosin quinol (**308**)	188	$C_8H_{12}O_5$	*A. fumigatus* DH 413, cultured, Osaka, Japan	[136]
Spinulosin hydrate (**309**)	202	$C_8H_{10}O_6$	*A. fumigatus* DH 413, cultured, Osaka, Japan	[136]
Spinulosin quinol-hydrate (**310**)	204	$C_8H_{12}O_6$	*A. fumigatus* DH 413, cultured, Osaka, Japan	[136]
3,4-Dihydroxytoluquinone (**311**)	154	$C_7H_6O_4$	*A. fumigatus* DH 413, cultured, Osaka, Japan	[134]
3-Hydroxy-5-methoxy-2-methylbenzoquinone (**312**)	168	$C_8H_8O_4$	*A. fumigatus* GZWMJZ-152, deep cave soil, Fanjing Mountain of Guizhou, China	[110]
Fumiquinone A (**313**)	254	$C_{12}H_{14}O_6$	*A. fumigatus*, soil, Kitakyushu, Japan	[89]
Fumiquinone B (**314**)	184	$C_8H_8O_5$	*A. fumigatus*, soil, Kitakyushu, Japan	[89]
Emodin (**315**)	270	$C_{15}H_{10}O_5$	*A. fumigatus* J-4, Japan	[132]

	-	-	*A. fumigatus* MF029, *Hymeniacidon perleve* (sponge), Bohai Sea, China	[75]
	-	-	*A. fumigatus* culture, *Crocus sativus* (lateral buds), Zhejiang, China	[38]
1-Methyl emodin (**316**)	284	$C_{16}H_{12}O_5$	*A. fumigatus*, *Delphinium grandiflorum*, Aba Tibetan Autonomous Prefecture, China	[40]
Emodin 1,6-dimethyl ether (**317**)	298	$C_{17}H_{14}O_5$	*A. fumigatus*, *Diphylleia sinensis* (rhizome), Honghegu, Shanxi, China	[44]
Isorhodoptilometrin (**318**)	314	$C_{17}H_{14}O_6$	*A. fumigatus*, *Diphylleia sinensis* (rhizome), Honghegu, Shanxi, China	[44]
(+)-2`*S*-Isorhodoptilometrin (**319**)	314	$C_{17}H_{14}O_6$	*A. fumigatus* H22, seawater, Western Pacific	[39]
Questin (**320**)	286	$C_{15}H_{10}O_6$	*A. fumigatus* NRRL 35693, cultured, France	[13]
	-	-	*A. fumigatus*, *Heteroscyphus tener* (Chinese liverwort), Maoer Mountain, Guangxi Zhuang Autonomous Region, China	[45]
	-	-	*A. fumigatus* HQD24, *Rhizophora stylosa* (roots), South China Sea, China	[72]
	-	-	*A. fumigatus* culture, *Crocus sativus* (lateral buds), Zhejiang, China	[38]
	-	-	*A. fumigatus* H22, seawater, Western Pacific	[39]
	-	-	*A. fumigatus* WJ-131, *Gardenia jasminoides* (stem), Kunming, Yunnan, China	[50]
Fragilin (**321**)	318	$C_{16}H_{11}ClO_5$	*A. fumigatus* J-4, Japan	[132]
2-Chloro-1,3,8-trihydroxy-6-methylanthrone (**322**)	290	$C_{15}H_{11}ClO_4$	*A. fumigatus* J-4, Japan	[132]
2-Bromo-1,3,8-trihydroxy-6-methylanthrone (**323**)	333	$C_{15}H_{11}BrO_4$	*A. fumigatus* J-4, Japan	[132]
2-Chloro-1,3,8-trihydroxy-6-methylanthraquinone (**324**)	304	$C_{15}H_9ClO_5$	*A. fumigatus* J-4, Japan	[132]
2-Bromo-1,3,8-trihydroxy-6-methylanthraquinone (**325**)	347	$C_{15}H_9BrO_5$	*A. fumigatus* J-4, Japan	[132]
2-Chloro-1,3,8-trihydroxy-6-hydroxymethylanthraquinone (**326**)	320	$C_{15}H_9ClO_6$	*A. fumigatus* J-4, Japan	[132]
Physcion (**327**)	284	$C_{16}H_{12}O_5$	*A. fumigatus* CY018, *Cynodon dactylon* (leaf), Yancheng Biosphere Reserve, Jiangsu, China	[42]
	-	-	*A. fumigatus* 3T-EGY, soil, Mansoura, Egypt	[115]
	-	-	*A. fumigatus* HQD24, *Rhizophora stylosa* (roots), South China Sea, China	[72]
Neosartoricin (**328**)	484	$C_{26}H_{28}O_9$	*A. fumigatus/Neosartorya fischeri* coculture, USA	[137]
2,4,5,17-Tetramethoxy pradimicin lactone (**329**)	542	$C_{30}H_{22}O_{10}$	*A. fumigatus* 3T-EGY, soil, Mansoura, Egypt	[115]
	-	-	*A. fumigatus* R7, *Ipomoea batatas* (leaves), Egypt	[71]
	-	-	*A. fumigatus* HQD24, *Rhizophora stylosa* (roots), South China Sea, China	[72]
Juglanthraquinone A-5-*O*-D-rhodosamine- (4`→1``)-2-deoxy-D-glucose (4``→1```)-cinerulose B (**330**)	753	$C_{39}H_{47}NO_{14}$	*A. fumigatus* 3T-EGY, soil, Mansoura, Egypt	[115]

Table S9: List of benzophenones and diphenyl ethers isolated from *Aspergillus fumigatus*.

Compound Name/chemical class	M. Wt.	Mol. Formula	Strain, Host, and Location	Ref.
Benzophenones				
Monomethylsulochrin-4-sulphate (**331**)	426	$C_{18}H_{18}O_{10}S$	*A. fumigatus*, *Albizia lucidior* (leaf), Zoological Garden, Giza, Egypt	[76]
Sulfurasperine A (**332**)	504	$C_{23}H_{24}N_2O_9S$	*A. fumigatus* GZWMJZ-152, deep cave soil, Fanjing Mountain of Guizhou, China	[110]
Sulfurasperine B ((±)-**333**)	408	$C_{19}H_{20}O_8S$	*A. fumigatus* GZWMJZ-152, deep cave soil, Fanjing Mountain of Guizhou, China	[110]
Sulfurasperine C ((±)-**334**)	434	$C_{21}H_{22}O_8S$	*A. fumigatus* GZWMJZ-152, deep cave soil, Fanjing Mountain of Guizhou, China	[110]
Sulfurasperine D (**335**)	450	$C_{21}H_{22}O_9S$	*A. fumigatus* GZWMJZ-152, deep cave soil, Fanjing Mountain of Guizhou, China	[110]
Sulochrin (**336**)	332	$C_{17}H_{16}O_7$	*A. fumigatus*, *Heteroscyphus tener* (Chinese liverwort), Maoer Mountain, Guangxi Zhuang Autonomous Region, China	[45]
	-	-	*A. fumigatus* HQD24, *Rhizophora stylosa* (roots), South China Sea, China	[72]
	-	-	*A. fumigatus* GZWMJZ-152, deep cave soil, Fanjing Mountain of Guizhou, China	[110]
	-	-	*A. fumigatus* H22, seawater, Western Pacific	[39]
Monomethylsulochrin (**337**)	346	$C_{18}H_{18}O_7$	*A. fumigatus* CY018, *Cynodon dactylon* (leaf), Yancheng Biosphere Reserve, Jiangsu, China	[42]
	-	-	*A. fumigatus* NRRL 35693, cultured, France	[13]
	-	-	*A. fumigatus*, *Diphylleia sinensis* (rhizome), Honghegu, Shanxi, China	[44]
	-	-	*A. fumigatus* HQD24, *Rhizophora stylosa* (roots), South China Sea, China	[72]
	-	-	*A. fumigatus*, *Albizia lucidior* (leaf), Zoological Garden, Giza, Egypt	[76]
	-	-	*A. fumigatus* GZWMJZ-152, deep cave soil, Fanjing Mountain of Guizhou, China	[110]
	-	-	*A. fumigatus* culture, *Crocus sativus* (lateral buds), Zhejiang, China	[38]
	-	-	*A. fumigatus* H22, seawater, Western Pacific	[39]
	-	-	*A. fumigatus* WJ-131, *Gardenia jasminoides* (stem), Kunming, Yunnan, China	[50]
	-	-	*A. fumigatus*, *Delphinium grandiflorum*, Aba Tibetan Autonomous Prefecture, China	[40]
8\`-Hydroxymonomethylsulochrin (**338**)	362	$C_{18}H_{18}O_8$	*A. fumigatus* WJ-131, *Gardenia jasminoides* (stem), Kunming, Yunnan, China	[50]
5-Hydroxy-2-(2-hydroxy-6-methoxy-4-methylbenzoyl)-3-methoxybenzoic acid (**339**)	346	$C_{18}H_{18}O_7$	*A. fumigatus* WJ-131, *Gardenia jasminoides* (stem), Kunming, Yunnan, China	[50]
Asperfumin (**340**)	376	$C_{19}H_{20}O_8$	*A. fumigatus* CY018, *Cynodon dactylon* (leaf), Yancheng Biosphere Reserve, Jiangsu, China	[42]
Rhizoctonic acid (**341**)	332	$C_{17}H_{16}O_7$	*A. fumigatus* HQD24, *Rhizophora stylosa* (roots), South China Sea, China	[72]
2,6\`-Dihydroxy-2,4\`-dimethoxy-8\`- methyl-6-methoxy-acyl-ethyl-diphenylmethanone (**342**)	360	$C_{19}H_{20}O_7$	*A. fumigatus* SZW01, sediment, Shenzhen, Guangdong, China	[84]
Penibenzophenone E (**343**)	332	$C_{17}H_{16}O_7$	*A. fumigatus* H22, seawater, Western Pacific	[39]
Diphenyl ethers				

8`-*O*-Methylasterric acid (**344**)	362	$C_{18}H_{18}O_8$	*A. fumigatus*, *Heteroscyphus tener* (Chinese liverwort), Maoer Mountain, Guangxi Zhuang Autonomous Region, China	[45]
	-	-	*A. fumigatus* H22, seawater, Western Pacific	[39]
Dimethyl 2,3`-dimethylosoate (**345**)	376	$C_{19}H_{20}O_8$	*A. fumigatus* H22, seawater, Western Pacific	[39]
4-Carboxy-5,5`-dihydroxy-3,3`-dimethyl-diphenylether (**346**)	274	$C_{15}H_{14}O_5$	*A. fumigatus* DH 413, cultured, Osaka, Japan	[133]

Table S10: List of chromane and isochromane derivatives isolated from *Aspergillus fumigatus*.

**Compound Name/chemical class**	**M. Wt.**	**Mol. Formula**	**Strain, Host, and Location**	**Ref.**
Chromane derivatives				
Ruakuric acid (**347**)	266	$C_{13}H_{14}O_6$	*A. fumigatus,* a coral lichen, hot, sulfurous springs environment, Craters-of-the-Moon, North Island of New Zealand	[139]
Ruakuric acid methyl ester (**348**)	280	$C_{14}H_{16}O_6$	*A. fumigatus,* a coral lichen, hot, sulfurous springs environment, Craters-of-the-Moon, North Island of New Zealand	[139]
3,5-Dihydroxy-7-*O*-α-rhamnopyranoyl-2H-chromen-2-one (**349**)	340	$C_{15}H_{16}O_9$	*A. fumigatus* 3T-EGY, soil, Mansoura, Egypt	[115]
Rubrofusarin B (**350**)	286	$C_{16}H_{14}O_5$	*A. fumigatus* KR019681, *Edgeworthia chrysantha*, coastal region of Hangzhou Bay, Hangzhou, China	[140]
Asperpyrone A (**351**)	556	$C_{31}H_{24}O_{10}$	*A. fumigatus* KR019681, *Edgeworthia chrysantha*, coastal region of Hangzhou Bay, Hangzhou, China	[140]
Asperpyrone D (**352**)	556	$C_{31}H_{24}O_{10}$	*A. fumigatus* KR019681, *Edgeworthia chrysantha*, coastal region of Hangzhou Bay, Hangzhou, China	[140]
Fonsecinone A (**353**)	570	$C_{32}H_{26}O_{10}$	*A. fumigatus* KR019681, *Edgeworthia chrysantha*, coastal region of Hangzhou Bay, Hangzhou, China	[140]
Fonsecinone B (**354**)	588	$C_{32}H_{28}O_{11}$	*A. fumigatus* KR019681, *Edgeworthia chrysantha*, coastal region of Hangzhou Bay, Hangzhou, China	[140]
Fonsecinone D (**355**)	588	$C_{32}H_{28}O_{11}$	*A. fumigatus* KR019681, *Edgeworthia chrysantha*, coastal region of Hangzhou Bay, Hangzhou, China	[140]
Aurasperone A (**356**)	570	$C_{32}H_{26}O_{10}$	*A. fumigatus* KR019681, *Edgeworthia chrysantha*, coastal region of Hangzhou Bay, Hangzhou, China	[140]
Isochromane derivatives				
1-(8-Methoxy-3-methyl-1H-isochromen-6-yl)propan-1-one (**357**)	232	$C_{14}H_{16}O_3$	*A. fumigatus* YATAS-22-042, *Nicotiana tabacum* (leaves), Gengma, Lincang, Yunnan, China	[141]
3-Hydroxy-1-(8- methoxy-3-methyl-1H-isochromen-7-yl)propan-1-one (**358**)	248	$C_{14}H_{16}O_4$	*A. fumigatus* YATAS-22-042, *Nicotiana tabacum* (leaves), Gengma, Lincang, Yunnan, China	[141]

(5-Methoxy-7-methyl-1H-isochromen-3-yl)methanol (**359**)	206	$C_{12}H_{14}O_3$	*A. fumigatus* YATAS-22-042, *Nicotiana tabacum* (leaves), Gengma, Lincang, Yunnan, China	[141]
7-Isopropyl-5-methoxy-3-methyl-1H-isochromene (**360**)	218	$C_{14}H_{18}O_2$	*A. fumigatus* YATAS-22-042, *Nicotiana tabacum* (leaves), Gengma, Lincang, Yunnan, China	[141]
(7-Isopropyl-5-methoxy-1H-isochromen-3-yl)methanol (**361**)	234	$C_{14}H_{18}O_3$	*A. fumigatus* YATAS-22-042, *Nicotiana tabacum* (leaves), Gengma, Lincang, Yunnan, China	[141]
Versicolol B (**362**)	244	$C_{16}H_{20}O_2$	*A. fumigatus* YATAS-22-042, *Nicotiana tabacum* (leaves), Gengma, Lincang, Yunnan, China	[141]
Oryzaein D (**363**)	276	$C_{16}H_{20}O_4$	*A. fumigatus* YATAS-22-042, *Nicotiana tabacum* (leaves), Gengma, Lincang, Yunnan, China	[141]
6-Hydroxy mellein (**364**)	194	$C_{10}H_{10}O_4$	*A. fumigatus*, *Bacopa monnieri*, Lucknow, India	[142]
6,8-dihydroxy-5-methoxy-3-methyl-1H-isochromen- 1-one (**365**)	224	$C_{10}H_8O_6$	*A. fumigatus* HQD24, *Rhizophora stylosa* (roots), South China Sea, China	[72]
6-Hydroxy-8-methoxy-3-methylisocoumarin (**366**)	206	$C_{11}H_{10}O_4$	*A. fumigatus* culture, *Crocus sativus* (lateral buds), Zhejiang, China	[38]
Fraxetin (**367**)	208	$C_{10}H_8O_5$	*A. fumigatus*, *Ceriops decandra* (leaves), Kolagachia, Sundarbans, Bangladesh	[85]
Alternariol 9-*O*-methyl ether (**368**)	272	$C_{15}H_{12}O_5$	*A. fumigatus* KR019681, *Edgeworthia chrysantha*, coastal region of Hangzhou Bay, Hangzhou, China	[140]
(-)-Viriditoxin (**369**)	662	$C_{34}H_{30}O_{14}$	*A. fumigatus* 1863, soil, Himalaya	[143]

Table S11: List of other metabolites isolated from *Aspergillus fumigatus*.

**Compound Name/chemical class**	**M. Wt.**	**Mol. Formula**	**Strain, Host, and Location**	**Ref.**
Azaphilone derivatives				
Pinophilin B (**370**)	402	$C_{21}H_{22}O_8$	*A. fumigatus* 14–27, *Carijoa* sp., (gorgonian), South China Sea, China	[144]
epi-Pinophilin B (**371**)	402	$C_{21}H_{22}O_8$	*A. fumigatus* 14–27, *Carijoa* sp., (gorgonian), South China Sea, China	[144]
Pinazaphilone B (**372**)	416	$C_{21}H_{20}O_9$	*A. fumigatus* 14–27, *Carijoa* sp., (gorgonian), South China Sea, China	[144]
Pinophilin E (**373**)	404	$C_{21}H_{24}O_8$	*A. fumigatus* 14–27, *Carijoa* sp., (gorgonian), South China Sea, China	[144]
Anhydrides				
spergide (**374**)	332	$C_{18}H_{20}O_6$	*A. fumigatus* JRJ111048, *Acrostichum specioum* (leaves), mangrove forest of Dongzhaigang, Haikou, Hainan, China,	[103]
Spiculisporic acid B (**375**)	326	$C_{17}H_{26}O_6$	*A. fumigatus* JRJ111048, *Acrostichum specioum* (leaves), mangrove forest of Dongzhaigang, Haikou, Hainan, China,	[103]
seco-Spiculisporic acid B (**376**)	360	$C_{18}H_{32}O_7$	*A. fumigatus* JRJ111048, *Acrostichum specioum* (leaves), mangrove forest of Dongzhaigang, Haikou, Hainan, China,	[103]
Spiculisporic acid C (**377**)	342	$C_{18}H_{30}O_6$	*A. fumigatus* JRJ111048, *Acrostichum specioum* (leaves), mangrove forest of Dongzhaigang, Haikou, Hainan, China,	[103]

Spiculisporic acid (**378**)	328	$C_{17}H_{28}O_6$	*A. fumigatus* JRJ111048, *Acrostichum specioum* (leaves), mangrove forest of Dongzhaigang, Haikou, Hainan, China,	[103]
Pyranones				
Dehydromevalonic lactone (**379**)	130	$C_6H_{10}O_3$	*A. fumigatus* WA7S6*,* marine sponge, Red Sea, Hurghada, Egypt	[145]
Neovasinin (**380**)	308	$C_{17}H_{24}O_5$	*A. fumigatus* D/*Fusarium oxysporum* R1 co-culture, *Edgeworthia chrysantha*, coastal region of Hangzhou Bay, Hangzhou, China	[101]
Cyclohexanones				
Asperfumtone A (**381**)	186	$C_8H_{10}O_5$	*A. fumigatus* KFQG-2/*Alternaria alternata* KFZ-32 coculture, *Coffea arabica*, Baoshan, Yunnan, China	[58]
Asperfumtone B (**382**)	184	$C_8H_8O_5$	*A. fumigatus* KFQG-2/*Alternaria alternata* KFZ-32 coculture, *Coffea arabica*, Baoshan, Yunnan, China	[58]
Naphthalene derivatives				
4,8-Dihydroxy-1-tetralone (**383**)	178	$C_{10}H_{10}O_3$	*A. fumigatus* LN-4, *Melia azedarach* (stem bark, Meliaceae), Yangling, Shaanxi province, China	[34]
	-	-	*A. fumigatus*, *Albizia lucidior* (leaf), Zoological Garden, Giza, Egypt	[76]
trans-3,4-Dihydro-3,4,8-trihydroxynaphthalen-1(2H)-one (**384**)	194	$C_{10}H_{10}O_4$	*A. fumigatus* LN-4, *Melia azedarach* (stem bark, Meliaceae), Yangling, Shaanxi province, China	[34]
cis-3,4-Dihydro-3,4,8-trihydroxynaphthalen-1(2H)-one (**385**)	194	$C_{10}H_{10}O_4$	*A. fumigatus* LN-4, *Melia azedarach* (stem bark, Meliaceae), Yangling, Shaanxi province, China	[34]
4,6-Dihydroxy, 3,8a-dimethyl-1-oxo-5-(3`-oxobutan-2`-yl)-1,4,4a,5,6,8a-hexahydronaphthalen-2-yl-1``,2``-dimethyl-5``-(2```-methylprop-1```-enyl)cyclopentanecarboxylate (**386**)	472	$C_{28}H_{40}O_6$	*A. fumigatus*, *Garcinia griffithii* (fruits), Sarasah Bonta, Lembah Arau, Kabupaten Lima Puluh Kota, West Sumatra, Indonesia	[146]
Furan-containing				
Trypacidin (**387**)	344	$C_{18}H_{16}O_7$	*A. fumigatus* NRRL 35693, cultured, France	[13]
	-	-	*A. fumigatus*, *Diphylleia sinensis* (rhizome), Honghegu, Shanxi, China	[44]
	-	-	*A. fumigatus*, *Heteroscyphus tener* (Chinese liverwort), Maoer Mountain, Guangxi Zhuang Autonomous Region, China	[45]
	-	-	*A. fumigatus* HQD24, *Rhizophora stylosa* (roots), South China Sea, China	[72]
	-	-	*A. fumigatus* MF029, *Hymeniacidon perleve* (sponge), Bohai Sea, China	[75]
	-	-	*A. fumigatus* H22, seawater, Western Pacific	[39]
	-	-	*A. fumigatus* WJ-131*, Gardenia jasminoides* (stem), Kunming, Yunnan, China	[50]
Neovasifuranone B (**388**)	282	$C_{16}H_{26}O_4$	*A. fumigatus* D/*Fusarium oxysporum* R1 co-culture, *Edgeworthia chrysantha*, coastal region of Hangzhou Bay, Hangzhou, China	[101]
2-Furoic acid (**389**)	112	$C_5H_4O_3$	*A. fumigatus* 1T-2, Yuanye, Shanghai, China	[147]
Aldehyde				
Flavipin (**390**)	196	$C_9H_8O_5$	*A. fumigatus* AF3-093A, *Fucus vesiculosus* (brown alga), Canada	[97]
Phenolics				

2,4-Dihydroxy-3-methylacetophenone (**391**)	166	$C_9H_{10}O_3$	*A. fumigatus* IFM 54246, soil, Brazil	[68]
Orcinol (**392**)	124	$C_7H_8O_2$	*A. fumigatus* M1, *Aconitum brevicalcaratum* (roots), Kunming, Yunnan, China	[48]
Atraric acid (**393**)	196	$C_{10}H_{12}O_4$	*A. fumigatus*, soil, McGillivray Trail, Southeast Manitoba, Canada	[100]
Di-(2-Ethylhexyl) phthalate (**394**)	390	$C_{24}H_{38}O_4$	*A. fumigatus* 3T-EGY, soil, Mansoura, Egypt	[115]
α-Ethyl glucoside (**395**)	208	$C_8H_{16}O_6$	*A. fumigatus* JRJ111048, *Acrostichum specioum* (leaves), mangrove forest of Dongzhaigang, Haikou, Hainan, China,	[103]
Fatty acids and related derivatives				
Fumifungin (**396**)	431	$C_{22}H_{41}NO_7$	*A. fumigatus* 1863, soil, Himalaya	[143]
Linoleic acid (**397**)	280	$C_{18}H_{32}O_2$	*A. fumigatus* R7, *Ipomoea batatas* (leaves), Egypt	[71]
α-Linolenic acid (**398**)	278	$C_{18}H_{30}O_2$	*A. fumigatus* 3T-EGY, soil, Mansoura, Egypt	[115]
	-	-	*A. fumigatus* D/*Fusarium oxysporum* R1 co-culture, *Edgeworthia chrysantha*, coastal region of Hangzhou Bay, Hangzhou, China	[101]
9-Tetradecynoic acid (**399**)	242	$C_{14}H_{24}O_2$	*A. fumigatus* WA7S6*,* marine sponge, Red Sea, Hurghada, Egypt	[145]
11-Hexadecynoic acid methyl ester (**400**)	266	$C_{17}H_{30}O_2$	*A. fumigatus* WA7S6*,* marine sponge, Red Sea, Hurghada, Egypt	[145]
R(-)-Glycerol monolinoleate (**401**)	254	$C_{21}H_{38}O_4$	*A. fumigatus* R7, *Ipomoea batatas* (leaves), Egypt	[71]
Stearic acid (**402**)	284	$C_{18}H_{36}O_2$	*A. fumigatus* 3T-EGY, soil, Mansoura, Egypt	[115]
α-Elaeostearic acid (**403**)	278	$C_{18}H_{30}O_2$	*A. fumigatus* D/*Fusarium oxysporum* R1 co-culture, *Edgeworthia chrysantha*, coastal region of Hangzhou Bay, Hangzhou, China	[101]
Palmitoleic acid (**404**)	254	$C_{22}H_{42}O_4$	*A. fumigatus* D/*Fusarium oxysporum* R1 co-culture, *Edgeworthia chrysantha*, coastal region of Hangzhou Bay, Hangzhou, China	[101]
10-Methyl-9*Z*-octadecenoic glyceride (**405**)	370	$C_{16}H_{30}O_2$	*A. fumigatus* culture*, Crocus sativus* (lateral buds), Zhejiang, China	[38]

Table S13. Biological activities of reported metabolites from *Aspergillus fumigatus*.

Compound name	Biological activity	Assay, Organism or Cell Line	Biological Results		Ref.
			Compound	Positive control	
Fumitremorgin B (**3**)	Antifungal	Broth microdilution/*Botrytis cinerea*	6.25 µg/mL (MIC)	Carbendazim, 12.5 µg/mL (MIC)	[34]
		Broth microdilution/*Alternaria solani*	6.25 µg/mL (MIC)	Carbendazim, 12.5 µg/mL (MIC)	[34]
		Broth microdilution/*Alternaria alternata* (Fries) Keissler	6.25 µg/mL (MIC)	Carbendazim, 6.25 µg/mL (MIC)	[34]
		Broth microdilution/ *Colletotrichum gloeosporioides*	12.5 µg/mL (MIC)	Carbendazim, 6.25 µg/mL (MIC)	[34]
		Broth microdilution/*Fusarium oxysporum* f. sp. *niveum*	25.0 µg/mL (MIC)	Carbendazim, 12.5 µg/mL (MIC)	[34]
		Broth microdilution/*Gibberella saubinettii.*	12.5 µg/mL (MIC)	Carbendazim, 6.25 µg/mL (MIC)	[34]
Spirotryprostatin G = 6-methoxyspirotryprostatin B (**5**)	Antifungal	Broth microdilution/*C. albicans*	0.39 µg/mL (MIC)	Amphotericin B, 0.39 µg/mL (MIC)	[62]
Derivative A-fumitremorgin B (**6**)	Cytotoxicity	MTT/HL-60	3.4 µM (IC_{50})	VP16, 0.083 µM (IC_{50})	[32]
		SRB/A-549	11.0 µM (IC_{50})	VP16, 1.40 µM (IC_{50})	[32]
		SRB/BEL-7402	7.0 µM (IC_{50})	VP16, 1.025 µM (IC_{50})	[32]
Derivative A epimer-fumitremorgin B (**7**)	Cytotoxicity	MTT/HL-60	5.4 µM (IC_{50})	VP16, 0.083 µM (IC_{50})	[32]
		SRB/A-549	11.60 µM (IC_{50})	VP16, 1.40 µM (IC_{50})	[32]
		SRB/BEL-7402	10.80 µM (IC_{50})	VP16, 1.025 µM (IC_{50})	[32]
Fumitremorgin C (**8**)	Antifungal	Broth microdilution/*T. rubrum*	62.5 µg/mL (MIC)	Ketonazole, 31.5 µg/mL (MIC)	[42]
		Broth microdilution/*Botrytis cinerea*	12.5 µg/mL (MIC)	Carbendazim, 12.5 µg/mL (MIC)	[34]
		Broth microdilution/*Alternaria solani*	25.0 µg/mL (MIC)	Carbendazim, 12.5 µg/mL (MIC)	[34]
		Broth microdilution/*Alternaria alternata* (Fries) Keissler	12.5 µg/mL (MIC)	Carbendazim, 6.25 µg/mL (MIC)	[34]
		Broth microdilution/	12.5 µg/mL (MIC)	Carbendazim, 6.25 µg/mL (MIC)	[34]

		Colletotrichum gloeosporioides			
		Broth microdilution/*Gibberella saubinettii.*	12.5 µg/mL (MIC)	Carbendazim, 6.25 µg/mL (MIC)	[34]
12α,13α-Dihydroxyfumitremorgin C (**10**)	Cytotoxicity	MTT/U937	1.8 µM (IC_{50})	Doxorubicin HCl, 0.021 µM (IC_{50})	[33]
		MTT/PC-3	6.6 µM (IC_{50})	Doxorubicin HCl, 0.73 µM (IC_{50})	[33]
12β-Hydroxy-13-oxofumitremorgin C (**14**)	Antifungal	Broth microdilution/*Fusarium oxysporum*	15.62 µg/mL (MIC)	Ketoconazole, 15.62 µg/mL (MIC)	[55]
		Broth microdilution/*Coriolus versicolor*	15.62 µg/mL (MIC)	Ketoconazole, 7.80 µg/mL (MIC)	[55]
		Broth microdilution/*Fusarium solani*	31.25 µg/mL (MIC)	Ketoconazole, 31.25 µg/mL (MIC)	[55]
		Broth microdilution/*Botrytis cinerea*	31.25 µg/mL (MIC)	Ketoconazole, 15.62 µg/mL (MIC)	[55]
		Broth microdilution/*Fusarium graminearum*	15.62 µg/mL (MIC)	Ketoconazole, 15.62 µg/mL (MIC)	[55]
13-Ethoxycyclotryprostatin A (**25**)	Antifungal	Broth microdilution/*Fusarium oxysporum*	7.80 µg/mL (MIC)	Ketoconazole, 15.62 µg/mL (MIC)	[55]
		Broth microdilution/*Coriolus versicolor*	7.80 µg/mL (MIC)	Ketoconazole, 7.80 µg/mL (MIC)	[55]
		Broth microdilution/*Fusarium solani*	15.62 µg/mL (MIC)	Ketoconazole, 31.25 µg/mL (MIC)	[55]
		Broth microdilution/*Botrytis cinerea*	15.62 µg/mL (MIC)	Ketoconazole, 15.62 µg/mL (MIC)	[55]
		Broth microdilution/*Fusarium graminearum*	31.25 µg/mL (MIC)	Ketoconazole, 15.62 µg/mL (MIC)	[55]
13-Dehydroxycyclotryprostatin A (**26**)	Antifungal	Broth microdilution/*Fusarium oxysporum*	15.62 µg/mL (MIC)	Ketoconazole, 15.62 µg/mL (MIC)	[55]
		Broth microdilution/*Coriolus versicolor*	15.62 µg/mL (MIC)	Ketoconazole, 7.80 µg/mL (MIC)	[55]
		Broth microdilution/*Fusarium solani*	31.25 µg/mL (MIC)	Ketoconazole, 31.25 µg/mL (MIC)	[55]
		Broth microdilution/*Botrytis cinerea*	31.25 µg/mL (MIC)	Ketoconazole, 15.62 µg/mL (MIC)	[55]

		Broth microdilution/*Fusarium graminearum*	31.25 µg/mL (MIC)	Ketoconazole, 15.62 µg/mL (MIC)	[55]
Cyclotryprostatin B (**29**)	Antifungal	Broth microdilution/*Botrytis cinerea*	12.5 µg/mL (MIC)	Carbendazim, 12.5 µg/mL (MIC)	[34]
		Broth microdilution/*Alternaria solani*	25.0 µg/mL (MIC)	Carbendazim, 12.5 µg/mL (MIC)	[34]
		Broth microdilution/*Alternaria alternata* (Fries) Keissler	12.5 µg/mL (MIC)	Carbendazim, 6.25 µg/mL (MIC)	[34]
		Broth microdilution/ *Colletotrichum gloeosporioides*	12.5 µg/mL (MIC)	Carbendazim, 6.25 µg/mL (MIC)	[34]
		Broth microdilution/*Fusarium solani*	50.0 µg/mL (MIC)	Carbendazim, 25.0 µg/mL (MIC)	[34]
		Broth microdilution/*Fusarium oxysporum* f. sp. *niveum*	25.0 µg/mL (MIC)	Carbendazim, 12.5 µg/mL (MIC)	[34]
		Broth microdilution/*Fusarium oxysporum* f. sp. *vasinfectum*	50.0 µg/mL (MIC)	Carbendazim, 25.0 µg/mL (MIC)	[34]
		Broth microdilution/*Gibberella saubinettii.*	12.5 µg/mL (MIC)	Carbendazim, 6.25 µg/mL (MIC)	[34]
	Antibacterial	Broth microdilution/*Gibberella saubinettii.*	1.0 µg/mL (MIC)	Gentamicin, 0.5 µg/mL (MIC)	[36]
		Broth microdilution/*Bacillus subtilis*	1.0 µg/mL (MIC)	Gentamicin, 1.0 µg/mL (MIC)	[36]
		Broth microdilution/*Staphylococcus aureus*	1.0 µg/mL (MIC)	Gentamicin, 0.5 µg/mL (MIC)	[36]
		Broth microdilution/*Escherichia coli*	2.0 µg/mL (MIC)	Gentamicin, 0.5 µg/mL (MIC)	[36]
Verruculogen (**37**)	Antifungal	Broth microdilution/*Botrytis cinerea*	6.25 µg/mL (MIC)	Carbendazim, 12.5 µg/mL (MIC)	[34]
		Broth microdilution/*Alternaria solani*	12.5 µg/mL (MIC)	Carbendazim, 12.5 µg/mL (MIC)	[34]
		Broth microdilution/*Alternaria alternata* (Fries) Keissler	6.25 µg/mL (MIC)	Carbendazim, 6.25 µg/mL (MIC)	[34]
		Broth microdilution/	6.25 µg/mL (MIC)	Carbendazim, 6.25 µg/mL (MIC)	[34]

		Colletotrichum gloeosporioides			
		Broth microdilution/*Fusarium solani*	50.0 μg/mL (MIC)	Carbendazim, 25.0 μg/mL (MIC)	[34]
		Broth microdilution/*Fusarium oxysporum* f. sp. *niveum*	12.5 μg/mL (MIC)	Carbendazim, 12.5 μg/mL (MIC)	[34]
		Broth microdilution/*Fusarium oxysporum* f. sp. *vasinfectum*	25.0 μg/mL (MIC)	Carbendazim, 25.0 μg/mL (MIC)	[34]
		Broth microdilution/*Gibberella saubinettii.*	6.25 μg/mL (MIC)	Carbendazim, 6.25 μg/mL (MIC)	[34]
	Antibacterial	Broth microdilution/*Bacillus subtilis*	2.0 μg/mL (MIC)	Gentamicin, 0.5 μg/mL (MIC)	[36]
		Broth microdilution/*Staphylococcus aureus*	4.0 μg/mL (MIC)	Gentamicin, 1.0 μg/mL (MIC)	[36]
		Broth microdilution/*Escherichia coli*	2.0 μg/mL (MIC)	Gentamicin, 0.5 μg/mL (MIC)	[36]
		Broth microdilution/*Salmonella typhimurium*	2.0 μg/mL (MIC)	Gentamicin, 0.5 μg/mL (MIC)	[36]
13-Oxoverruculogen (**38**)	Cytotoxicity	MTT/HL-60	1.90 μM (IC_{50})	VP16, 0.083 μM (IC_{50})	[32]
		SRB/A-549	16.9 μM (IC_{50})	VP16, 1.40 μM (IC_{50})	[32]
		SRB/BEL-7402	25.6 μM (IC_{50})	VP16, 1.025 μM (IC_{50})	[32]
Verruculogen TR-2 (**39**)	Antifungal	Broth microdilution/*Botrytis cinerea*	12.5 μg/mL (MIC)	Carbendazim, 12.5 μg/mL (MIC)	[34]
		Broth microdilution/*Alternaria solani*	12.5 μg/mL (MIC)	Carbendazim, 12.5 μg/mL (MIC)	[34]
		Broth microdilution/*Alternaria alternata* (Fries) Keissler	6.25 μg/mL (MIC)	Carbendazim, 6.25 μg/mL (MIC)	[34]
		Broth microdilution/ *Colletotrichum gloeosporioides*	12.5 μg/mL (MIC)	Carbendazim, 6.25 μg/mL (MIC)	[34]
		Broth microdilution/*Fusarium solani*	25.0 μg/mL (MIC)	Carbendazim, 25.0 μg/mL (MIC)	[34]
		Broth microdilution/*Fusarium oxysporum* f. sp. *niveum*	12.5 μg/mL (MIC)	Carbendazim, 12.5 μg/mL (MIC)	[34]

		Broth microdilution/*Fusarium oxysporum* f. sp. *vasinfectum*	25.0 µg/mL (MIC)	Carbendazim, 25.0 µg/mL (MIC)	[34]
		Broth microdilution/*Gibberella saubinettii.*	12.5 µg/mL (MIC)	Carbendazim, 6.25 µg/mL (MIC)	[34]
12β-Hydroxyverruculogen TR-2 (**40**)	Antifungal	Broth microdilution/*Botrytis cinerea*	25.0 µg/mL (MIC)	Carbendazim, 12.5 µg/mL (MIC)	[34]
		Broth microdilution/*Alternaria solani*	25.0 µg/mL (MIC)	Carbendazim, 12.5 µg/mL (MIC)	[34]
		Broth microdilution/*Alternaria alternata* (Fries) Keissler	25.0 µg/mL (MIC)	Carbendazim, 6.25 µg/mL (MIC)	[34]
		Broth microdilution/ *Colletotrichum gloeosporioides*	25.0 µg/mL (MIC)	Carbendazim, 6.25 µg/mL (MIC)	[34]
		Broth microdilution/*Fusarium solani*	50.0 µg/mL (MIC)	Carbendazim, 25.0 µg/mL (MIC)	[34]
		Broth microdilution/*Fusarium oxysporum* f. sp. *niveum*	25.0 µg/mL (MIC)	Carbendazim, 12.5 µg/mL (MIC)	[34]
		Broth microdilution/*Fusarium oxysporum* f. sp. *vasinfectum*	50.0 µg/mL (MIC)	Carbendazim, 25.0 µg/mL (MIC)	[34]
		Broth microdilution/*Gibberella saubinettii.*	25.0 µg/mL (MIC)	Carbendazim, 6.25 µg/mL (MIC)	[34]
12β-Hydroxy-13α-methoxyverruculogen TR-2 (**41**)	Antifungal	Broth microdilution/*Botrytis cinerea*	6.25 µg/mL (MIC)	Carbendazim, 12.5 µg/mL (MIC)	[34]
		Broth microdilution/*Alternaria solani*	6.25 µg/mL (MIC)	Carbendazim, 12.5 µg/mL (MIC)	[34]
		Broth microdilution/*Alternaria alternata* (Fries) Keissler	6.25 µg/mL (MIC)	Carbendazim, 6.25 µg/mL (MIC)	[34]
		Broth microdilution/ *Colletotrichum gloeosporioides*	6.25 µg/mL (MIC)	Carbendazim, 6.25 µg/mL (MIC)	[34]
		Broth microdilution/*Fusarium solani*	25.0 µg/mL (MIC)	Carbendazim, 25.0 µg/mL (MIC)	[34]
		Broth microdilution/*Fusarium oxysporum* f. sp. *niveum*	12.5 µg/mL (MIC)	Carbendazim, 12.5 µg/mL (MIC)	[34]
		Broth microdilution/*Fusarium oxysporum* f. sp. *vasinfectum*	25.0 µg/mL (MIC)	Carbendazim, 25.0 µg/mL (MIC)	[34]

		Broth microdilution/*Gibberella saubinettii.*	6.25 µg/mL (MIC)	Carbendazim, 6.25 µg/mL (MIC)	[34]
Spirotryprostatin A (**42**)	Antibacterial	Broth microdilution/*E. coli*	0.39 µg/mL (MIC)	Ampicillin, 0.39 µg/mL (MIC)	[62]
		Broth microdilution/*S. aureus*	0.39 µg/mL (MIC)	Ampicillin, 0.39 µg/mL (MIC)	[62]
	Antifungal	Broth microdilution/*C. albicans*	0.78 µg/mL (MIC)	Amphotericin B, 0.39 µg/mL (MIC)	[62]
Spirotryprostatin E (**49**)	Cytotoxicity	MTT/MOLT-4	3.1 µM (IC_{50})	VP16, 0.003 µM (IC_{50})	[32]
		MTT/HL-60	2.3 µM (IC_{50})	VP16, 0.083 µM (IC_{50})	[32]
		SRB/A-549	3.1 µM (IC_{50})	VP16, 1.40 µM (IC_{50})	[32]
Tryptoquivaline O (**67**)	Antifungal	Broth microdilution/*Botrytis cinerea*	25.0 µg/mL (MIC)	Carbendazim, 12.5 µg/mL (MIC)	[34]
		Broth microdilution/*Alternaria solani*	12.5 µg/mL (MIC)	Carbendazim, 12.5 µg/mL (MIC)	[34]
		Broth microdilution/*Alternaria alternata* (Fries) Keissler	25.0 µg/mL (MIC)	Carbendazim, 6.25 µg/mL (MIC)	[34]
Fumiquinazoline A (**68**)	Antifungal	Broth microdilution/*Botrytis cinerea*	12.5 µg/mL (MIC)	Carbendazim, 12.5 µg/mL (MIC)	[34]
		Broth microdilution/*Alternaria solani*	12.5 µg/mL (MIC)	Carbendazim, 12.5 µg/mL (MIC)	[34]
		Broth microdilution/*Alternaria alternata* (Fries) Keissler	12.5 µg/mL (MIC)	Carbendazim, 6.25 µg/mL (MIC)	[34]
		Broth microdilution/ *Colletotrichum gloeosporioides*	12.5 µg/mL (MIC)	Carbendazim, 6.25 µg/mL (MIC)	[34]
		Broth microdilution/*Gibberella saubinettii.*	12.5 µg/mL (MIC)	Carbendazim, 6.25 µg/mL (MIC)	[34]
3-Hydroxyfumiquinazoline A (**69**)	Antifungal	Broth microdilution/*Botrytis cinerea*	12.5 µg/mL (MIC)	Carbendazim, 12.5 µg/mL (MIC)	[34]
		Broth microdilution/*Alternaria solani*	12.5 µg/mL (MIC)	Carbendazim, 12.5 µg/mL (MIC)	[34]
		Broth microdilution/*Alternaria alternata* (Fries) Keissler	12.5 µg/mL (MIC)	Carbendazim, 6.25 µg/mL (MIC)	[34]
		Broth microdilution/ *Colletotrichum gloeosporioides*	12.5 µg/mL (MIC)	Carbendazim, 6.25 µg/mL (MIC)	[34]

		Broth microdilution/*Gibberella saubinettii.*	12.5 µg/mL (MIC)	Carbendazim, 6.25 µg/mL (MIC)	[34]
Fumiquinazoline C (**71**)	Antibacterial	Broth microdilution/*S. aureus* (ATCC 16339)	1.565 µg/mL (MIC)	Streptomycin, 6.25 µg/mL (MIC)	[70]
		Broth microdilution/ *S. aureus* (ATCC 29213)	0.78 µg/mL (MIC)	Streptomycin, 3.125 µg/mL (MIC)	[70]
Fumiquinazoline D (**73**)	Antifungal	Broth microdilution/*Botrytis cinerea*	25.0 µg/mL (MIC)	Carbendazim, 12.5 µg/mL (MIC)	[34]
		Broth microdilution/*Alternaria solani*	25.0 µg/mL (MIC)	Carbendazim, 12.5 µg/mL (MIC)	[34]
		Broth microdilution/*Alternaria alternata* (Fries) Keissler	25.0 µg/mL (MIC)	Carbendazim, 6.25 µg/mL (MIC)	[34]
		Broth microdilution/ *Colletotrichum gloeosporioides*	25.0 µg/mL (MIC)	Carbendazim, 6.25 µg/mL (MIC)	[34]
		Broth microdilution/*Gibberella saubinettii.*	25.0 µg/mL (MIC)	Carbendazim, 6.25 µg/mL (MIC)	[34]
Fumiquinazoline F (**74**)	Antifungal	Broth microdilution/*Botrytis cinerea*	12.5 µg/mL (MIC)	Carbendazim, 12.5 µg/mL (MIC)	[34]
		Broth microdilution/*Alternaria solani*	25.0 µg/mL (MIC)	Carbendazim, 12.5 µg/mL (MIC)	[34]
		Broth microdilution/*Alternaria alternata* (Fries) Keissler	12.5 µg/mL (MIC)	Carbendazim, 6.25 µg/mL (MIC)	[34]
		Broth microdilution/ *Colletotrichum gloeosporioides*	12.5 µg/mL (MIC)	Carbendazim, 6.25 µg/mL (MIC)	[34]
		Broth microdilution/*Gibberella saubinettii.*	12.5 µg/mL (MIC)	Carbendazim, 6.25 µg/mL (MIC)	[34]
Fumiquinazoline G (**75**)	Antifungal	Broth microdilution/*Botrytis cinerea*	12.5 µg/mL (MIC)	Carbendazim, 12.5 µg/mL (MIC)	[34]
		Broth microdilution/*Alternaria solani*	25.0 µg/mL (MIC)	Carbendazim, 12.5 µg/mL (MIC)	[34]
		Broth microdilution/*Alternaria alternata* (Fries) Keissler	12.5 µg/mL (MIC)	Carbendazim, 6.25 µg/mL (MIC)	[34]
		Broth microdilution/ *Colletotrichum gloeosporioides*	12.5 µg/mL (MIC)	Carbendazim, 6.25 µg/mL (MIC)	[34]

		Broth microdilution/*Gibberella saubinettii.*	12.5 µg/mL (MIC)	Carbendazim, 6.25 µg/mL (MIC)	[34]
	Antibacterial	Broth microdilution/*A. baumanii* (ATCC 15122)	6.25 µg/mL (MIC)	Streptomycin, 12.5 µg/mL (MIC)	[70]
		Broth microdilution/*S. aureus* (ATCC 16339)	12.5 µg/mL (MIC)	Streptomycin, 6.25 µg/mL (MIC)	[70]
Fumiquinazoline J (**76**)	Antibacterial	Broth microdilution/*Gibberella saubinettii.*	1.0 µg/mL (MIC)	Gentamicin, 0.5 µg/mL (MIC)	[36]
		Broth microdilution/*Bacillus subtilis*	1.0 µg/mL (MIC)	Gentamicin, 1.0 µg/mL (MIC)	[36]
		Broth microdilution/*Staphylococcus aureus*	1.0 µg/mL (MIC)	Gentamicin, 0.5 µg/mL (MIC)	[36]
		Broth microdilution/*Escherichia coli*	1.0 µg/mL (MIC)	Gentamicin, 0.5 µg/mL (MIC)	[36]
	Antifungal	Broth microdilution/*Candida albicans*	2.0 µg/mL (MIC)	Nystatin, 4.0 µg/mL (MIC)	[36]
		Broth microdilution/*Penicillium chrysogenum*	4.0 µg/mL (MIC)	Nystatin, 4.0 µg/mL (MIC)	[36]
		Broth microdilution/*Fusarium solani*	2.0 µg/mL (MIC)	Nystatin, 8.0 µg/mL (MIC)	[36]
Fumigatosides E (**82**)	Antibacterial	Broth microdilution/*A. baumanii* (ATCC 15122)	6.25 µg/mL (MIC)	Streptomycin, 12.5 µg/mL (MIC)	[70]
		Broth microdilution/*S. aureus* (ATCC 16339)	6.25 µg/mL (MIC)	Streptomycin, 6.25 µg/mL (MIC)	[70]
Chaetominine (**88**)	Antifungal	Broth microdilution/*Fusarium oxysporum*	31.25 µg/mL (MIC)	Ketoconazole, 15.62 µg/mL (MIC)	[55]
Secofumigaclavine B (**124**)	Anti-inflammatory	Griess method/ LPS-induced NO production in RAW264.7 cells	3.48 µM (IC_{50})	Indomethacin, 10.99 µM (IC_{50})	[82]
Fumigaclavine C (**125**)	Antifungal	Broth microdilution/*T. rubrum*	31.5 µg/mL (MIC),	Ketonazole, 31.5 µg/mL (MIC)	[42]
9-Deacetoxyfumigaclavine C (**128**)	Cytotoxicity	MTT/ K562	3.1 µM (IC_{50})	Doxorubicin, 1.2 µM (IC_{50})	[52]
Secofumigaclavine D (**130**)	Anti-inflammatory	Griess method/ LPS-induced NO production in RAW264.7 cells	7.83 µM (IC_{50})	Indomethacin, 10.99 µM (IC_{50})	[82]

Secofumigaclavine E (**131**)	Anti-inflammatory	Griess method/ LPS-induced NO production in RAW264.7 cells	9.47 µM (IC_{50})	Indomethacin, 10.99 µM (IC_{50})	[82]
Fumigaclavine I (**134**)	Antifungal	Broth microdilution/*Fusarium oxysporum*	15.62 µg/mL (MIC)	Ketoconazole, 15.62 µg/mL (MIC)	[55]
		Broth microdilution/*Coriolus versicolor*	15.62 µg/mL (MIC)	Ketoconazole, 7.80 µg/mL (MIC)	[55]
		Broth microdilution/*Fusarium solani*	62.50 µg/mL (MIC)	Ketoconazole, 31.25 µg/mL (MIC)	[55]
		Broth microdilution/*Botrytis cinerea*	15.62 µg/mL (MIC)	Ketoconazole, 15.62 µg/mL (MIC)	[55]
		Broth microdilution/*Fusarium graminearum*	31.25 µg/mL (MIC)	Ketoconazole, 15.62 µg/mL (MIC)	[55]
Fumigaclavine J (**135**)	Antifungal	Broth microdilution/*Fusarium oxysporum*	31.25 µg/mL (MIC)	Ketoconazole, 15.62 µg/mL (MIC)	[55]
Pseurotin A (**141**)	Antibacterial	Broth microdilution/*E. coli*	0.78 µg/mL (MIC)	Ampicillin, 0.39 µg/mL (MIC)	[62]
		Broth microdilution/*S. aureus*	0.39 µg/mL (MIC)	Ampicillin, 0.39 µg/mL (MIC)	[62]
Gliotoxin (**197**)	Cytotoxicity	MTT/U937	0.20 µM (IC_{50})	Doxorubicin HCl, 0.021 µM (IC_{50})	[33]
		MTT/PC-3	0.39 µM (IC_{50})	Doxorubicin HCl, 0.73 µM (IC_{50})	[33]
	Antibacterial	Broth microdilution/*E. coli*	0.78 µg/mL (MIC)	Ampicillin, 0.39 µg/mL (MIC)	[62]
Bisdethiobis(methylthio)gliotoxin (**201**)	Cytotoxicity	MTT/U937	0.52 µM (IC_{50})	Doxorubicin HCl, 0.021 µM (IC_{50})	[33]
	Antifungal	Broth microdilution/*C. albicans*	0.39 µg/mL (MIC)	Amphotericin B, 0.39 µg/mL (MIC)	[62]
epi-Aszonalenin A (**209**)	Antibacterial	Broth microdilution/*A. baumanii* (ATCC 15122)	6.25 µg/mL (MIC)	Streptomycin, 12.5 µg/mL (MIC)	[70]
Pyripyropene A (**232**)	ACAT inhibitor	Radioactive using enzyme rate liver microsomes	0.16 µM (IC_{50})	CL-283,546, 1.3 µM (IC_{50})	[116]
Pyripyropene B (**235**)	ACAT inhibitor	Radioactive using enzyme rate liver microsomes	0.32 µM (IC_{50})	CL-283,546, 1.3 µM (IC_{50})	[116]
Pyripyropene C (**236**)	ACAT inhibitor	Radioactive using enzyme rate liver microsomes	0.15 µM (IC_{50})	CL-283,546, 1.3 µM (IC_{50})	[116]
Pyripyropene D (**237**)	ACAT inhibitor	Radioactive using enzyme rate liver microsomes	0.268 µM (IC_{50})	CL-283,546, 1.3 µM (IC_{50})	[119]

Pyripyropene I (**242**)	ACAT inhibitor	Radioactive using enzyme rate liver microsomes	2.45 µM (IC_{50})	CL-283,546, 1.3 µM (IC_{50})	[117]
Pyripyropene J (**243**)	ACAT inhibitor	Radioactive using enzyme rate liver microsomes	0.85 µM (IC_{50})	CL-283,546, 1.3 µM (IC_{50})	[117]
Pyripyropene K (**244**)	ACAT inhibitor	Radioactive using enzyme rate liver microsomes	2.65 µM (IC_{50})	CL-283,546, 1.3 µM (IC_{50})	[117]
Pyripyropene L (**245**)	ACAT inhibitor	Radioactive using enzyme rate liver microsomes	0.27 µM (IC_{50})	CL-283,546, 1.3 µM (IC_{50})	[116]
Pyripyropene M (**246**)	ACAT inhibitor	Radioactive using enzyme rate liver microsomes	3.80 µM (IC_{50})	CL-283,546, 1.3 µM (IC_{50})	[116]
Pyripyropene N (**247**)	ACAT inhibitor	Radioactive using enzyme rate liver microsomes	48.00 µM (IC_{50})	CL-283,546, 1.3 µM (IC_{50})	[116]
Pyripyropene O (**248**)	ACAT inhibitor	Radioactive using enzyme rate liver microsomes	11.00 µM (IC_{50})	CL-283,546, 1.3 µM (IC_{50})	[116]
Pyripyropene P (**249**)	ACAT inhibitor	Radioactive using enzyme rate liver microsomes	44.00 µM (IC_{50})	CL-283,546, 1.3 µM (IC_{50})	[116]
Pyripyropene Q (**250**)	ACAT inhibitor	Radioactive using enzyme rate liver microsomes	40.00 µM (IC_{50})	CL-283,546, 1.3 µM (IC_{50})	[116]
Pyripyropene R (**251**)	ACAT inhibitor	Radioactive using enzyme rate liver microsomes	78.00 µM (IC_{50})	CL-283,546, 1.3 µM (IC_{50})	[116]
Fumiparaphine A (**268**)	Antifungal	Broth microdilution/*Alternaria alternata*	2.0 µg/mL (MIC)	Nystatin, 2.0 µg/mL (MIC)	[128]
Helvolic acid (**273**)	Antifungal	Broth microdilution/*T. rubrum*	31.5 µg/mL (MIC),	Ketonazole, 31.5 µg/mL (MIC)	[42]
		Broth microdilution/*Botrytis cinerea*	6.25 µg/mL (MIC)	Carbendazim, 12.5 µg/mL (MIC)	[34]
		Broth microdilution/*Alternaria solani*	12.5 µg/mL (MIC)	Carbendazim, 12.5 µg/mL (MIC)	[34]
		Broth microdilution/*Alternaria alternata* (Fries) Keissler	6.25 µg/mL (MIC)	Carbendazim, 6.25 µg/mL (MIC)	[34]
		Broth microdilution/ *Colletotrichum gloeosporioides*	6.25 µg/mL Helvolic acid (MIC)	Carbendazim, 6.25 µg/mL (MIC)	[34]
		Broth microdilution/*Fusarium solani*	50.0 µg/mL (MIC)	Carbendazim, 25.0 µg/mL (MIC)	[34]

		Broth microdilution/*Fusarium oxysporum* f. sp. *niveum*	12.5 µg/mL (MIC)	Carbendazim, 12.5 µg/mL (MIC)	[34]
		Broth microdilution/*Fusarium oxysporum* f. sp. *vasinfectum*	25.0 µg/mL (MIC)	Carbendazim, 25.0 µg/mL (MIC)	[34]
		Broth microdilution/*Gibberella saubinettii.*	6.25 µg/mL (MIC)	Carbendazim, 6.25 µg/mL (MIC)	[34]
	Antibacterial	2-Fold dilution/*Streptococcus agalactiae*	8.0 µg/mL (MIC)	Tobramycin, 32.0 µg/mL (MIC)	[130]
16-O-Propionyl-16-O-deacetylhelvolic acid (**281**)	Antibacterial	2-Fold dilution/*Streptococcus agalactiae*	16.0 µg/mL (MIC)	Tobramycin, 32.0 µg/mL (MIC)	[130]
6-O-Propionyl-6-O-deacetylhelvolic acid (**282**)	Antibacterial	2-Fold dilution/*Streptococcus agalactiae*	2.0 µg/mL (MIC)	Tobramycin, 32.0 µg/mL (MIC)	[130]
24-*epi*-6β,16β-Diacetoxy-25-hydroxy-3,7-dioxo-29-nordammara-1,17(20)-diene-21,24-lactone (**286**)	Antibacterial	2-Fold dilution/*Streptococcus agalactiae*	64.0 µg/mL (MIC)	Tobramycin, 32.0 µg/mL (MIC)	[130]
22-*O*-Acetylisocyclocitrinol A (**294**)	Antibacterial	Broth microdilution/*A. baumanii* (ATCC 15122)	12.5 µg/mL (MIC)	Streptomycin, 12.5 µg/mL (MIC)	[70]
1-(8-Methoxy-3-methyl-1H-isochromen-6-yl)propan-1-one (**357**)	Antibacterial	Broth microdilution/*Pseudomonas syringae* pv. *angulata*	6.8 µg/mL (MIC)	Streptomycin, 2.2 µg/mL (MIC)	[141]
3-Hydroxy-1-(8- methoxy-3-methyl-1H-isochromen-7-yl)propan-1-one (**358**)	Antibacterial	Broth microdilution/*Pseudomonas syringae* pv. *angulata*	8.4 µg/mL (MIC)	Streptomycin, 2.2 µg/mL (MIC)	[141]
4,8-Dihydroxy-1-tetralone (**383**)	Antifungal	Broth microdilution/*Botrytis cinerea*	12.5 µg/mL (MIC)	Carbendazim, 12.5 µg/mL (MIC)	[34]
		Broth microdilution/*Alternaria solani*	12.5 µg/mL (MIC)	Carbendazim, 12.5 µg/mL (MIC)	[34]
		Broth microdilution/*Alternaria alternata* (Fries) Keissler	12.5 µg/mL (MIC)	Carbendazim, 6.25 µg/mL (MIC)	[34]
		Broth microdilution/ *Colletotrichum gloeosporioides*	12.5 µg/mL (MIC)	Carbendazim, 6.25 µg/mL (MIC)	[34]
		Broth microdilution/*Fusarium solani*	50.0 µg/mL (MIC)	Carbendazim, 25.0 µg/mL (MIC)	[34]
		Broth microdilution/*Fusarium oxysporum* f. sp. *niveum*	25.0 µg/mL (MIC)	Carbendazim, 12.5 µg/mL (MIC)	[34]

	Broth microdilution/*Fusarium oxysporum* f. sp. *vasinfectum*	50.0 µg/mL (MIC)	Carbendazim, 25.0 µg/mL (MIC)	[34]
	Broth microdilution/*Gibberella saubinettii.*	12.5 µg/mL (MIC)	Carbendazim, 6.25 µg/mL (MIC)	[34]
